# Cs_2_CO_3_-Promoted reaction of tertiary bromopropargylic alcohols and phenols in DMF: a novel approach to α-phenoxyketones

**DOI:** 10.3762/bjoc.18.44

**Published:** 2022-04-12

**Authors:** Ol'ga G Volostnykh, Olesya A Shemyakina, Anton V Stepanov, Igor' A Ushakov

**Affiliations:** 1 A. E. Favorsky Irkutsk Institute of Chemistry, Siberian Branch of the Russian Academy of Sciences, 1 Favorsky Str., 664033 Irkutsk, Russian Federation

**Keywords:** acetylenic alcohol, bromoacetylene, 1,3-dioxolan-2-one, phenols, phenoxyketone

## Abstract

The reaction of bromopropargylic alcohols with phenols in the presence of Cs_2_CO_3_/DMF affords α-phenoxy-α’-hydroxyketones (1:1 adducts) and α,α-diphenoxyketones (1:2 adducts) in up to 92% and 24% yields, respectively. Both products are formed via ring opening of the same intermediates, 1,3-dioxolan-2-ones, generated in situ from bromopropargylic alcohols and Cs_2_CO_3_.

## Introduction

Due to the relative stability, ease of handling and the presence of reactive sites, bromoacetylenes are widely applied in synthetic organic chemistry. They are known to be involved in various transformations including homo- and cross-coupling [1–7], addition [1,8–9], cycloaddition [1,10–11] and other reactions*.* Of particular synthetic value is the addition to the triple bond of bromoacetylenes to provide vinyl adducts, which can undergo numerous transformations. For example, bromoacetylenes were demonstrated to add imidazoles, imidazolines [12], and benzimidazoles [13–14] to give vinyl bromides. Sulfonamides reacted with bromoacetylenes to deliver *N*-bromovinyl-*p*-toluenesulfonamides that under Heck reaction conditions afforded *N*-(*p*-toluenesulfonyl)pyrroles [15]. The CsF-promoted nucleophilic addition of isocyanides to bromoacetylenes furnished the functionalized bromovinyl amides followed by Pd-catalyzed formation of 5-iminopyrrolone [16]. Sequential nucleophilic addition/intramolecular cyclization of amidine with bromoacetylenes led to imidazoles [17]. Also, M_2_CO_3_-catalyzed (M = K or Cs) addition of phenols to bromoacetylenes produced bromovinyl phenyl ethers, which were converted into 4*H*-chromen-4-ones, benzo[*b*]furans, etc. [18–21]. The latter reaction attracted our attention and prompted us to explore the interaction of phenols and bromopropargylic alcohols under the reported conditions. The bromopropargylic alcohols are readily available from acetylenic alcohols and hypobromite [22] or *N*-bromosuccinimide [23]. The presence of the hydroxy group expands the synthetic potential of these bromoacetylenes. Thus, we have recently demonstrated a highly selective hydration/acylation of tertiary bromopropargylic alcohols with carboxylic acids promoted by alkali metal carbonates [24]. The reaction proceeds via the ring-opening of 1,3-dioxolan-2-one intermediates formed with hydroxy and alkynyl groups of bromopropargylic alcohol and alkali metal carbonate. In the light of the above, it was unclear, in which direction would proceed the reaction of bromopropargylic alcohols and phenols. In the present paper, we report on the results of these studies.

## Results and Discussion

Initially, bromopropargylic alcohol **1a** and phenol (**2a**) were chosen as the model substrates for our investigation (Table 1). Completion of the reaction was monitored by IR and ^1^H NMR spectroscopy by the disappearance of the bands at 2196–2212 cm^–1^ (–C≡C–Br) and signals of the bromopropargylic alcohol **1a**, respectively. Under the conditions previously used [18–21] for the addition of phenols to bromoacetylenes (K_2_CO_3_ or Cs_2_CO_3_, DMF, 110 °C), the reaction turned out to be non-selective: along with the expected bromovinyl phenyl ether **3a** (3–9%) and phenoxyhydroxyketone **4a** (25–39%), diphenoxyketone **5a** was isolated in 9–24% yield (Table 1, entries 1–3). At 50–55 °C, the reaction slowed down and became more selective (Table 1, entries 4 and 5). With Cs_2_CO_3_ (1 equiv) at 50–55 °C, the reaction proceeded for 3 h, the yield of the phenoxyhydroxyketone **4a** increased up to 55% and 5-phenoxymethylene-1,3-dioxolan-2-one **7**, one of the probable intermediates, was isolated in 5% preparative yield (Table 1, entry 4), whereas the use of 2 equiv of Cs_2_CO_3_ led to slightly more selective reaction (Table 1, entry 5). Further lowering the temperature reduces the selectivity toward phenoxyketone **4a**. At room temperature, the full conversion of bromopropargylic alcohol **1a** took 15 h and yields of phenoxyketones **4a** and **5a** decreased (Table 1, entry 7). In the presence of K_2_CO_3_ (1 equiv) at 50–55 °C, the same reaction was completed for 8 h, the yields and selectivity being not improved (Table 1, entry 10). In these cases, 5-phenoxymethylene-1,3-dioxolan-2-one **7** was also isolated in 6–9% preparative yield. Hydrocarbonates CsHCO_3_ and KHCO_3_ were also tested in the reaction, which gave 5-bromomethylene-1,3-dioxolan-2-one **6a** as a major product in 29–36% yield (Table 1, entries 11 and 12). Considering that hydration occurs during the formation of phenoxyketone **4a**, we added water to the reaction system. It was shown that the reaction of **1a** with **2a** in aqueous DMF (1 equiv of Cs_2_CO_3_, DMF/H_2_O, 10:1, 50–55 °C) was highly selective to deliver phenoxyhydroxyketone **4a** in 78% yield and dihydroxyketone **8a** as a side product (Table 1, entry 6). Hence, the addition of phenol to the triple bond is a minor direction for the reaction of bromopropargylic alcohols and phenol in the presence of Cs_2_CO_3_/DMF, which was completely suppressed by addition of water. When DMF was replaced by DMSO (Table 1, entry 13), the preparative yield of reaction products decreased possibly due to product losses during extraction. No reaction was observed in CHCl_3_ (Table 1, entry 14) or utilizing organic bases (Et_3_N, DBU) (Table 1, entries 16–18). The efforts to increase the yield of diphenoxyketone **5a** using 2 equivalents of phenol (**2a**) in the reaction with bromopropargylic alcohol **1a** (Table 1, entries 8 and 9) failed.

**Table 1:** Screening of the conditions for reaction of bromopropargylic alcohol **1a** and phenol (**2a**)^a^.

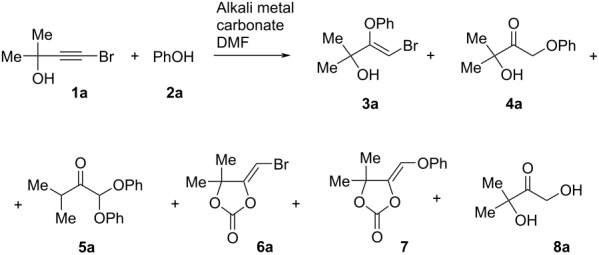									

Entry	Alkali metal carbonate (equiv)	*Т* (°С)	Time (h)	**3a** ^b^	**4a** ^b^	**5a** ^b^	**6a** ^b^	**7** ^b^	**8a** ^b^

1	K_2_CO_3_ (1)	110	1	9	31	21	–	–	–
2	Cs_2_CO_3_ (1)	110	1	3	39	24	–	–	–
3	Cs_2_CO_3_ (2)	110	1	9	25	9	–	–	–
4	Cs_2_CO_3_ (1)	50–55	3	4	55	22	–	5	–
5	Cs_2_CO_3_ (2)	50–55	3	–	44	24	–	–	–
6^c^	Cs_2_CO_3_ (1)	50–55	3	–	78	–	–	–	5
7	Cs_2_CO_3_ (1)	rt	15	4	29	16	–	6	–
8^d^	Cs_2_CO_3_ (1)	110	1	4	58	19	–	–	–
9^d^	Cs_2_CO_3_ (2)	110	1		17	9	–	–	–
10	K_2_CO_3_ (1)	50–55	8	–	30	10	–	9	–
11	KHCO_3_ (1)	110	1	–	8	5	29	–	–
12	CsHCO_3_ (1)	110	1	–	6	8	36	–	–
13^e^	Cs_2_CO_3_ (1)	50–55	3	–	25	18	–	9	–
14^f^	Cs_2_CO_3_ (1)	50–55	3	–	–	–	–	–	–
15	Na_2_CO_3_ (1)	110	1	–	–	–	–	–	–
16	Et_3_N	50–55	3	–	–	–	–	–	–
17	DBU	50–55	3	–	–	–	–	–	–
18	DBU	110	3	–	–	–	–	–	–

^a^Reaction conditions: **1a** (1.2 mmol), **2a** (1 mmol), alkali metal carbonate (1–2 equiv) in DMF (5 mL); the products were separated by column chromatography; ^b^Yields (%) are for the isolated products; ^c^In DMF/H_2_O (10:1); ^d^With 2 equiv of phenol; ^e^In DMSO; ^f^In CHCl_3_.

**Scheme 1:**
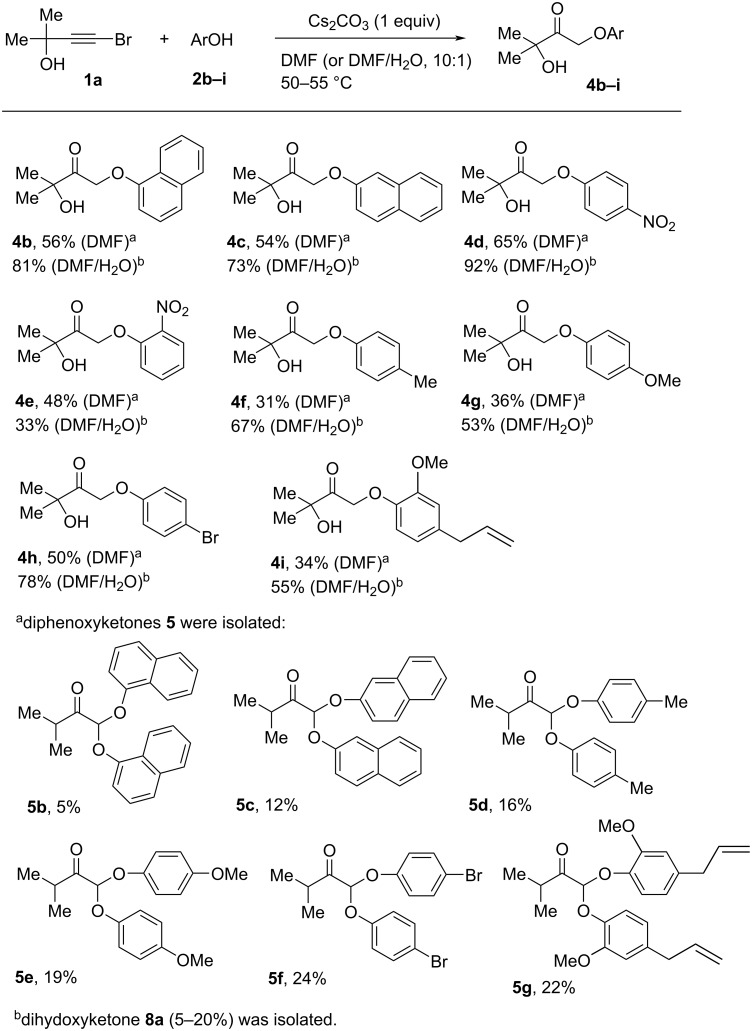
Scope of the reaction of bromopropargylic alcohol **1a** and phenols **2b**–**i**.

Employing the reaction conditions similar to those given in entries 4 and 6 (Table 1), we examined the substrate scope of the process relative to other phenols (Scheme 1). It was found that the electronic character of the substituents and the steric hindrance affected the reaction outcome. α-Naphthol (**2b**) and β-naphthol (**2c**) reacted with bromopropargylic alcohol **1a** in DMF or DMF/H_2_O to furnish naphthoxyhydroxyketones **4b**,**c** in preparative yields (up to 81%) comparable to those of **4a**. The introduction of an electron-withdrawing substituent (*p*-NO_2_) at the benzene ring gave a better result: *p*-nitrophenoxyhydroxyketone **4d** was formed in 65% (DMF) and 92% (DMF/H_2_O) isolated yields. However, the reaction of bromopropargylic alcohol **1a** with *o*-nitrophenol (**2e**) afforded *o*-nitrophenoxyhydroxyketone **4e** in only 48% (DMF) and 33% yields (DMF/H_2_O). The presence of an electron-donating group in *p*-cresol (**2f**), *p*-metoxyphenol (**2g**) and eugenol (**2i**) decreased the yields of phenoxyhydroxyketones **4f**,**g**, and **i** in comparison with phenol. Bromovinyl phenyl ethers **3** were not isolated. In DMF, diphenoxyketones **5b**–**g** were obtained in almost all the cases, the reaction with nitrophenols **2d**,**e** being the only exception. When the reactions of bromopropargylic alcohol **1a** with phenols **2b**–**i** were carried out in DMF/H_2_O, dihydroxyketone **8a** was isolated as a side product.

**Scheme 2:**
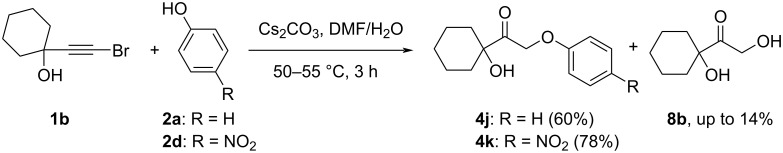
Reaction of bromopropargylic alcohol **1b** and phenols **2a** and **2d**.

Next, several experiments were carried out to evaluate the role of the steric effects of the alkyl substituents in bromopropargylic alcohols. The reaction of bromopropargylic alcohol **1b** bearing a cyclohexyl substituent with phenol (**2a**) in DMF/H_2_O (1 equiv of Cs_2_CO_3_, 50–55 °C, 3 h) gave phenoxyhydroxyketone **4j** in 60% yield (Scheme 2). Dihydroxyketone **8b** was isolated as side product in 5% yield. The reaction of bromopropargylic alcohol **1b** with *p*-nitrophenol (**2d**, DMF/H_2_O, 50–55 °C, 3 h) furnished product **8b** (14% yield) along with phenoxyhydroxyketone **4k** (78% isolated yield).

**Scheme 3:**
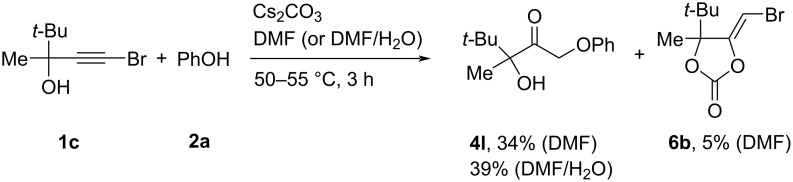
Reaction of bromopropargylic alcohol **1c** and phenol (**2a**).

Bromopropargylic alcohol **1c** having a *tert*-butyl group reacted with phenol (**2a**) in DMF for 3 h to give phenoxyhydroxyketone **4l** in only 34% yield, 5-bromomethylene-1,3-dioxolan-2-one **6b** (5%) being isolated (Scheme 3). In DMF/H_2_O (3 h), the conversion of **1c** was incomplete (50%) and phenoxyhydroxyketone **4l** was obtained in 39% yield. So, the steric hindrances of the bulky groups noticeably affect the reaction.

The reaction of secondary and primary bromopropargylic alcohols (4-bromobut-3-yn-2-ol and 3-bromoprop-2-yn-1-ol) and phenol (**2a**) with 1 equiv of Cs_2_CO_3_, DMF, 50–55 °C, for 3 h did not gave any products, the competitive polymerization of bromopropargylic alcohols **1** being predominant.

**Scheme 4:**
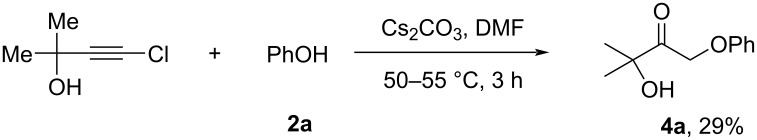
Reaction of chloropropargylic alcohol and phenol (**2a**).

Finally, chloroacetylenic alcohol was involved in the reaction with phenol (**2a**, 1 equiv Cs_2_CO_3_, DMF, 50–55 °C, 3 h) to afford the corresponding product **4a** in 29% isolated yield (Scheme 4).

**Scheme 5:**
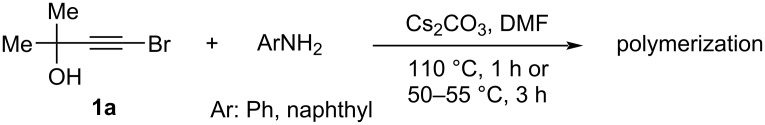
Reaction of bromopropargylic alcohol **1a** and anilines.

We tested aniline and 2-naphthylamine as nucleophiles (DMF, 50–55 °C) in the reaction of bromopropargylic alcohol **1a** (Scheme 5). But such a protocol turned out to be ineffective providing no desired products.

**Scheme 6:**
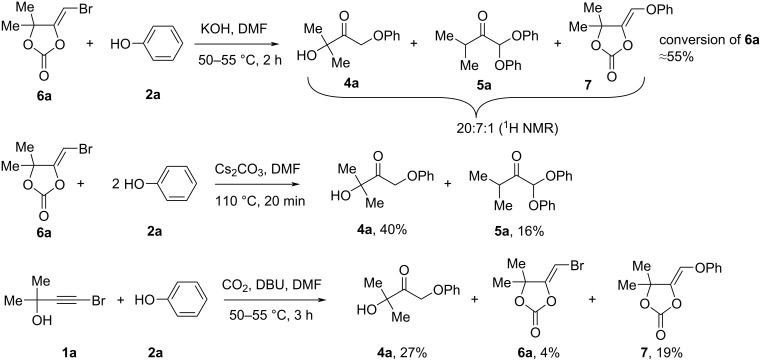
Control experiments.

Several control experiments were performed to gain insight into the reaction mechanism (Scheme 6). When the reaction of 5-bromomethylene-1,3-dioxolan-2-one **6a** and phenol (**2a**) was carried out with KOH, the conversion of the starting **6a** was 55% and crude product contained phenoxyketone **4a**, diphenoxyketone **5a** and 5-phenoxymethylene-1,3-dioxolan-2-one **7**. Using 2 equivalents of phenol (**2a**) in the reaction of 5-bromomethylene-1,3-dioxolan-2-one **6a** (Cs_2_CO_3_, DMF, 110 °C, 20 min) gave phenoxyketone **4a** and diphenoxyketone **5a** in 40 and 16% yields, correspondingly. These results confirm that compound **6a** is the main intermediate to form phenoxyketones. Next, we carried out the experiment using CO_2_ gas with DBU as a base. In comparison with reactions without CO_2_ (Table 1, entries 17 and 18), bromopropargylic alcohol **1a** with free CO_2_ gas in the presence of 100 mol % of DBU and phenol (**2a**) (DMF, 50–55 °C, 3 h) afforded phenoxyketone **4a**, 5-bromomethylene-1,3-dioxolan-2-one **6a** and 5-phenoxymethylene-1,3-dioxolan-2-one **7** in 27, 4 and 19% yields, respectively. This result suggest that Cs_2_CO_3_ acts as a source of CO_2_ for the formation of 5-bromomethylene-1,3-dioxolan-2-one **6a**.

**Scheme 7:**
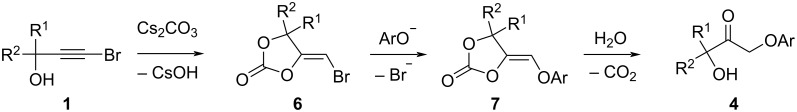
A plausible mechanism for the formation of phenoxyhydroxyketone **4**.

Obviously, the formation of phenoxyhydroxyketone **4** proceeds via 1,3-dioxolan-2-one **6** generated from bromopropargylic alcohol **1** and Cs_2_CO_3_. Then, Br-substitution/hydration of **6** and the release of CO_2_ give product **4** (Scheme 7).

**Scheme 8:**
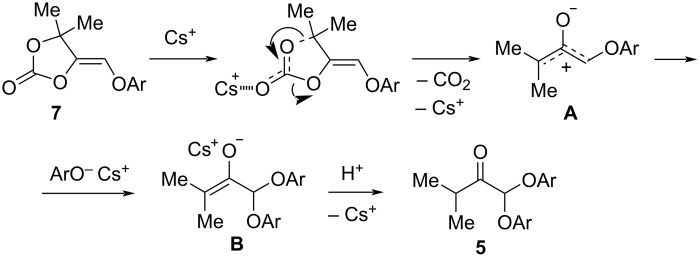
A plausible mechanism for the formation of diphenoxyketone **5**.

Apparently, diphenoxyketone **5** results from decarboxylative conversion of 1,3-dioxolan-2-one **7** leading to intermediate **A**, nucleophilic attack of phenolate at the less sterically hindered carbon of the above zwitterion **A** and subsequent protonation of anion **B** (Scheme 8).

Based on these plausible mechanisms for the formation of phenoxyketones, it can be assumed that a decline of the Cs^+^ concentration after Cs_2_CO_3_ convertion to CsBr (because of the very poor solubility of CsBr in DMF) has an influence on the rate of diphenoxyketone formation. In addition, the suppression of the di(nitrophenoxy)ketone formation can be due to the lower basicity of a reaction mixture since nitrophenols **2d**,**e** are more acidic than phenols **2a**–**c**,**f**–**i** (p*K*_a_ values: 9.99 [25–26] phenol (**2a**), 9.40 [27] α-naphthol (**2b**), 9.57 [27] β-naphthol (**2c**), 7.18 [25–26] *p*-nitrophenol (**2d**), 7.23 [25–26] *o*-nitrophenol (**2e**), 10.28 [25–26] *p*-cresol (**2f**), 10.27 [25–26] *p*-methoxyphenol (**2g**), 9.36 [25–26] *p*-bromophenol (**2h**), 10.19 eugenol (**2i**)). Addition of water to the reaction mixture also reduces the pH of the medium and simultaneously increases the concentration of hydroxide ions, therefore, diphenoxyketones **5** were not produced and dihydroxyketones **8** were formed as side products in these cases.

**Scheme 9:**
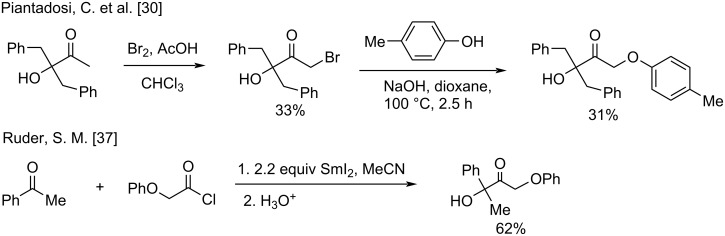
Examples of representative preparation of phenoxyketones **4**.

Among the approaches to produce α-phenoxyketones, the most common methodologies are base-catalyzed alkylation of the corresponding phenols with halo- [28–30] and mesyl [31–33] ketones (Scheme 9), the preparation of which are not always selective and high-yielded. The ring opening of ArOCH_2_-epoxides [34–35], the SmI_2_-catalyzed reductive coupling of acid halides with ketones [36–37] and acetolyses of α-phenoxy-α-diazoketones [38] were also employed.

**Scheme 10:**
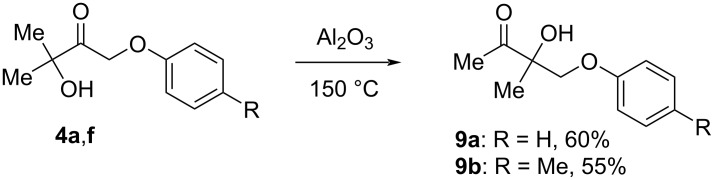
α-Ketol rearrangement of phenoxyketones **4a** and **4f**.

Recently, F. P. Cossío et al. [39] have described a method for the preparation of benzo[*b*]furans by thermal heating of a dispersion of α-phenoxyketones in Al_2_O_3_. We involved the synthesized α-phenoxyketones **4** in this reaction. The results showed that instead of benzo[*b*]furan formation, α-ketol rearrangement of phenoxyketones **4a**,**f** occurred to afford β-phenoxyketones **9a**,**b** in 55–60% yields (Scheme 10).

## Conclusion

We have shown that the main direction of the reaction of bromopropargylic alcohols and phenols in Cs_2_CO_3_/DMF is the hydration/phenoxylation of bromopropargylic alcohols to afford phenoxyketones. This step-economical process takes place under mild reaction conditions using simple readily available starting materials. The synthesized phenoxyketones are of interest as valuable building blocks for the production of other important molecules (e.g., amino alcohols, diols, etc.) [40–46] and potential pharmaceuticals. α-Hydroxyketones are structural subunits of natural products [47–49] and compounds possessing immunosuppressant [50], antidepressant [51], amyloid-β protein production inhibitory [52], urease inhibitory [53], farnesyl transferase inhibitory (kurasoin A and B) [54–55], antitumor and antibacterial (doxorubicin, olivomycin A, chromomycin A_3_, carminomycin I, epothilones) [56–58] activities.

## Experimental

### General information

^1^Н and ^13^С NMR spectra were recorded on a Bruker DPX-400 spectrometer (400.1 and 100.6 MHz, respectively) in CDCl_3_ or (CD_3_)_2_CO using hexamethyldisiloxane as internal reference at 20–25 °C. IR spectra were measured on a Varian 3100 FT-IR Excalibur series instrument as thin films or KВr pellets. Microanalyses were performed on a Flash 2000 elemental analyzer. Melting points were determined using a Kofler micro hot stage apparatus. Mass spectra were recorded on a GCMS-QP5050A spectrometer made by Shimadzu Company. Chromatographic column parameters were as follows: SPB^ТМ^-5, length 60 m, internal diameter 0.25 mm, thickness of stationary phase film 0.25 μm; injector temperature 250 °C, gas carrier – helium, flow rate 0.7 mL/min; detector temperature 250 °C; mass analyzer: quadrupole, electron ionization, electron energy: 70 eV, ion source temperature 200 °C; mass range 34–650 Da. The solvent was distilled DMF. Column chromatography was performed on silica gel 60 (230–400 mesh, particle size 0.040–0.063 mm, Merck). Bromopropargylic alcohols **1a**–**c** and chloropropargylic alcohol were prepared according to published methods [22–23,59]. Phenol (**2a**), naphthalen-1-ol (**2b**), naphthalen-2-ol (**2c**), 4-nitrophenol (**2d**), 2-nitrophenol (**2e**), *p*-cresol (**2f**), 4-methoxyphenol (**2g**), 4-bromophenol (**2h**), 4-allyl-2-methoxyphenol (**2i**) are commercial reagents. Commercially available starting materials were used without further purification. The structures of synthesized products have been proven by ^1^H, ^13^C and 2D (NOESY, ^1^Н,^13^С HSQC, ^1^Н,^13^С HMBC) NMR techniques, as well as IR spectra.

**Typical procedure for preparation of phenoxyhydroxyketones 4 in DMF, 50–55 °C.** To a stirred solution of Cs_2_CO_3_ (326 mg, 1 mmol) and phenol (**2a**; 94 mg, 1 mmol) in DMF (5 mL) 4-bromo-2-methylbut-3-yn-2-ol (**1a**; 196 mg, 1.2 mmol) was added dropwise. The reaction mixture was stirred at 50–55 °C for 3 h, filtered and concentrated. The residue was purified by flash column chromatography on silica gel (5.0 × 4.0 cm, gradient elution, C_6_H_14_/Et_2_O, 2:1 followed by Et_2_O, Me_2_CO) to give products **3a** (10 mg, 4%), **4a** (214 mg, 55%), **5a** (30 mg, 22%) and **7** (11 mg, 5%).

## Supporting Information

File 1 General information, synthetic procedures and additional optimization results, NMR spectra and characterization of synthesized compounds.
