# Supplementary material for: Cs2CO3-Promoted reaction of tertiary bromopropargylic alcohols and phenols in DMF: a novel approach to α-phenoxyketones
Source: Beilstein J Org Chem. 2022 Apr 12;18:420–8. doi: 10.3762/bjoc.18.44 (PMC9039521; doi:10.3762/bjoc.18.44)

BEILSTEIN JOURNAL OF ORGANIC CHEMISTRY

# Supporting Information

for

# $Cs_2CO_3$-Promoted reaction of tertiary bromopropargylic alcohols and phenols in DMF: a novel approach to α-phenoxyketones

Ol'ga G. Volostnykh, Olesya A. Shemyakina, Anton V. Stepanov and Igor' A. Ushakov

*Beilstein J. Org. Chem.* **2022,** *18,* 420–428. doi:10.3762/bjoc.18.44

# General information, synthetic procedures and additional optimization results, NMR spectra and characterization of synthesized compounds

License and Terms: This is a supporting information file under the terms of the Creative Commons Attribution License (https://creativecommons.org/licenses/by/4.0). Please note that the reuse, redistribution and reproduction in particular requires that the author(s) and source are credited and that individual graphics may be subject to special legal provisions.
The license is subject to the *Beilstein Journal of Organic Chemistry* terms and conditions: (https://www.beilstein-journals.org/bjoc/terms)

# Table of contents


1. General Information ..... S2

2. Method A. Typical procedure for preparation of phenoxyhydroxyketones **4** in DMF, 50–55 °C ..... S2

3. Method B. Typical procedure for preparation of phenoxyhydroxyketones **4** in DMF/$H_2O$, 50–55 °C ..... S2

4. Method C. Typical procedure for preparation of phenoxyhydroxyketones **4** in DMF, 110 °C S3

5. Method D. Typical procedure for preparation of phenoxyhydroxyketones **4** in DMF, 20–25 °C ..... S3

6. Optimization of reaction conditions. Yields of the synthesized products. ..... S3

7. Characterization data for all products ..... S4

References ..... S10

8. Rearrangement of the synthesized phenoxyhydroxyketones **4a**,**f** ..... S10

9. Characterization data for **9a**, **9b** ..... S10

10. $^{1}H$ and $^{13}C$ spectra of the synthesized products ..... S11

4-Bromo-2-methyl-3-phenoxybut-3-en-2-ol (**3a**) ..... S11

3-Hydroxy-3-methyl-1-phenoxybutan-2-one (**4a**) ..... S12

3-Hydroxy-3-methyl-1-(naphthalen-1-yloxy)butan-2-one (**4b**) ..... S13

3-Hydroxy-3-methyl-1-(naphthalen-2-yloxy)butan-2-one (**4c**) ..... S14

3-Hydroxy-3-methyl-1-(4-nitrophenoxy)butan-2-one (**4d**) ..... S15

3-Hydroxy-3-methyl-1-(2-nitrophenoxy)butan-2-one (**4e**) ..... S16

3-Hydroxy-3-methyl-1-(p-tolyloxy)butan-2-one (**4f**) ..... S17

3-Hydroxy-1-(4-methoxyphenoxy)-3-methylbutan-2-one (**4g**) ..... S18

1-(4-Bromophenoxy)-3-hydroxy-3-methylbutan-2-one (**4h**) ..... S19

1-(4-Allyl-2-methoxyphenoxy)-3-hydroxy-3-methylbutan-2-one (**4i**) ..... S20

1-(1-Hydroxycyclohexyl)-2-phenoxyethan-1-one (**4j**) ..... S21

1-(1-Hydroxycyclohexyl)-2-(4-nitrophenoxy)ethan-1-one (**4k**) ..... S22

3-Hydroxy-3,4,4-trimethyl-1-phenoxypentan-2-one (**4l**) ..... S23

3-Methyl-1,1-diphenoxybutan-2-one (**5a**) ..... S24

1,1-Bis(4-methoxyphenoxy)-3-methylbutan-2-one (**5e**) ..... S25

1,1-Bis(4-bromophenoxy)-3-methylbutan-2-one (**5f**) ..... S26

1,1-Bis(4-allyl-2-methoxyphenoxy)-3-methylbutan-2-one (**5g**) ..... S27

(Z)-4,4-Dimethyl-5-(phenoxymethylene)-1,3-dioxolan-2-one (**7**) ..... S28

2D $^{1}H$-$^{13}C$ HMBC Spectrum of **7** ($CDCl_3$) ..... S29

2D NOESY Spectrum of **7** ($CDCl_3$) ..... S30

3-Hydroxy-3-methyl-4-phenoxybutan-2-one (**9a**) ..... S31

3-Hydroxy-3-methyl-4-(*p*-tolyloxy)butan-2-one (**9b**) ..... S32

## 1. General Information

$^1H$ and $^{13}C$ NMR spectra were recorded on a Bruker DPX-400 spectrometer (400.1 and 100.6 MHz, respectively) in $CDCl_3$ or $(CD_3)_2CO$ using hexamethyldisiloxane as internal references at 20-25 °C.
IR spectra were measured on a Varian 3100 FT-IR Excalibur series instrument as thin films or KBr pellets. Microanalyses were performed on a Flash 2000 elemental analyzer. Melting points were determined using a Kofler micro hot stage. Mass spectra were recorded on a GCMS-QP5050A spectrometer made by Shimadzu Company. Chromatographic column parameters were as follows: SPB$^{TM}$-5, length 60 m, internal diameter 0.25 mm, thickness of stationary phase film 0.25 $\mu$m; injector temperature 250 °C, gas carrier – helium, flow rate 0.7 mL/min; detector temperature 250 °C; mass analyzer: quadrupole, electron ionization, electron energy: 70 eV, ion source temperature 200 °C; mass range 34-650 Da. The solvent was distilled DMF. Column chromatography was performed on silica gel 60 (230-400 mesh, particle size 0.040-0.063 mm, Merck). Bromopropargylic alcohols **1a**–**c** and chloropropargylic alcohol were prepared according to published methods [1-3]. Phenol (**2a**), naphthalen-1-ol (**2b**), naphthalen-2-ol (**2c**), 4-nitrophenol (**2d**), 2-nitrophenol (**2e**), 4-methylphenol (**2f**), 4-methoxyphenol (**2g**), 4-bromophenol (**2h**), 4-allyl-2-methoxyphenol (**2i**) are commercial reagents. Commercially available starting materials were used without further purification. The structures of synthesized products have been proven by $^1H$, $^{13}C$ and 2D (NOESY, $^1H$-$^{13}C$ HSQC, $^1H$-$^{13}C$ HMBC) NMR techniques, as well as IR spectra.

## 2. Method A. Typical procedure for preparation of phenoxyhydroxyketones 4 in DMF, 50–55 °C

To a stirred solution of $Cs_2CO_3$ (326 mg, 1 mmol) and phenol (**2a**; 94 mg, 1 mmol) in DMF (5 mL) 4-bromo-2-methylbut-3-yn-2-ol (**1a**; 196 mg, 1.2 mmol) was added dropwise. The reaction mixture was stirred at 50-55 °C for 3 h, filtered and concentrated. The residue was purified by flash column chromatography on silica gel (5.0 × 4.0 cm, gradient elution, $C_6H_{14}$–$Et_2O$, 2:1 followed by $Et_2O$, $Me_2CO$) to give 4-bromo-2-methyl-3-phenoxybut-3-en-2-ol (**3a**) (10 mg, 4%), 3-hydroxy-3-methyl-1-phenoxybutan-2-one (**4a**) (214 mg, 55%), 3-methyl-1,1-diphenoxybutan-2-one (**5a**) (30 mg, 22%) and (*Z*)-4,4-dimethyl-5-(phenoxymethylene)-1,3-dioxolan-2-one (**7**) (11 mg, 5%).

## 3. Method B. Typical procedure for preparation of phenoxyhydroxyketones 4 in DMF/$H_2O$, 50–55 °C

To a stirred solution of $Cs_2CO_3$ (326 mg, 1 mmol) and phenol (**2a**; 94 mg, 1 mmol) in distilled $H_2O$ (0.5 mL) and DMF (5 mL) 4-bromo-2-methylbut-3-yn-2-ol (**1a**; 196 mg, 1.2 mmol) was added dropwise. The reaction mixture was stirred at 50–55 °C for 3 h. The mixture was filtered and concentrated, this gave mixture of the 3-hydroxy-3-methyl-1-phenoxybutan-2-one (**4a**) and 1,3-dihydroxy-3-methylbutan-2-one (**8a**). The mixture of the **4a** and **8a** was purified by flash column chromatography on silica gel (5.0 × 4.0 cm, gradient elution, $C_6H_{14}$–$Et_2O$, 2:1 followed by $Et_2O$, $Me_2CO$) to give products **4a** (152 mg, 78%) and **8a** (6 mg, 5%).

## 4. Method C. Typical procedure for preparation of phenoxyhydroxyketones 4 in DMF, 110 °C

To a stirred solution of carbonate [1 mmol ($K_2CO_3$, 138 mg or $Cs_2CO_3$, 326 mg)] and phenol (**2a**; 94 mg, 1 mmol) in DMF (5 mL) 4-bromo-2-methylbut-3-yn-2-ol (**1a**; 196 mg, 1.2 mmol) was added dropwise. The reaction mixture was stirred at 110 °C for 1 h. The mixture was filtered and concentrated, this gave mixture of the 4-bromo-2-methyl-3-phenoxybut-3-en-2-ol (**3a**), 3-hydroxy-3-methyl-1-phenoxybutan-2-one (**4a**) and 3-methyl-1,1-diphenoxybutan-2-one (**5a**). The mixture of the **3a**, **4a** and **5a** was purified by flash column chromatography on silica gel (5.0 × 4.0 cm, gradient elution, $C_6H_{14}$–$Et_2O$, 2:1 followed by $Et_2O$, $Me_2CO$) to give products **3a** (23 mg, 9% for $K_2CO_3$; 8 mg, 3% for $C_2CO_3$), **4a** (60 mg, 31% for $K_2CO_3$; 75 mg, 39% for $C_2CO_3$) and **5a** (28 mg, 21% for $K_2CO_3$; 33 mg, 24% for $C_2CO_3$).

## 5. Method D. Typical procedure for preparation of phenoxyhydroxyketones 4 in DMF, 20–25 °C

To a stirred solution of $Cs_2CO_3$ (326 mg, 1 mmol) and phenol (**2a**; 94 mg, 1 mmol) in DMF (5 mL) 4-bromo-2-methylbut-3-yn-2-ol (**1a**; 196 mg, 1.2 mmol) was added dropwise. The reaction mixture was stirred at 20-25 °C for 15 h. The mixture was filtered and concentrated, this gave mixture of the 4-bromo-2-methyl-3-phenoxybut-3-en-2-ol (**3a**), 3-hydroxy-3-methyl-1-phenoxybutan-2-one (**4a**), 3-methyl-1,1-diphenoxybutan-2-one (**5a**), (*Z*)-5-(bromomethylene)-4,4-dimethyl-1,3-dioxolan-2-one (**6a**) and (*Z*)-4,4-dimethyl-5-(phenoxymethylene)-1,3-dioxolan-2-one (**7**). The mixture of the **3a**, **4a**, **5a**, **6a** and **7** was purified by flash column chromatography on silica gel (5.0 × 4.0 cm, gradient elution, $C_6H_{14}$–$Et_2O$, 2:1 followed by $Et_2O$, $Me_2CO$) to give products **3a** (10 mg, 4%), **4a** (56 mg, 29%), **5a** (21 mg, 16%) and **7** (13 mg, 6%).

## 6. Optimization of reaction conditions. Yields of the synthesized products.

a) Following **Method A** to a stirred solution of $Cs_2CO_3$ (652 mg, 2 mmol) and phenol (**2a**; 94 mg, 1 mmol) in DMF (5 mL) 4-bromo-2-methylbut-3-yn-2-ol (**1a**; 196 mg, 1.2 mmol) was added dropwise. The reaction mixture was stirred at 50-55 °C for 3 h. After purification the desired products **4a** (85 mg, 44%) and **5a** (33 mg, 24%) were obtained.
b) Following **Method A** to a stirred solution of $K_2CO_3$ (138 mg, 1 mmol) and phenol (**2a**; 94 mg, 1 mmol) in DMF (5 mL) 4-bromo-2-methylbut-3-yn-2-ol (**1a**; 196 mg, 1.2 mmol) was added dropwise. The reaction mixture was stirred at 50-55 °C for 8 h. After purification the desired products **4a** (58 mg, 30%), **5a** (13 mg, 10%) and **7** (20 mg, 9%) were obtained.
c) Following **Method A** to a stirred solution of $CsHCO_3$ (194 mg, 1 mmol) and phenol (**2a**; 94 mg, 1 mmol) in DMF (5 mL) 4-bromo-2-methylbut-3-yn-2-ol (**1a**; 196 mg, 1.2 mmol) was added dropwise. The reaction mixture was stirred at 50-55 °C for 9 h. After purification the desired products **4a** (35 mg, 18%), **6a** (56 mg, 27%) and **7** (33 mg, 15%) were obtained.
d) Following **Method C** to a stirred solution of $Cs_2CO_3$ (652 mg, 2 mmol) and phenol (**2a**; 94 mg, 1 mmol) in DMF (5 mL) 4-bromo-2-methylbut-3-yn-2-ol (**1a**; 196 mg, 1.2 mmol) was added dropwise. The reaction mixture was stirred at 110 °C for 1 h. After purification the desired products **3a** (23 mg, 9%), **4a** (49 mg, 25%) and **5a** (12 mg, 9%) were obtained.
e) Following **Method C** to a stirred solution of $CsHCO_3$ (194 mg, 1 mmol) and phenol (**2a**; 94 mg, 1 mmol) in DMF (5 mL) 4-bromo-2-methylbut-3-yn-2-ol (**1a**; 196 mg, 1.2 mmol) was added dropwise. The reaction mixture was stirred at 110 °C for 1 h. After purification the desired products **4a** (12 mg, 6%), **5a** (11 mg, 8%) and **6a** (74 mg, 36%) were obtained.
f) Following **Method C** to a stirred solution of $KHCO_3$ (194 mg, 1 mmol) and phenol (**2a**; 94 mg, 1 mmol) in DMF (5 mL) 4-bromo-2-methylbut-3-yn-2-ol (**1a**; 196 mg, 1.2 mmol) was added

dropwise. The reaction mixture was stirred at 110 °C for 1 h. After purification the desired products **4a** (15 mg, 8%), **5a** (7 mg, 5%) and **6a** (59 mg, 29%) were obtained.
g) To a stirred solution of $CsHCO_3$ (194 mg, 1 mmol) in DMF (5 mL) 4-bromo-2-methylbut-3-yn-2-ol (**1a**; 196 mg, 1.2 mmol) was added dropwise. The reaction mixture was stirred at 110 °C for 1 h. The mixture was filtered and concentrated, this gave (*Z*)-5-(bromomethylene)-4,4-dimethyl-1,3-dioxolan-2-one (**6a**). The product **6a** was purified by flash column chromatography on silica gel (5.0 × 4.0 cm, gradient elution, $C_6H_{14}$–$Et_2O$, 2:1 followed by $Et_2O$, $Me_2CO$) to give the desired product **6a** (65 mg, 31%).

## 7. Characterization data for all products

**4-Bromo-2-methyl-3-phenoxybut-3-en-2-ol (3a)**
Yield 10 mg, 4% (Method A). White solid; mp 68-70 °C.
IR (KBr): 3115, 3057, 2994, 2972, 2928, 2864, 1633, 1592, 1489, 1457, 1422, 1360, 1291, 1260, 1214, 1202, 1177, 1163, 1129, 1077, 1022, 970, 900, 775, 754, 746, 735, 688, 647, 546, 498 $cm^{-1}$.
$^1H$ NMR (400.1 MHz, $CDCl_3$): δ = 7.28-7.26 (m, 2 H, Ph), 6.99-6.97 (m, 3 H, Ph), 6.32 (s, 1 H, CH), 1.93 (s, 1 H, OH), 1.43 [s, 6 H, $(CH_3)_2$].
$^{13}C$ NMR (100.6 MHz, $CDCl_3$): δ = 159.0 (CH*C*O), 156.0, 130.0, 122.2, 115.7 (Ph), 93.8 (CH), 73.9 (C–OH), 28.3 [$(CH_3)_2$].
MS (EI): *m/z* (%) = 258 (22) $[M+1]^+$, 256 (23), 241 (10), 162 (11), 161 (14), 119 (29), 118 (14), 95 (11), 94 (82), 91 (44), 90 (12), 81 (11), 77 (74), 66 (15), 65 (32), 59 (100), 51 (38), 43 (96), 41 (16), 39 (40).
Anal. Calcd for $C_{11}H_{13}BrO_2$ (257.13): C, 51.38; H, 5.10; Br, 31.08. Found: C, 51.37; H, 5.12; Br, 31.05.

**3-Hydroxy-3-methyl-1-phenoxybutan-2-one (4a)**
Yield 107 mg, 55% (Method A); Yield 152 mg, 78% (Method B). White solid; mp 66-67 °C.
IR (KBr): 3066, 2979, 2922, 2876, 1727, 1597, 1496, 1414, 1357, 1295, 1250, 1185, 1142, 1040, 966, 880, 840, 754, 690, 611, 554, 505, 425 $cm^{-1}$.
$^1H$ NMR (400.1 MHz, $CDCl_3$): δ = 7.32-7.25 (m, 2 H, Ph), 7.00-6.96 (m, 1 H, Ph), 6.92-6.86 (m, 2 H, Ph), 4.97 (s, 2 H, $CH_2$), 3.15 (s, 1 H, OH), 1.46 [s, 6 H, $(CH_3)_2$].
$^{13}C$ NMR (100.6 MHz, $CDCl_3$): δ = 209.2 (C=O), 157.8, 130.0, 121.9, 114.7 (Ph), 76.6 (C–OH), 69.2 ($CH_2$), 26.9 [$(CH_3)_2$].
MS (EI): *m/z* (%) = 194 (11) [$M^+$], 152 (10), 151 (96), 133 (60), 107 (21), 105 (35), 95 (24), 94 (77), 79 (17), 77 (49), 66 (11), 65 (16), 57 (55), 51 (23), 43 (100), 39 (15).
Anal. Calcd for $C_{11}H_{14}O_3$ (194.23): C, 68.02; H, 7.27. Found: C, 68.00; H, 7.30.

**3-Hydroxy-3-methyl-1-(naphthalen-1-yloxy)butan-2-one (4b)**
Yield 137 mg, 56% (Method A); Yield 198 mg, 81% (Method B). Brown solid; mp 87-90 °C.
IR (KBr): 3056, 2976, 2922, 2870, 1721, 1629, 1579, 1507, 1464, 1400, 1355, 1277, 1231, 1183, 1143, 1056, 1017, 962, 869, 769, 712, 591, 569, 543, 521, 492 $cm^{-1}$.
$^1H$ NMR (400.1 MHz, $CDCl_3$): δ = 8.34-8.32 (m, 1 H, Naphthyl), 7.80-7.78 (m, 1 H, Naphthyl), 7.53-7.42 (m, 3 H, Naphthyl), 7.31 (t, $J$ = 8.0 Hz, 1 H, Naphthyl), 6.66 (d, $J$ = 7.6 Hz, 1 H, Naphthyl), 5.13 (s, 2 H, $CH_2$), 3.19 (s, 1 H, OH), 1.50 [s, 6 H, $(CH_3)_2$].
$^{13}C$ NMR (100.6 MHz, $CDCl_3$): δ = 208.9 (C=O), 153.7, 134.7, 127.6, 126.7, 125.7, 125.5, 122.1, 121.6, 105.2 (Naphthyl), 76.6 (C–OH), 69.5 ($CH_2$), 27.0 [$(CH_3)_2$].
MS (EI): *m/z* (%) = 185 (45) $[M – Me_2COH]^+$, 158 (95), 157 (17), 144 (100), 130 (15), 129 (13), 127 (71), 125 (50), 115 (43), 114 (16), 79 (11), 75 (16), 65 (24), 64 (27), 59 (81), 57 (17), 55 (25), 51 (19), 41 (23).
Anal. Calcd for $C_{15}H_{16}O_3$ (244.29): C, 73.75; H, 6.60. Found: C, 73.71; H, 6.58.

**3-Hydroxy-3-methyl-1-(naphthalen-2-yloxy)butan-2-one (4c)**
Yield 132 mg, 54% (Method A); Yield 177 mg, 73% (Method B). White solid; mp 131-133 °C.
IR (KBr): 3057, 2979, 2933, 2911, 1816, 1727, 1630, 1600, 1510, 1470, 1440, 1412, 1391, 1359, 1261, 1220, 1183, 1146, 1097, 1037, 968, 842, 816, 742, 584, 476 $cm^{-1}$.
$^1$H NMR (400.1 MHz, $CDCl_3$): δ =7.76 (d, *J* = 8.6 Hz, 2 H, Naphthyl), 7.69 (d, *J* = 8.1 Hz, 1 H, Naphthyl), 7.45-7.33 (m, 2 H, Naphthyl), 7.27-7.19 (m, 1 H, Naphthyl), 7.06-7.05 (m, 1 H, Naphthyl), 5.08 (s, 2 H, $CH_2$), 3.08 (s, 1 H, OH), 1.51 [s, 6 H, $(CH_3)_2$].
$^{13}$C NMR (100.6 MHz, $CDCl_3$): δ = 208.8 (C=O), 155.8, 134.3, 129.9, 129.5, 127.8, 126.9, 126.7, 124.3, 118.6, 107.3 (Naphthyl), 76.7 (C–OH), 69.3 ($CH_2$), 27.0 [$(CH_3)_2$].
MS (EI): *m/z* (%) = 244 (26) [$M^+$], 201 (35), 183 (37), 157 (11), 145 (15), 144 (100), 143 (19), 129 (12), 127 (31), 116 (17), 115 (87), 43 (72).
Anal. Calcd for $C_{15}H_{16}O_3$ (244.29): C, 73.75; H, 6.60. Found: C, 73.73; H, 6.62.

**3-Hydroxy-3-methyl-1-(4-nitrophenoxy)butan-2-one (4d)**
Yield 156 mg, 65% (Method A); Yield 221 mg, 92% (Method B). Colorless crystals; mp 68-71 °C.
IR (KBr): 3083, 2979, 2936, 2897, 1731, 1593, 1507, 1465, 1338, 1271, 1240, 1187, 1109, 1030, 961, 850, 751, 690, 635, 574, 532, 505, 470 $cm^{-1}$.
$^1$H NMR [400.1 MHz, $(CD_3)_2CO$]: δ = 8.23-8.13 (m, 2 H, Ph), 7.10-6.99 (m, 2 H, Ph), 5.43 (s, 2 H, $CH_2$), 4.66 (s, 1 H, OH), 1.38 [s, 6 H, $(CH_3)_2$].
$^{13}$C NMR [100.6 MHz, $(CD_3)_2CO$]: δ = 209.1 (C=O), 164.3, 142.2, 126.2, 115.4 (Ph), 77.1 (C–OH), 69.9 ($CH_2$), 26.8 [$(CH_3)_2$].
MS (EI): *m/z* (%) = 152 (77) [M – (COC(OH)$Me_2$)]$^+$, 76 (11), 59 (100), 43 (10).
Anal. Calcd for $C_{11}H_{13}NO_5$ (239.23): C, 55.23; H, 5.48; N, 5.86. Found: C, 55.25; H, 5.45; N, 5.87.

**3-Hydroxy-3-methyl-1-(2-nitrophenoxy)butan-2-one (4e)**
Yield 114 mg, 48% (Method A); Yield 78 mg, 33% (Method B). Yellow oil.
IR (film): 3085, 2980, 2932, 2876, 1734, 1606, 1526, 1487, 1426, 1355, 1287, 1240, 1137, 1087, 1024, 964, 858, 774, 746, 700, 664, 557, 518 $cm^{-1}$.
$^1$H NMR [400.1 MHz, $(CD_3)_2CO$]: δ = 7.84-7.78 (m, 1 H, Ph), 7.57-7.52 (m, 1 H, Ph) 7.13-7.05 (m, 2 H, Ph), 5.44 (s, 2 H, $CH_2$), 4.68 (s, 1 H, OH), 1.36 [s, 6 H, $(CH_3)_2$].
$^{13}$C NMR [100.6 MHz, $(CD_3)_2CO$]: δ = 208.9 (C=O), 151.7, 141.0, 134.1, 125.4, 121.3, 115.4 (Ph), 77.0 (C–OH), 70.4 ($CH_2$), 26.7 [$(CH_3)_2$].
MS (EI): *m/z* (%) = 123 (42) [M – ($Me_2C(OH)COCH_2O$)]$^+$, 93 (12), 77 (12), 59 (100), 43 (12), 41 (15), 39 (14).
Anal. Calcd for $C_{11}H_{13}NO_5$ (239.23): C, 55.23; H, 5.48; N, 5.86. Found: C, 55.20; H, 5.47; N, 5.89.

**3-Hydroxy-3-methyl-1-(*p*-tolyloxy)butan-2-one (4f)**
Yield 65 mg, 31% (Method A); Yield 139 mg, 67% (Method B). Colorless crystals; mp 86-88 °C.
IR (KBr): 2987, 2975, 2929, 2902, 2871, 1729, 1613, 1588, 1512, 1465, 1424, 1356, 1292, 1247, 1181, 1143, 1107, 1039, 968, 814, 581, 498 $cm^{-1}$.
$^1$H NMR (400.1 MHz, $CDCl_3$): δ = 7.07 (d, *J* = 8.0 Hz, 2 H, Ph), 6.78 (d, *J* = 8.0 Hz, 2 H, Ph), 4.93 (s, 2 H, $CH_2$), 3.01 (s, 1 H, OH), 2.27 (s, 3 H, $CH_3$), 1.45 [s, 6 H, $(CH_3)_2$].
$^{13}$C NMR (100.6 MHz, $CDCl_3$): δ = 209.3 (C=O), 155.8, 131.3, 130.1, 114.6 (Ph) , 76.5 (C–OH), 69.5 ($CH_2$), 26.9 [$(CH_3)_2$], 20.5 ($CH_3$).
MS (EI): *m/z* (%) = 208 (13) [$M^+$], 150 (18), 122 (66), 121 (31), 109 (10), 108 (79), 107 (32), 92 (16), 91 (44), 77 (19), 65 (30), 60 (10), 59 (100), 43 (24), 41 (17), 39 (15).
Anal. Calcd for $C_{12}H_{16}O_3$ (208.26): C, 69.21; H, 7.74. Found: C, 69.19; H, 7.76.

**3-Hydroxy-1-(4-methoxyphenoxy)-3-methylbutan-2-one (4g)**
Yield 80 mg, 36% (Method A); Yield 118 mg, 53% (Method B). Colorless crystals; mp 98-102 °C.
IR (KBr): 3072, 3049, 3000, 2972, 2934, 2909, 2842, 1725, 1630, 1510, 1469, 1449, 1414, 1373, 1358, 1348, 1291, 1237, 1183, 1138, 1112, 1040, 965, 827, 795, 721, 584, 549, 529 $cm^{-1}$.
$^1H$ NMR (400.1 MHz, $CDCl_3$): δ = 6.86-6.80 (m, 4 H, Ph), 4.91 (s, 2 H, $CH_2$), 3.75 (s, 3 H, $OCH_3$), 3.14 (s, 1 H, OH), 1.45 [s, 6 H, $(CH_3)_2$].
$^{13}C$ NMR (100.6 MHz, $CDCl_3$): δ = 209.4 (C=O), 154.7, 152.0, 116.0, 114.8 (Ph), 76.5 (C–OH), 70.3 ($CH_2$), 55.8 ($OCH_3$), 26.9 [$(CH_3)_2$].
Anal. Calcd for $C_{12}H_{16}O_4$ (224.26): C, 64.27; H, 7.19. Found: C, 64.30; H, 7.18.

**1-(4-Bromophenoxy)-3-hydroxy-3-methylbutan-2-one (4h)**
Yield 136 mg, 50% (Method A); Yield 213 mg, 78% (Method B). White solid; mp 95-98 °C.
IR (KBr): 3019, 2978, 2935, 2851, 1732, 1639, 1594, 1511, 1464, 1421, 1367, 1264, 1216, 1146, 1127, 1104, 1033, 1000, 962, 919, 851, 756, 668 $cm^{-1}$.
$^1H$ NMR (400.1 MHz, $CDCl_3$): δ = 7.39-7.34 (m, 2 H, Ph), 6.78-6.74 (m, 2 H, Ph), 4.97 (s, 2 H, $CH_2$), 2.99 (s, 1 H, OH), 1.45 [s, 6 H, $(CH_3)_2$].
$^{13}C$ NMR (100.6 MHz, $CDCl_3$): δ = 208.7 (C=O), 157.0, 132.5, 116.6, 114.1 (Ph), 76.7 (C–OH), 69.2 ($CH_2$), 27.0 [$(CH_3)_2$].
Anal. Calcd for $C_{11}H_{13}BrO_3$ (273.13): C, 48.37; H, 4.80; Br, 29.26. Found: C, 48.36; H, 4.76; Br, 29.29.

**1-(4-Allyl-2-methoxyphenoxy)-3-hydroxy-3-methylbutan-2-one (4i)**
Yield 89 mg, 34% (Method A); Yield 145 mg, 55% (Method B). Colorless crystals; mp 61-63 °C.
IR (KBr): 3019, 2978, 2935, 2919, 2851, 1732, 1639, 1594, 1511, 1464, 1421, 1367, 1264, 1216, 1146, 1127, 1104, 1033, 1000, 962, 919, 756, 668 $cm^{-1}$.
$^1H$ NMR (400.1 MHz, $CDCl_3$): δ = 6.74-6.66 (m, 3 H, Ph), 5.98-5.88 (m, 1 H, $CH_2CH$), 5.09-5.03 (m, 2 H, $=CH_2$), 4.94 (s, 2 H, $CH_2$), 3.85 (s, 3 H, $OCH_3$), 3.52 (s, 1 H, OH), 3.32 (d, $J$ = 6.8 Hz, 1 H, $CH_2CH$), 1.44 [s, 6 H, $(CH_3)_2$].
$^{13}C$ NMR (100.6 MHz, $CDCl_3$): δ = 209.2 (C=O), 149.7, 145.6 (Ph), 137.4 ($CH=CH_2$), 134.8, 120.6 (Ph), 115.9 ($=CH_2$), 114.9, 112.6 (Ph), 71.6 (C–OH), 55.9 ($CH_2$), 39.9 ($OCH_3$), 26.7 [$(CH_3)_2$].
MS (EI): $m/z$ (%) = 264 (5) [$M^+$], 178 (27), 164 (40), 163 (18), 147 (13), 115 (13), 103 (10), 91 (15), 77 (13), 59 (100), 43 (18), 41 (21), 39 (12).
Anal. Calcd for $C_{15}H_{20}O_4$ (264.32): C, 68.16; H, 7.63. Found: C, 68.15; H, 7.66.

**1-(1-Hydroxycyclohexyl)-2-phenoxyethan-1-one (4j)**
Yield 141 mg, 60% (Method A); Yield 140 mg, 60% (Method B). Colorless crystallizing substance.
IR (KBr): 3064, 2933, 2857, 1731, 1710, 1600, 1497, 1446, 1433, 1409, 1387, 1351, 1306, 1291, 1247, 1228, 1200, 1175, 1153, 1130, 1084, 1038, 984, 928, 883, 862, 842, 828, 751, 689, 580, 555, 533, 507, 437 $cm^{-1}$.
$^1H$ NMR (400.1 MHz, $CDCl_3$): δ = 7.28-7.24 (m, 2 H, Ph), 6.98-6.94 (m, 1 H, Ph), 6.89-6.86 (m, 2 H, Ph), 5.00 (s, 2 H, $CH_2$), 2.94 (s, 1 H, OH), 1.83-1.58 [m, 9 H, $(CH_2)_5$], 1.33-1.23 [m, 1 H, $(CH_2)_5$].
$^{13}C$ NMR (100.6 MHz, $CDCl_3$): δ = 209.3 (C=O), 157.9, 129.6, 121.7, 114.7 (Ph), 78.3 (C–OH), 69.4 ($CH_2$), 34.1, 25.1, 20.8 [$(CH_2)_5$].
MS (EI): $m/z$ (%) = 234 (29) [$M^+$], 141 (10), 112 (18), 108 (47), 107 (39), 99 (29), 97 (12), 95 (24), 94 (100), 81 (28), 79 (20), 77 (38), 70 (12), 69 (13), 65 (13), 55 (44), 51 (10), 43 (60), 42 (10), 41 (22), 39 (18).

Anal. Calcd for $C_{14}H_{18}O_3$ (234.30): C, 71.77; H, 7.74. Found: C, 71.80; H, 7.69.

**1-(1-Hydroxycyclohexyl)-2-(4-nitrophenoxy)ethan-1-one (4k)**
Yield 217 mg, 78% (Method B). Colorless crystals; mp 110-114 °C.
IR (KBr): 3114, 2934, 2856, 1716, 1590, 1512, 1450, 1415, 1340, 1274, 1228, 1182, 1111, 1061, 990, 846, 751, 688, 637, 518 $cm^{-1}$.
$^1$H NMR [400.1 MHz, $(CD_3)_2CO$]: δ = 8.19-8.15 (m, 2 H, Ph), 7.05-7.01 (m, 2 H, Ph), 5.42 (s, 2 H, $CH_2$), 4.53 (s, 1 H, OH), 1.74-1.52 [m, 9 H, $(CH_2)_5$], 1.32-1.23 [m, 1 H, $(CH_2)_5$].
$^{13}$C NMR [100.6 MHz, $(CD_3)_2CO$]: δ = 209.5 (C=O), 164.4 (C*i*), 142.3 (C*p*), 126.2, 115.5 (Ph), 78.7 (C–OH), 70.2 ($CH_2$), 34.2, 25.8, 21.3 [$(CH_2)_5$].
MS (EI): *m/z* (%) = 279 (4) [$M^+$], 167 (14), 150 (12), 149 (43), 125 (12), 123 (13), 113 (11), 109 (20), 99 (74), 98 (17), 95 (24), 94 (12), 93 (26), 85 (21), 83 (49), 82 (19), 81 (31), 80 (11), 71 (53), 70 (29), 69 (48), 65 (43), 64 (10), 57 (100), 56 (19), 55 (58), 45 (13), 44 (15), 43 (45), 41 (34), 39 (23).
Anal. Calcd for $C_{14}H_{17}NO_5$ (279.29): C, 60.21; H, 6.14; N, 5.02. Found: C, 60.33; H, 6.12; N, 5.00.

**3-Hydroxy-3,4,4-trimethyl-1-phenoxypentan-2-one (4l)**
Yield 80 mg, 34% (Method A); Yield 46 mg, 39% (Method B, conversion of **1b** was 50%). Colorless crystals; mp 91-94 °C.
IR (KBr): 2983, 2959, 2908, 2875, 1721, 1600, 1587, 1497, 1446, 1415, 1394, 1364, 1337, 1292, 1247, 1222, 1163, 1140, 1100, 1033, 1006, 975, 912, 884, 850, 756, 719, 691, 623, 557, 509, 477, 450 $cm^{-1}$.
$^1$H NMR (400.1 MHz, $CDCl_3$): δ = 7.32-7.19 (m, 2 H, Ph), 7.00-6.85 (m, 3 H, Ph), 5.07-4.94 (m, 2 H, $CH_2$), 2.36 (s, 1 H, OH), 1.37 (s, 3 H, $CH_3$), 1.01 [s, 9 H, $(CH_3)_3$].
$^{13}$C NMR (100.6 MHz, $CDCl_3$): δ = 209.5 (C=O), 158.2, 129.6, 121.6, 115.0 (Ph), 83.8 (C–OH), 71.6 ($CH_2$), 38.0 [*C*-$(CH_3)_3$], 25.3 [$(CH_3)_3$], 21.7 ($CH_3$).
MS (EI): *m/z* (%) = 236 (2) [$M^+$], 194 (17), 136 (32), 108 (57), 107 (14), 102 (14), 101 (100), 95 (11), 94 (88), 85 (13), 83 (86), 77 (42), 59 (12), 57 (34), 55 (44), 51 (15), 43 (65), 41 (42), 39 (18).
Anal. Calcd for $C_{14}H_{20}O_3$ (236.31): C, 71.16; H, 8.53. Found: C, 71.12; H, 8.55.

**3-Methyl-1,1-diphenoxybutan-2-one (5a)**
Yield 30 mg, 22 % (Method A). Pale yellow oil.
IR (film): 3066, 3042, 2975, 2932, 2874, 1815, 1731, 1595, 1492, 1469, 1383, 1367, 1329, 1289, 1240, 1203, 1172, 1103, 1031, 991, 894, 857, 834, 754, 692, 612, 506 $cm^{-1}$.
$^1$H NMR (400.1 MHz, $CDCl_3$): δ = 7.34-7.20 (m, 5 H, Ph), 7.12-6.93 (2m, 5 H, Ph), 5.85 [s, 1 H, C*H*-$(OPh)_2$], 3.34-3.24 [m, 1 H, C*H*-$(CH_3)_2$], 1.16 [d, *J* = 6.8 Hz, 6 H, $(CH_3)_2$].
$^{13}$C NMR (100.6 MHz, $CDCl_3$): δ = 207.5 (C=O) , 156.0, 129.8, 123.3, 117.3 (Ph), 100.2 [*C*H-$(OPh)_2$], 35.8 [*C*H-$(CH_3)_2$], 18.7 [$(CH_3)_2$].
MS (EI): *m/z* (%) = 200 (42) [M – ($Me_2CHCO$)]$^+$, 199 (100), 171 (11), 153 (52), 152 (17), 107 (18), 105 (10), 95 (13), 94 (16), 93 (13), 78 (13), 77 (80), 65 (16), 55 (32), 51 (35), 43 (37), 41 (19), 39 (26).
Anal. Calcd for $C_{17}H_{18}O_3$ (270.33): C, 75.53; H, 6.71. Found: C, 75.55; H, 6.70.

**3-Methyl-1,1-bis(naphthalen-1-yloxy)butan-2-one (5b)**
Purity 88%, yield 10 mg, 5% (Method A). Pale yellow oil.
IR (film): 3056, 2973, 2928, 2873, 2852, 1820, 1730, 1630, 1596, 1579, 1507, 1463, 1396, 1317, 1263, 1234, 1175, 1156, 1122, 1087, 1059, 1018, 985, 894, 792, 771, 740, 569 $cm^{-1}$.
$^1$H NMR (400.1 MHz, $CDCl_3$): δ = 8.26-8.24 (m, 2 H, Naphthyl), 7.81-7.79 (m, 2 H, Naphthyl), 7.53-7.47 (m, 6 H, Naphthyl), 7.25-7.23 (m, 2 H, Naphthyl), 6.94-6.92 (m, 2 H, Naphthyl), 6.26 [s, 1 H, C*H*-$(ONaphthyl)_2$], 3.55-3.44 [m, 1 H, C*H*-$(CH_3)_2$], 1.26 [d, *J* = 6.9 Hz, 6 H, $(CH_3)_2$].

$^{13}C$ NMR (100.6 MHz, $CDCl_3$): δ = 207.4 (C=O) , 151.8, 134.8, 127.7, 126.8, 126.0, 125.9, 125.7, 122.9, 122.0, 109.1 (Naphthyl), 100.7 [*C*H-(ONaphthyl)$_2$], 35.8 [*C*H-($CH_3$)$_2$], 18.9 [($CH_3$)$_2$].
MS (EI): *m/z* (%) = 370 (2) [$M^+$], 369 (10), 300 (21), 299 (81), 186 (12), 155 (13), 129 (16), 127 (33), 126 (17), 125 (17), 115 (100), 69 (13), 51 (10), 43 (95), 41 (12).
Anal. Calcd for $C_{25}H_{22}O_3$ (370.45): C, 81.06; H, 5.99. Found: C, 81.08; H, 5.96.

**3-Methyl-1,1-bis(naphthalen-2-yloxy)butan-2-one (5c)**
Purity 87%, yield 25 mg, 12% (Method A). Pale yellow oil.
IR (film): 3058, 3029, 2973, 2931, 2874, 2853, 1821, 1730, 1631, 1599, 1510, 1466, 1444, 1386, 1359, 1249, 1210, 1164, 1120, 1099, 1043, 1006, 962, 914, 845, 811, 748, 623, 474 $cm^{-1}$.
$^1H$ NMR (400.1 MHz, $CDCl_3$): δ = 7.78-7.76 (m, 4 H, Naphthyl), 7.68-7.66 (m, 2 H, Naphthyl), 7.44-7.32 (m, 6 H, Naphthyl), 7.25-7.22 (m, 2 H, Naphthyl), 6.13 [s, 1 H, C*H*-(ONaphthyl)$_2$], 3.42-3.35 [m, 1 H, C*H*-($CH_3$)$_2$], 1.21 [d, *J* = 6.8 Hz, 6 H, ($CH_3$)$_2$].
$^{13}C$ NMR (100.6 MHz, $CDCl_3$): δ = 207.5 (C=O) , 153.7, 134.2, 130.2, 130.0, 127.8, 127.3, 126.7, 124.8, 119.1, 111.7 (Naphthyl), 100.3 [*C*H-(ONaphthyl)$_2$], 35.9 [*C*H-($CH_3$)$_2$], 18.8 [($CH_3$)$_2$].
MS (EI): *m/z* (%) = 370 (9) [$M^+$], 300 (29), 299 (100), 157 (32), 144 (16), 143 (29), 128 (14), 127 (73), 126 (17), 115 (39), 71 (12), 55 (11), 43 (27).
Anal. Calcd for $C_{25}H_{22}O_3$ (370.45): C, 81.06; H, 5.99. Found: C, 81.03; H, 5.98.

**3-Methyl-1,1-bis(*p*-tolyloxy)butan-2-one (5d)**
Yield 24 mg, 16% (Method A). Pale yellow oil.
IR (film): 3032, 2973, 2925, 2873, 1730, 1611, 1589, 1508, 1466, 1382, 1364, 1320, 1285, 1241, 1203, 1175, 1144, 1105, 1043, 1006, 933, 896, 864, 817, 753, 720, 660, 510 $cm^{-1}$.
$^1H$ NMR (400.1 MHz, $CDCl_3$): δ = 7.04 (d, *J* = 8.5 Hz, 4 H, Ph), 6.85 (d, *J* = 8.5 Hz, 4 H, Ph), 5.74 [s, 1 H, C*H*-(OPh)$_2$], 3.32-3.23 [m, 1 H, C*H*-($CH_3$)$_2$], 2.26 [s, 6 H, Ph-(C*H*$_3$)$_2$], 1.15 [d, *J* = 6.8 Hz, 6 H, CH-(C*H*$_3$)$_2$].
$^{13}C$ NMR (100.6 MHz, $CDCl_3$): δ = 207.7 (C=O) , 153.9, 130.2, 129.8, 117.2 (Ph), 100.8 [*C*H-(OPh)$_2$], 35.7 [*C*H-($CH_3$)$_2$], 20.7 [Ph-(*C*H$_3$)$_2$], 18.7 [CH-(*C*H$_3$)$_2$].
MS (EI): *m/z* (%) = 298 (2) [$M^+$], 228 (40), 227 (100), 199 (12), 166 (25), 121 (28), 119 (12), 109 (18), 108 (14), 107 (55), 91 (67), 79 (14), 77 (18), 65 (50), 55 (28), 43 (23), 41 (13), 39 (14).
Anal. Calcd for $C_{19}H_{22}O_3$ (298.38): C, 76.48; H, 7.43. Found: C, 76.47; H, 7.40.

**1,1-Bis(4-methoxyphenoxy)-3-methylbutan-2-one (5e)**
Yield 32 mg, 19 % (Method A). Pale yellow oil.
IR (film): 3047, 2997, 2972, 2934, 2875, 2836, 1821, 1729, 1679, 1639, 1609, 1593, 1505, 1465, 1443, 1384, 1295, 1233, 1197, 1126, 1103, 1036, 1005, 946, 896, 829, 799, 768, 718, 661, 643, 523 $cm^{-1}$.
$^1H$ NMR (400.1 MHz, $CDCl_3$): δ = 6.92-6.89 (m, 4 H, Ph), 6.80-6.77 (2m, 4 H, Ph), 5.63 [s, 1 H, C*H*-(OPh)$_2$], 3.74 [s, 6 H, ($OCH_3$)$_2$], 3.29-3.23 [m, 1 H, C*H*-($CH_3$)$_2$], 1.15 [d, *J* = 6.8 Hz, 6 H, ($CH_3$)$_2$].
$^{13}C$ NMR (100.6 MHz, $CDCl_3$): δ = 207.8 (C=O) , 155.7, 150.0, 119.0, 114.8 (Ph), 102.2 [*C*H-(OPh)$_2$], 55.7 [($OCH_3$)$_2$], 35.8 [*C*H-($CH_3$)$_2$], 18.7 [($CH_3$)$_2$].
Anal. Calcd for $C_{19}H_{22}O_5$ (330.38): C, 69.07; H, 6.71. Found: C, 69.07; H, 6.73.

**1,1-Bis(4-bromophenoxy)-3-methylbutan-2-one (5f)**
Yield 51 mg, 24 % (Method A). Pale yellow oil.
IR (film): 3095, 3070, 2974, 2931, 2874, 1733, 1642, 1584, 1485, 1403, 1383, 1366, 1321, 1278, 1241, 1203, 1172, 1102, 1068, 1047, 1006, 938, 897, 824, 684, 671, 504 $cm^{-1}$.

$^{1}$H NMR (400.1 MHz, $CDCl_3$): δ = 7.39-7.36 (m, 4 H, Ph), 6.87-6.84 (2m, 4 H, Ph), 5.76 [s, 1 H, C*H*-$(OPh)_2$], 3.26-3.19 [m, 1 H, C*H*-$(CH_3)_2$], 1.15 [d, *J* = 6.8 Hz, 6 H, $(CH_3)_2$].
$^{13}$C NMR (100.6 MHz, $CDCl_3$): δ = 206.6 (C=O) , 154.8, 132.8, 121.4, 119.1, 116.1 (Ph), 100.0 [*C*H-$(OPh)_2$], 36.0 [*C*H-$(CH_3)_2$], 18.6 [$(CH_3)_2$].
Anal. Calcd for $C_{17}H_{16}Br_2O_3$ (428.12): C, 47.69; H, 3.77; Br, 37.33. Found: C, 47.71; H, 3.74; Br, 37.33.

**1,1-Bis(4-allyl-2-methoxyphenoxy)-3-methylbutan-2-one (5g)**
Yield 45 mg, 22 % (Method A). Pale yellow oil.
IR (film): 3079, 3060, 3014, 2975, 2937, 2915, 2876, 2839, 1818, 1729, 1639, 1593, 1509, 1465, 1419, 1384, 1368, 1311, 1268, 1216, 1207, 1152, 1125, 1097, 1036, 995, 917, 851, 812, 756, 668 $cm^{-1}$.
$^{1}$H NMR (400.1 MHz, $CDCl_3$): δ = 6.90-6.88 (m, 2 H, Ph), 6.67-6.65 (m, 2 H, Ph), 6.62-6.59 (m, 2 H, Ph), 5.95-5.85 [m, 2 H, $(=CH)_2$], 5.69 [s, 1 H, C*H*-$(OPh)_2$], 5.07-5.00 [m, 4 H, $(=CH_2)_2$], 3.72 [s, 6 H, $(OCH_3)_2$], 3.44-3.37 [m, 1 H, C*H*-$(CH_3)_2$], 3.30-3.28 [m, 4 H, $(CH_2)_2$], 1.20 [d, *J* = 6.8 Hz, 6 H, $(CH_3)_2$].
$^{13}$C NMR (100.6 MHz, $CDCl_3$): δ = 207.3 (C=O) , 150.7, 143.7 (Ph), 137.4 [$(CH=CH_2)_2$], 120.7, 120.6 (Ph), 115.9 [$(=CH_2)_2$], 112.9 (Ph), 103.2 [*C*H-$(OPh)_2$], 55.8 [$(OCH_3)_2$], 39.9 [$(CH_2)_2$], 35.4 [*C*H-$(CH_3)_2$], 18.7 [$(CH_3)_2$].
MS (EI): *m/z* (%) = 339 (100) [M – $(Me_2CHCO)]^+$, 163 (20), 162 (17), 161 (15), 131 (11), 115 (27), 105 (11), 104 (19), 103 (22), 91 (31), 78 (13), 77 (17), 55 (23), 43 (34), 41 (29), 39 (10).
Anal. Calcd for $C_{25}H_{30}O_5$ (410.51): C, 73.15; H, 7.37. Found: C, 73.17; H, 7.34.

**(Z)-5-(Bromomethylene)-4-(*tert*-butyl)-4-methyl-1,3-dioxolan-2-one (6b)**
Yield 13 mg, 5% (Method A). Pale yellow oil.
IR (film): 3105, 2973, 2919, 2878, 1831, 1739, 1678, 1595, 1490, 1400, 1381, 1371, 1303, 1263, 1228, 1202, 1132, 1105, 1052, 1021, 995, 937, 890, 758, 691 $cm^{-1}$.
$^{1}$H NMR (400.1 MHz, $CDCl_3$): δ = 5.39 (s, 1 H, CH), 1.57 (s, 3 H, $CH_3$), 1.02 [s, 9 H, $(CH_3)_3$].
$^{13}$C NMR (100.6 MHz, $CDCl_3$): δ = 153.1 (*C*=CH), 150.3 (C=O), 93.1 (*C*–$CH_3$), 79.7 (C=*C*H), 38.4 [*C*–$(CH_3)_3$], 24.2 [$(CH_3)_3$], 21.4 ($CH_3$).
MS (EI): *m/z* (%) = 193 (2) [M-$(CH_3)_3]^+$, 125 (11), 85 (10), 84 (100), 69 (98), 67 (15), 57 (88), 56 (12), 55 (15), 53 (11), 43 (44), 41 (75), 39 (27).
Anal. Calcd for $C_{19}H_{13}BrO_3$ (249.10): C, 43.40; H, 5.26; Br, 32.08. Found: C, 43.37; H, 5.29; Br, 32.06.

**(*Z*)-4,4-Dimethyl-5-(phenoxymethylene)-1,3-dioxolan-2-one (7)**
Yield 11 mg, 5% (Method A). White crystals; mp 112-114 °C.
IR (KBr): 3048, 2983, 2937, 2870, 1802, 1737, 1679, 1593, 1492, 1363, 1228, 1153, 1127, 1069, 1025, 840, 758, 687, 614, 508 $cm^{-1}$.
$^{1}$H NMR (400.1 MHz, $CDCl_3$): δ = 7.37-7.29 (m, 2 H, Ph), 7.08 (t, *J* = 7.4 Hz, 1 H, Ph), 7.03-6.97 (m, 2 H, Ph), 6.04 (s, 1 H, CH), 1.66 [s, 6 H, $(CH_3)_2$].
$^{13}$C NMR (100.6 MHz, $CDCl_3$): δ = 157.0 ($C_{ipso}$), 150.8 (C=O), 140.6 (*C*=CH), 129.9, 123.5, 116.1 (Ph), 119.5 (C=*C*H), 83.3 [*C*$(CH_3)_2$], 28.2 [$(CH_3)_2$].
MS (EI): *m/z* (%) = 220 (2) [$M^+$], 129 (14), 97 (18), 94 (22), 87 (10), 85 (19), 84 (21), 83 (32), 82 (16), 81 (33), 77 (14), 73 (62), 71 (34), 70 (22), 69 (84), 68 (20), 67 (24), 61 (19), 60 (49), 57 (63), 56 (22), 55 (78), 51 (12), 45 (21), 44 (19), 43 (100), 42 (22), 41 (94), 39 (24).
Anal. Calcd for $C_{12}H_{12}O_4$ (220.22): C, 65.45; H, 5.49. Found: C, 65.41; H, 5.52.

Analyzing data for **6a** [4] and **8a**,**b** [5] were published in literature.

**References**


1. Nazarov, I. V.; Shvekhgeimer, G. A. *Zh. Obshch. Khim.* **1959**, *29*, 457.
2. Hofmeister, H.; Annen, K.; Laurent, H.; Wiechert, R. *Angew. Chem.* **1984**, *96*, 720-722. doi: 10.1002/ange.19840960932.
3. Shi, D.; Liu, Z.; Zhang, Z.; Shi, W.; Chen, H. *ChemCatChem*. **2015**, *7*, 1424-1426. doi: 10.1002/cctc.201500243.
4. Bogolyubov, A. A.; Chernysheva, N. B.; Semenov. V. V. *ChemHetCmpd*. **2004**, *40*, 1124-1130. doi: 10.1023/B:COHC.0000048283.51485.4e.
5. Chen, C.; You, M.; Chen, H. *Synth. Commun*. **2016**, *46*, 73-78. doi: 10.1080/00397911.2015.1121279.


## 8. Rearrangement of the synthesized phenoxyhydroxyketones 4a,f

The mixture of 3-hydroxy-3-methyl-1-phenoxybutan-2-one (**4a**, 97 mg, 0.5 mmol) or 3-hydroxy-3-methyl-1-(p-tolyloxy)butan-2-one (**4f**, 104 mg, 0.5 mmol) and neutral aluminum oxide (900 mg, 8.5 mmol) was stirred at ~150 °C for 3 h. The resulting products were washed off aluminum oxide with acetone to give 3-hydroxy-3-methyl-4-phenoxybutan-2-one (**9a**) (58 mg, 60%) or 3-hydroxy-3-methyl-4-(p-tolyloxy)butan-2-one (**9b**) (57 mg, 55%).

## 9. Characterization data for 9a, 9b

**3-Hydroxy-3-methyl-4-phenoxybutan-2-one (9a)**
Yield 58 mg, 60%. Yellow oil.
IR (KBr): 3063, 3040, 2978, 2928, 2873, 1716, 1599, 1495, 1459, 1417, 1382, 1355, 1292, 1243, 1189, 1172, 1153, 1133, 1095, 1078, 1047, 994, 965, 887, 817, 755, 692, 640, 611, 596, 569, 550, 510 $cm^{-1}$.
$^{1}H$ NMR (400.1 MHz, $CDCl_3$): δ = 7.38-7.23 (m, 2 H, Ph), 6.97-6.93 (m, 1 H, Ph), 6.87-6.84 (m, 2 H, Ph), 4.17-4.14 (m, 1 H, $CH_2$), 3.99-3.96 (m, 2 H, OH, $CH_2$), 2.29 (s, 3 H, $CH_3CO$), 1.41 (s, 3 H, $CH_3$COH).
$^{13}C$ NMR (100.6 MHz, $CDCl_3$): δ = 210.4 (C=O), 158.2, 129.6, 121.5, 114.6 (Ph), 78.4 (C–OH), 73.0 ($CH_2$), 24.7 ($CH_3$CO), 21.8 ($CH_3$COH).
Anal. Calcd for $C_{11}H_{14}O_3$ (194.23): C, 68.02; H, 7.27. Found: C, 68.05; H, 7.25.

**3-Hydroxy-3-methyl-4-(*p*-tolyloxy)butan-2-one (9b)**
Yield 57 mg, 55%. Yellow oil.
IR (KBr): 3061, 3031, 2979, 2926, 2902, 2870, 1716, 1614, 1587, 1512, 1460, 1419, 1381, 1355, 1291, 1243, 1176, 1134, 1110, 1048, 965, 943, 900, 844, 817, 757, 696, 629, 594, 549, 512 $cm^{-1}$.
$^{1}H$ NMR (400.1 MHz, $CDCl_3$): δ = 7.07-7.03 (m, 2 H, Ph), 6.79-6.74 (m, 2 H, Ph), 4.14-4.11 (m, 1 H, $CH_2$), 3.99-3.93 (m, 2 H, OH, $CH_2$), 2.28 (s, 3 H, $CH_3CO$), 2.25 (s, 3 H, $CH_3$Ph), 1.40 (s, 3 H, $CH_3$COH).
$^{13}C$ NMR (100.6 MHz, $CDCl_3$): δ = 210.5 (C=O), 156.2, 130.8, 130.0, 114.6 (Ph), 78.4 (C–OH), 73.3 ($CH_2$), 24.7 ($CH_3$CO), 21.8 ($CH_3$Ph), 20.5 ($CH_3$COH).
Anal. Calcd for $C_{12}H_{16}O_3$ (208.26): C, 69.21; H, 7.74. Found: C, 69.22; H, 7.75.

## 10. $^{1}H$ and $^{13}C$ spectra of the synthesized products

### 4-Bromo-2-methyl-3-phenoxybut-3-en-2-ol (3a)

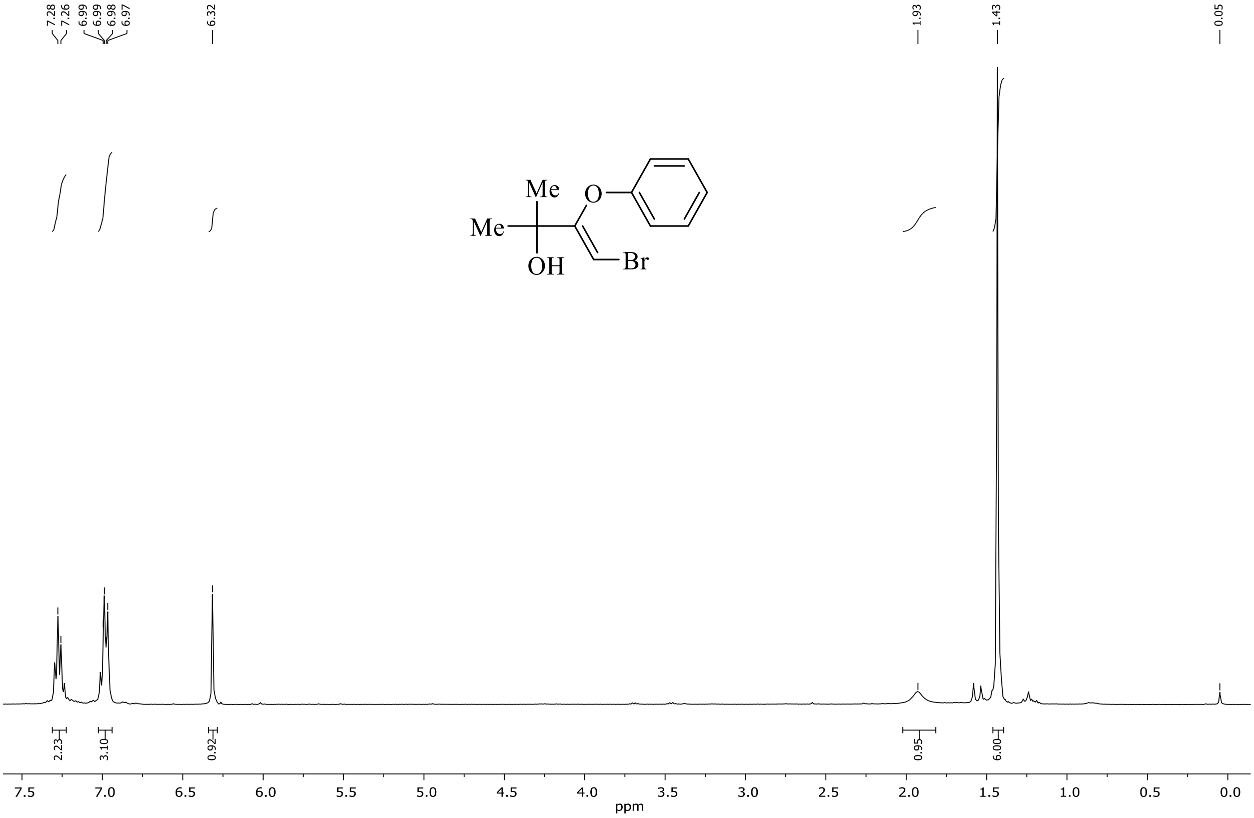


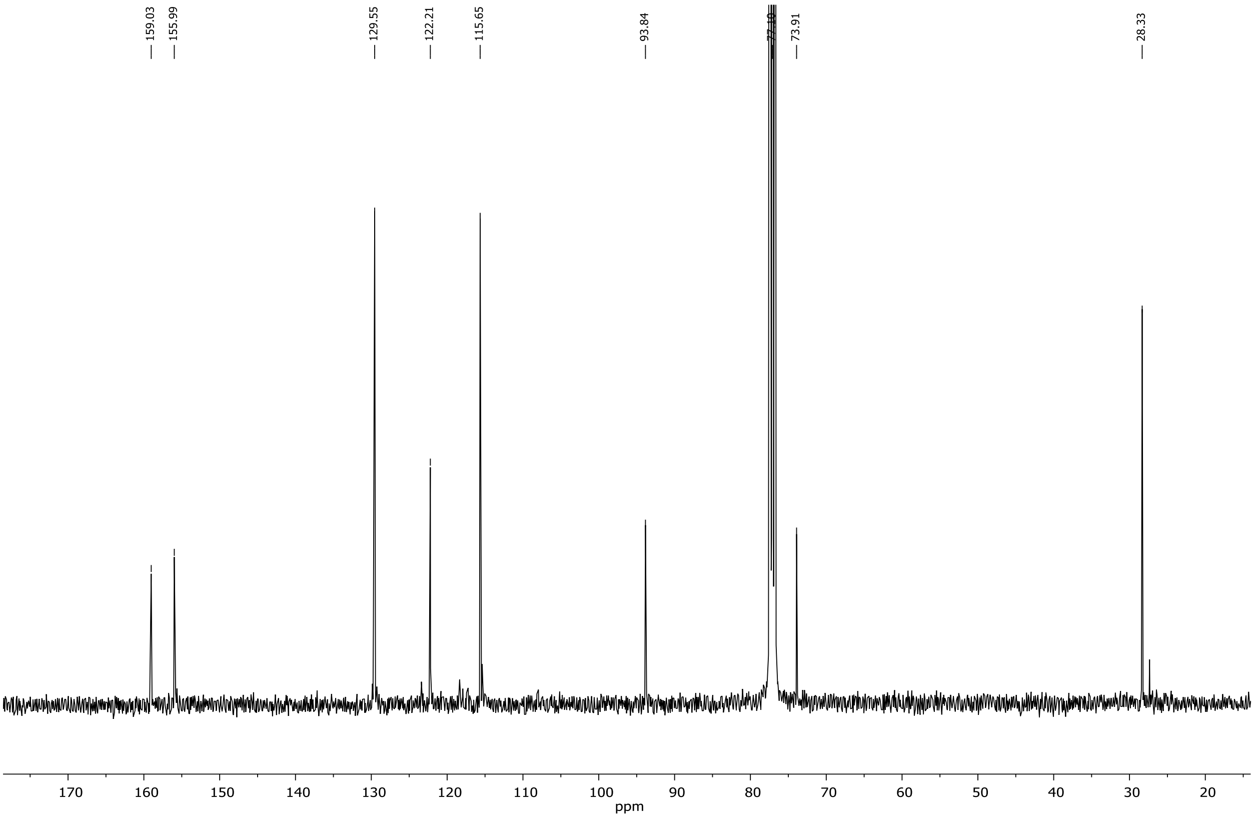

## 3-Hydroxy-3-methyl-1-phenoxybutan-2-one (4a)

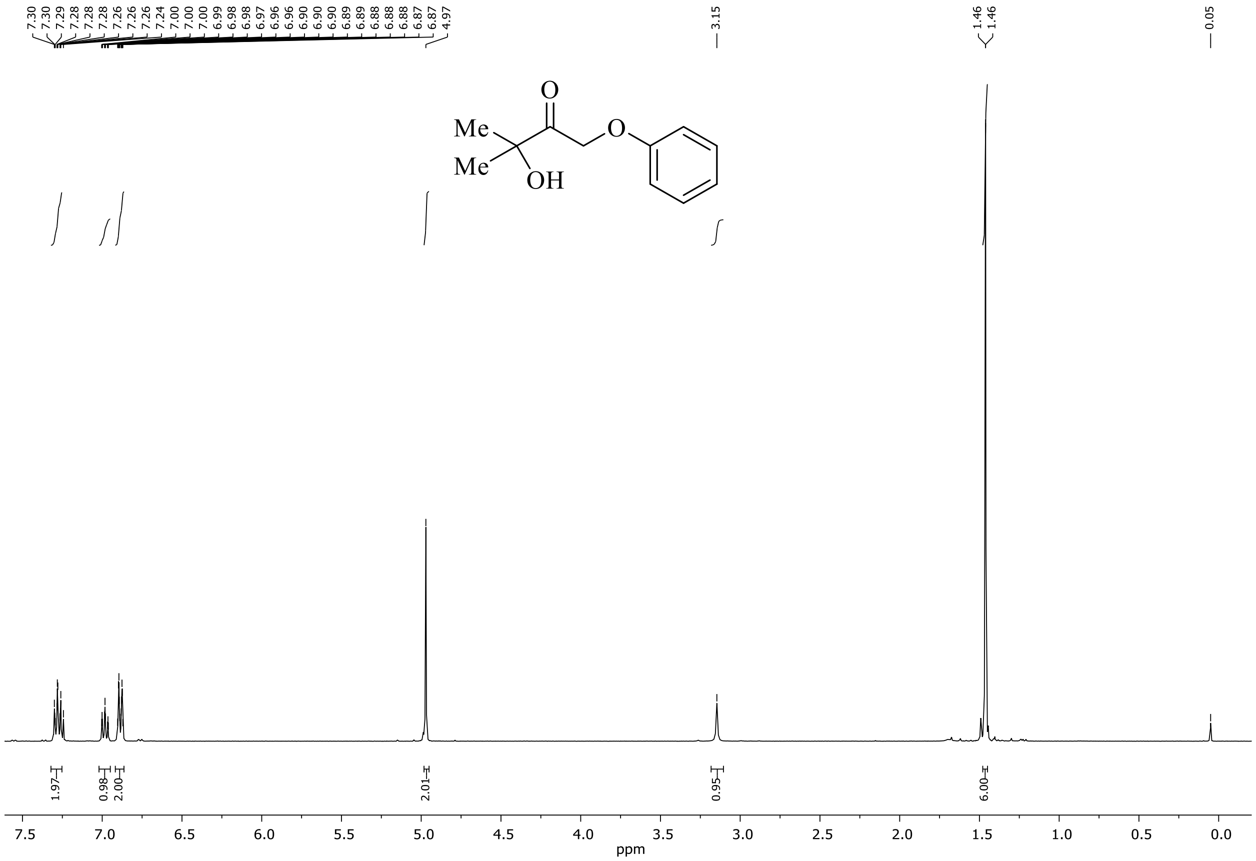


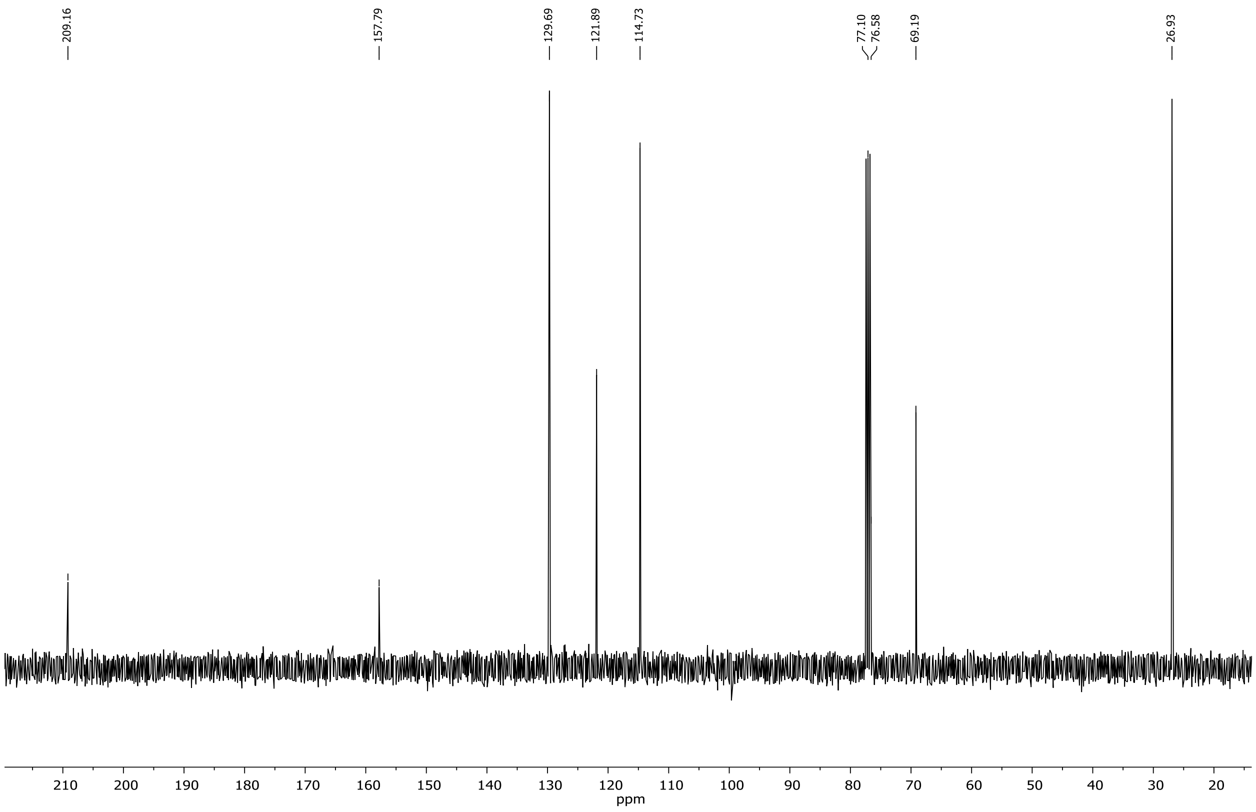

# 3-Hydroxy-3-methyl-1-(naphthalen-1-yloxy)butan-2-one (4b)

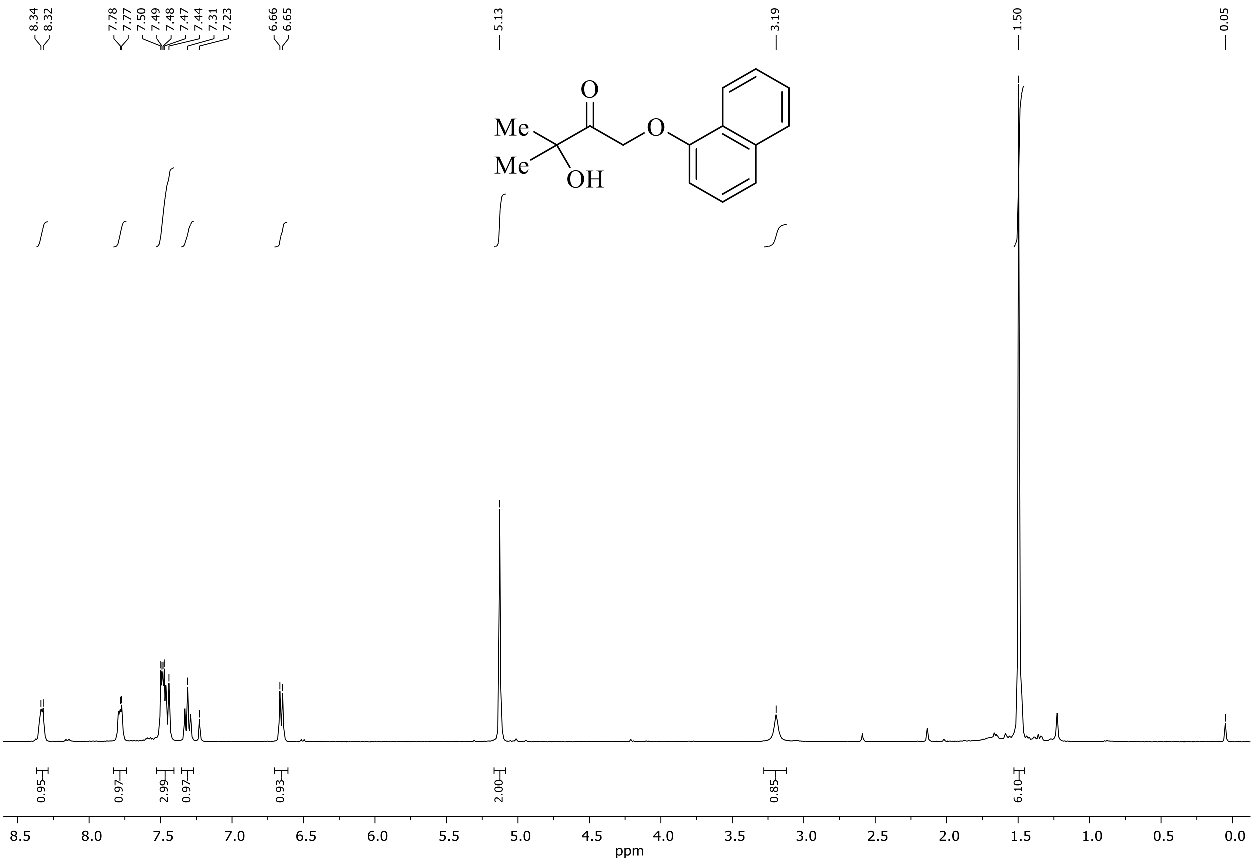


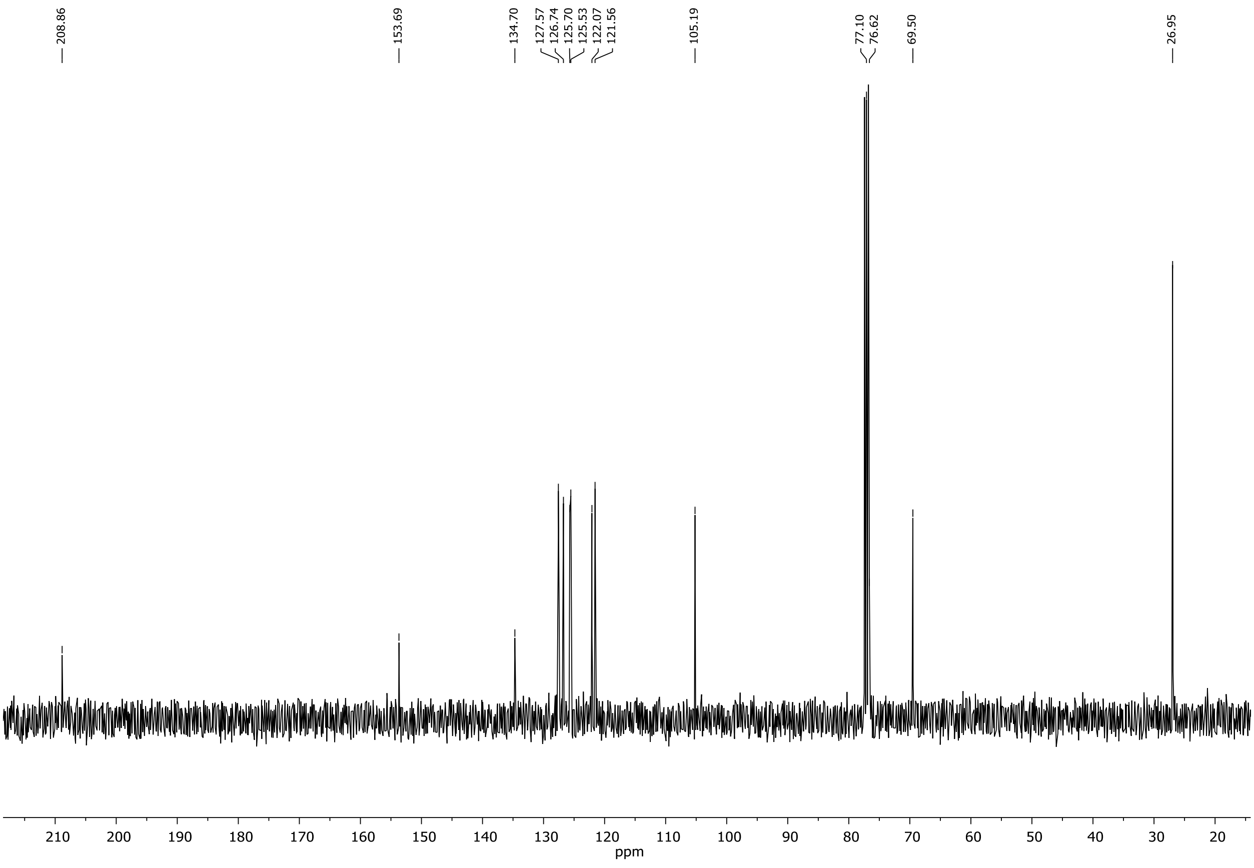

## 3-Hydroxy-3-methyl-1-(naphthalen-2-yloxy)butan-2-one (4c)

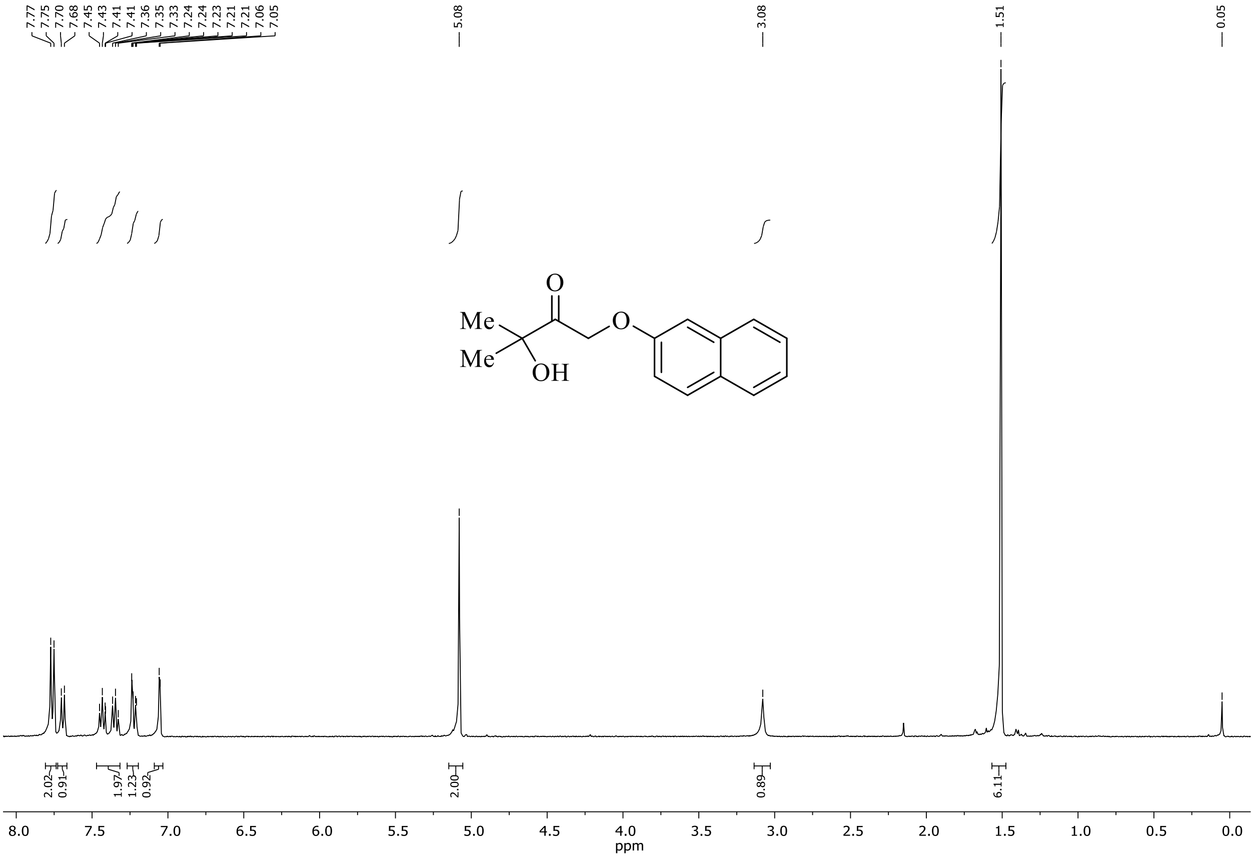


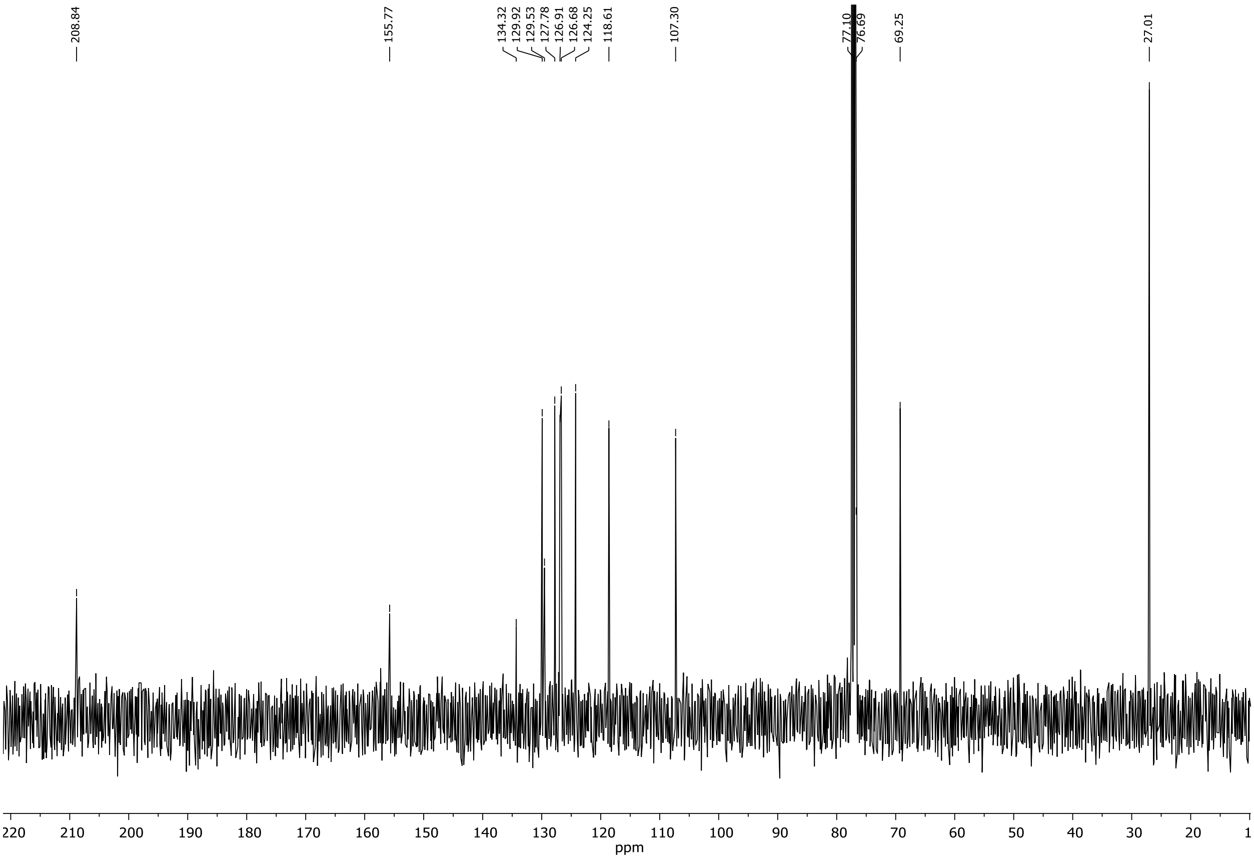

**3-Hydroxy-3-methyl-1-(4-nitrophenoxy)butan-2-one (4d)**

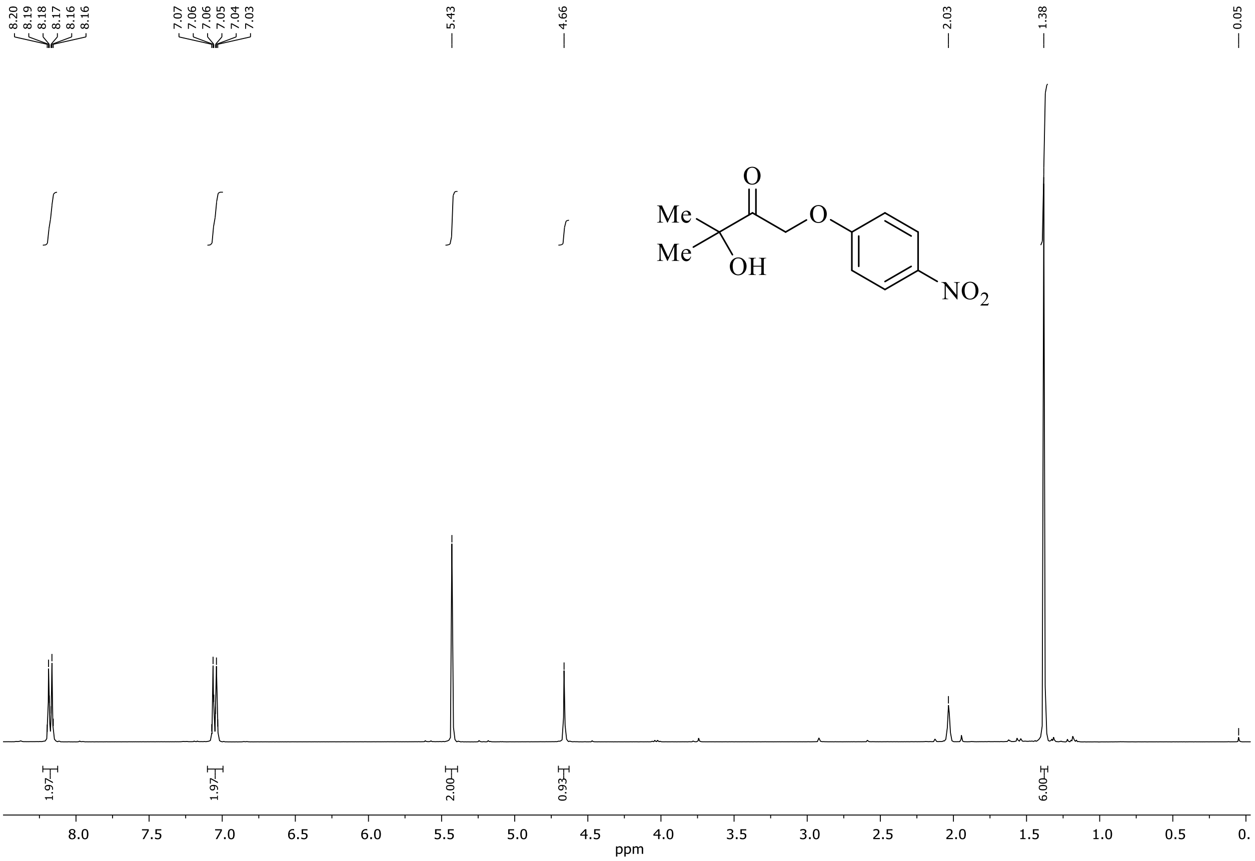


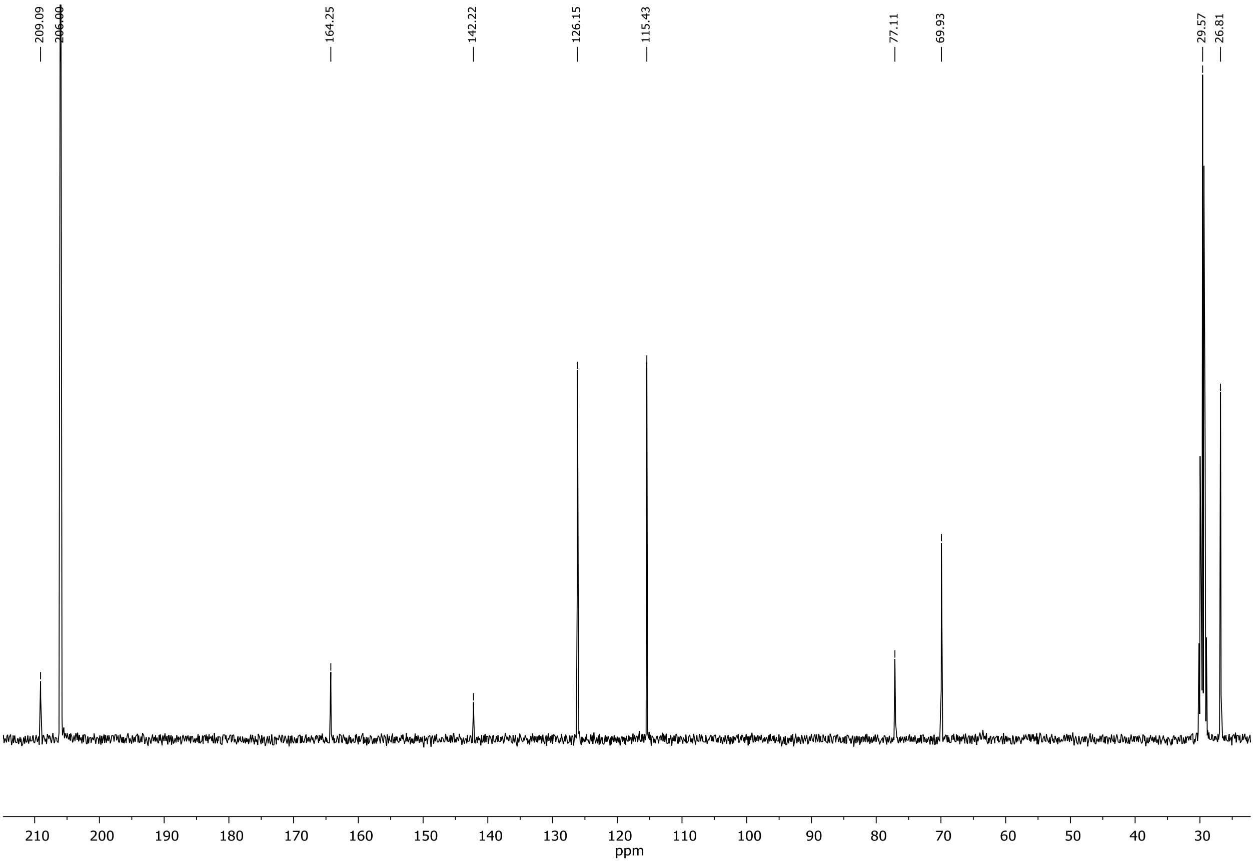

## 3-Hydroxy-3-methyl-1-(2-nitrophenoxy)butan-2-one (4e)

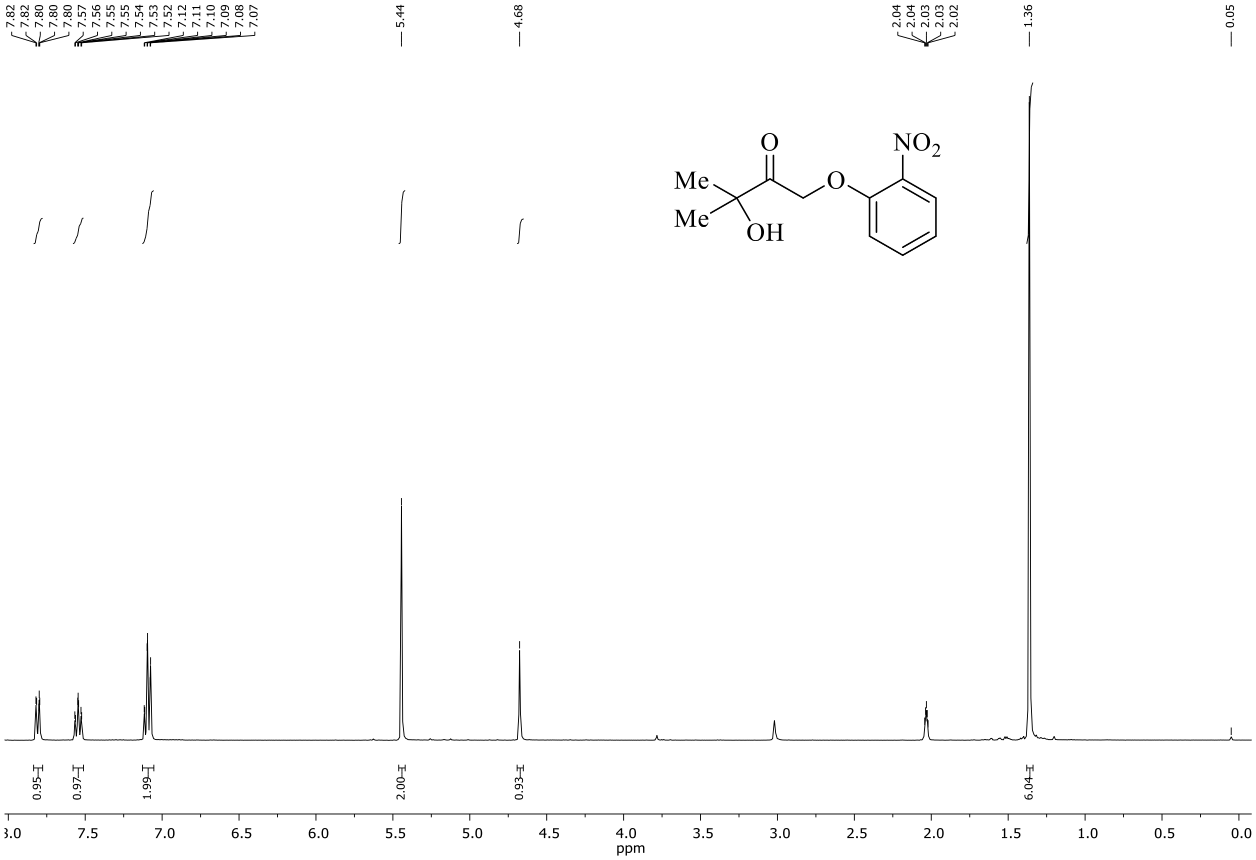


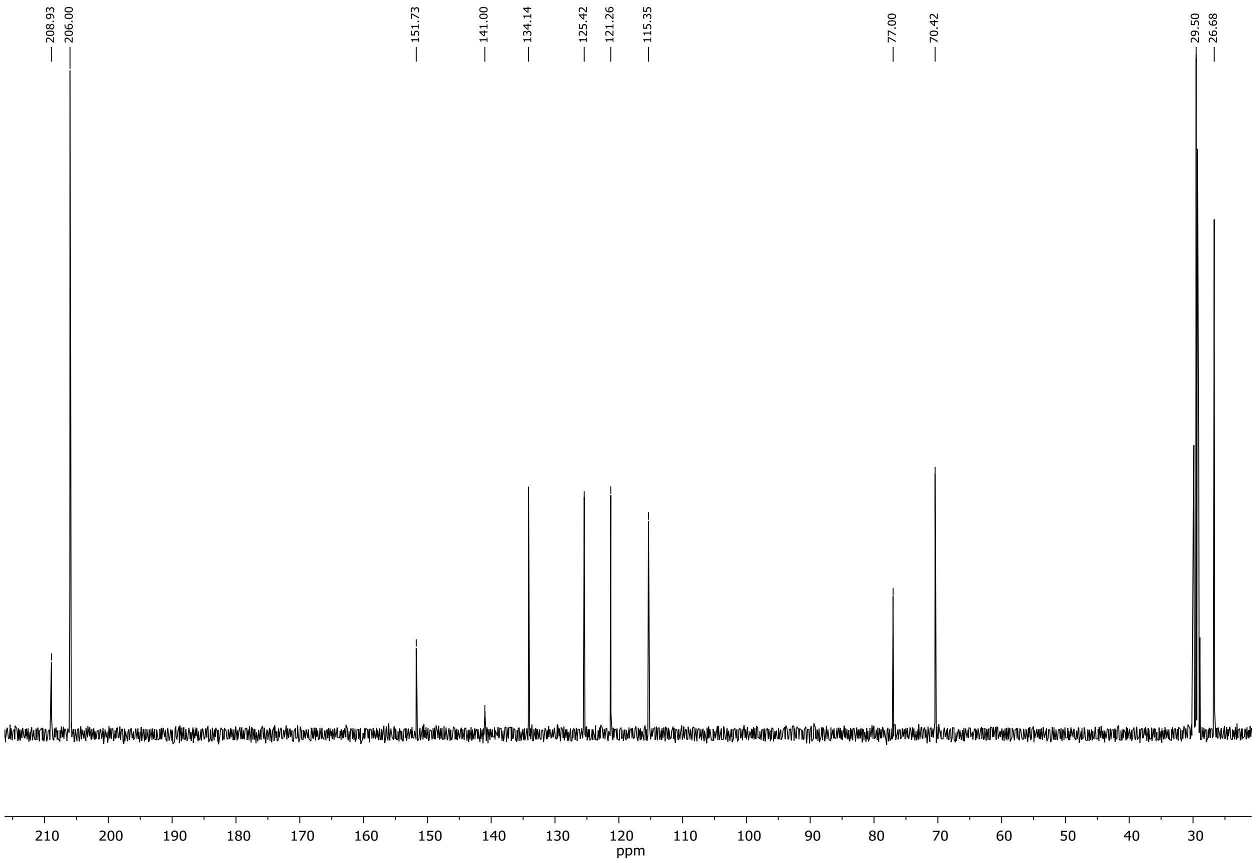

### 3-Hydroxy-3-methyl-1-(*p*-tolyloxy)butan-2-one (4f)

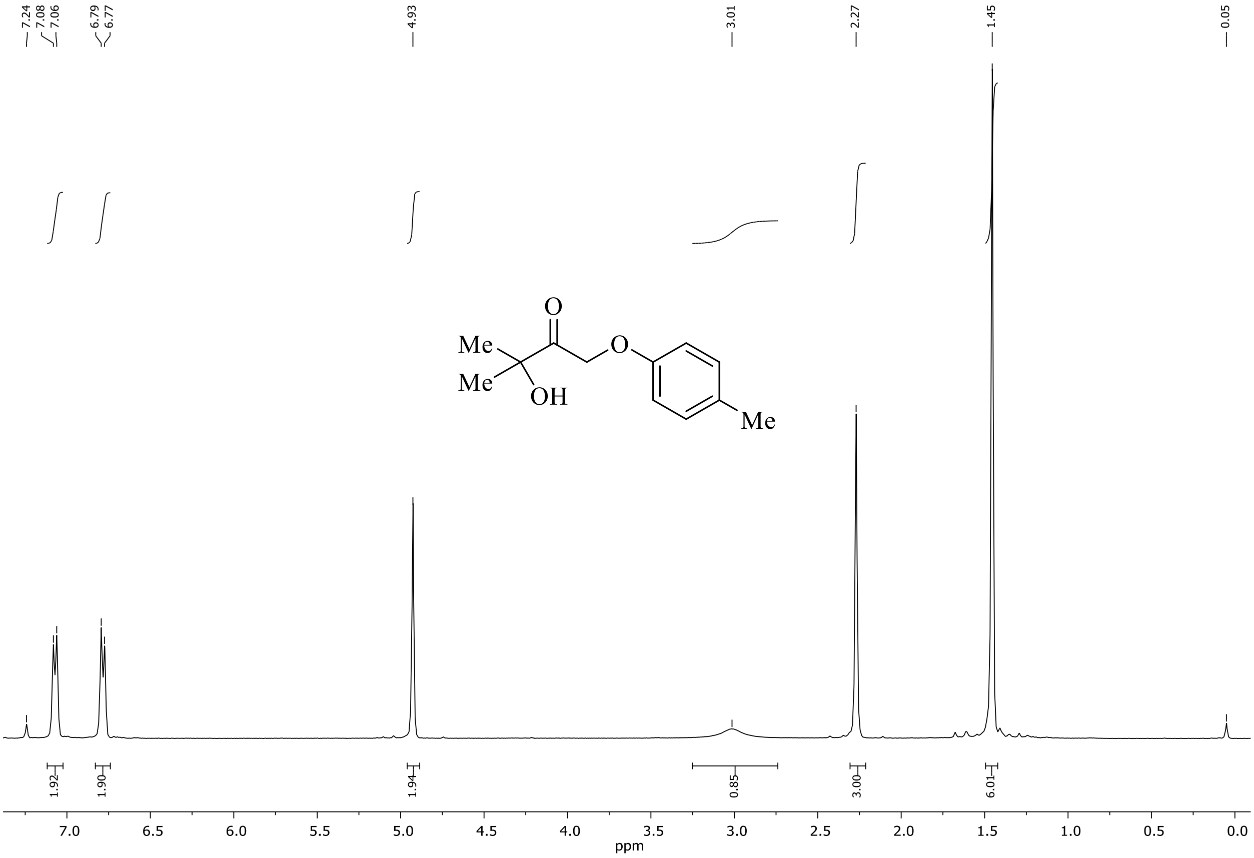


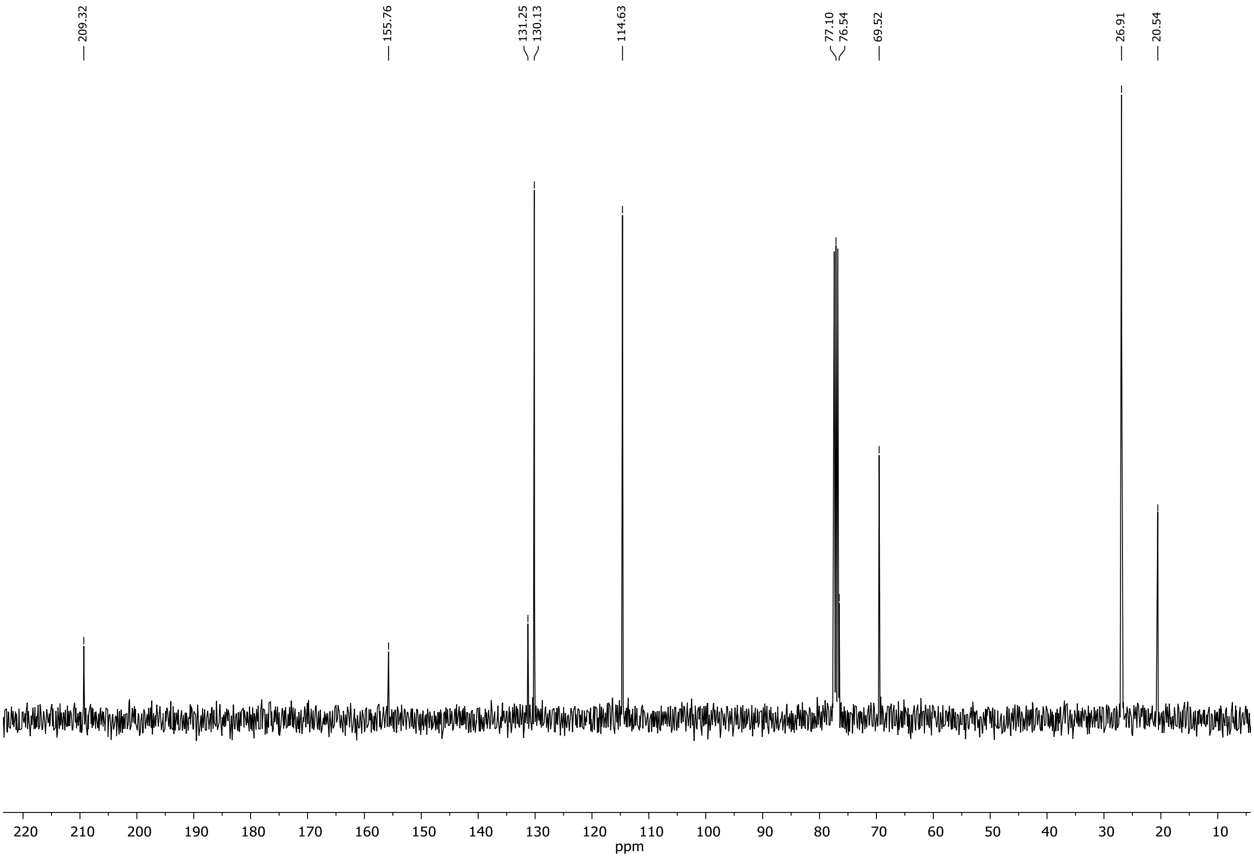

# 3-Hydroxy-1-(4-methoxyphenoxy)-3-methylbutan-2-one (4g)

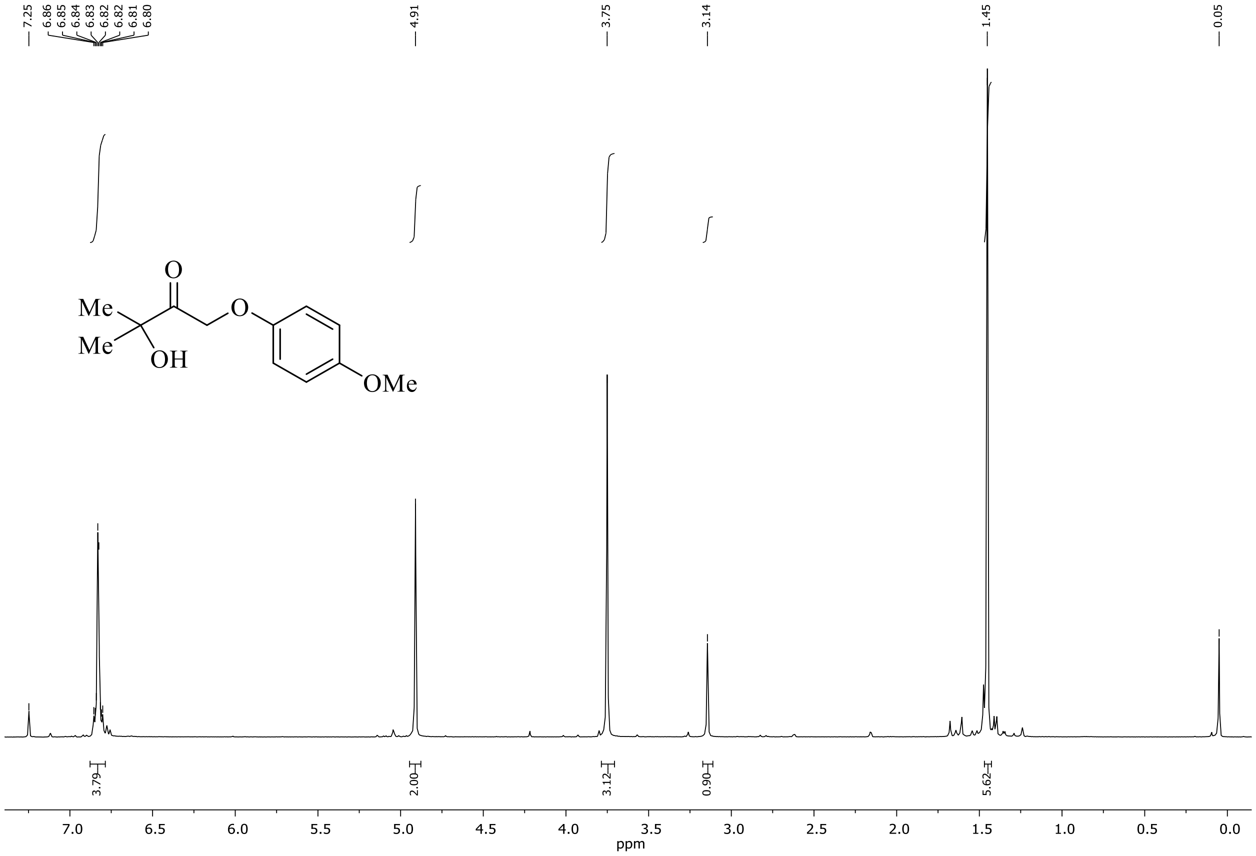


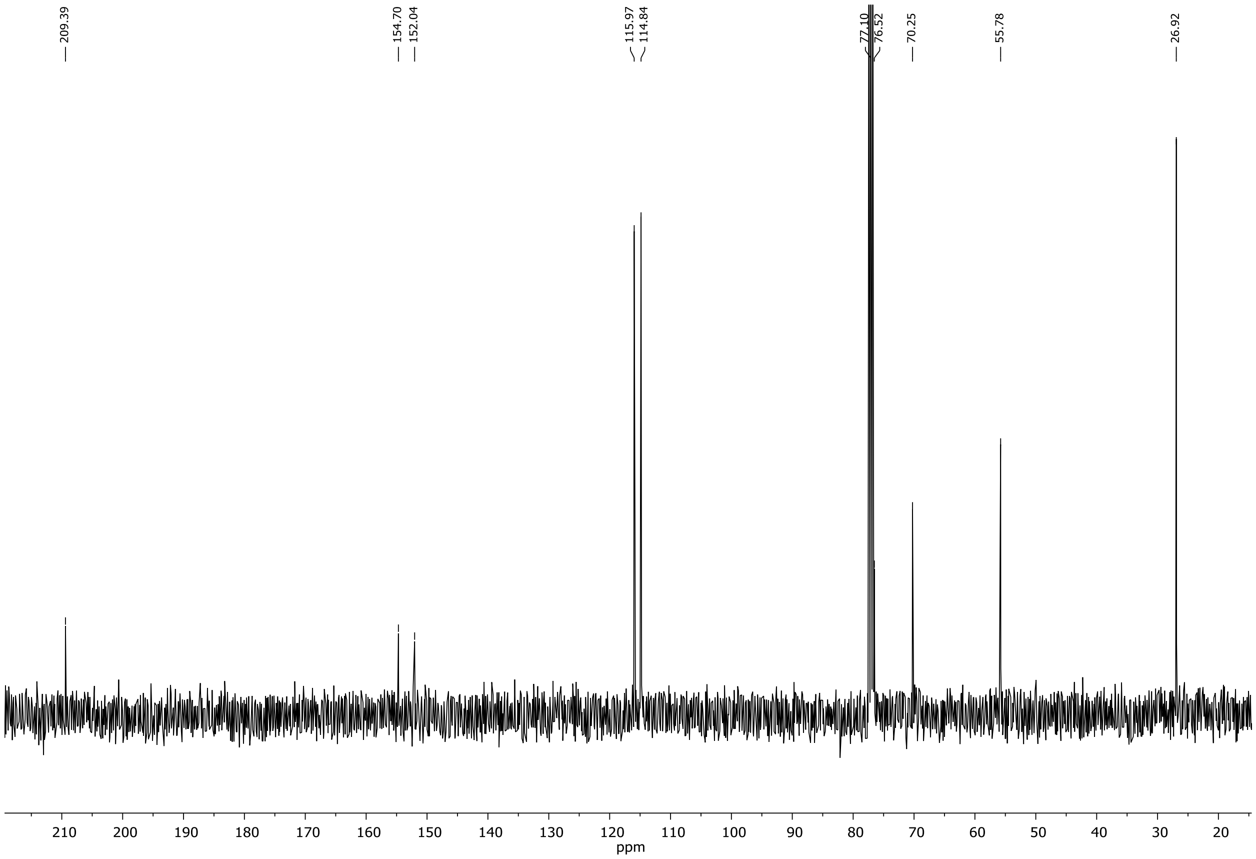

# 1-(4-Bromophenoxy)-3-hydroxy-3-methylbutan-2-one (4h)

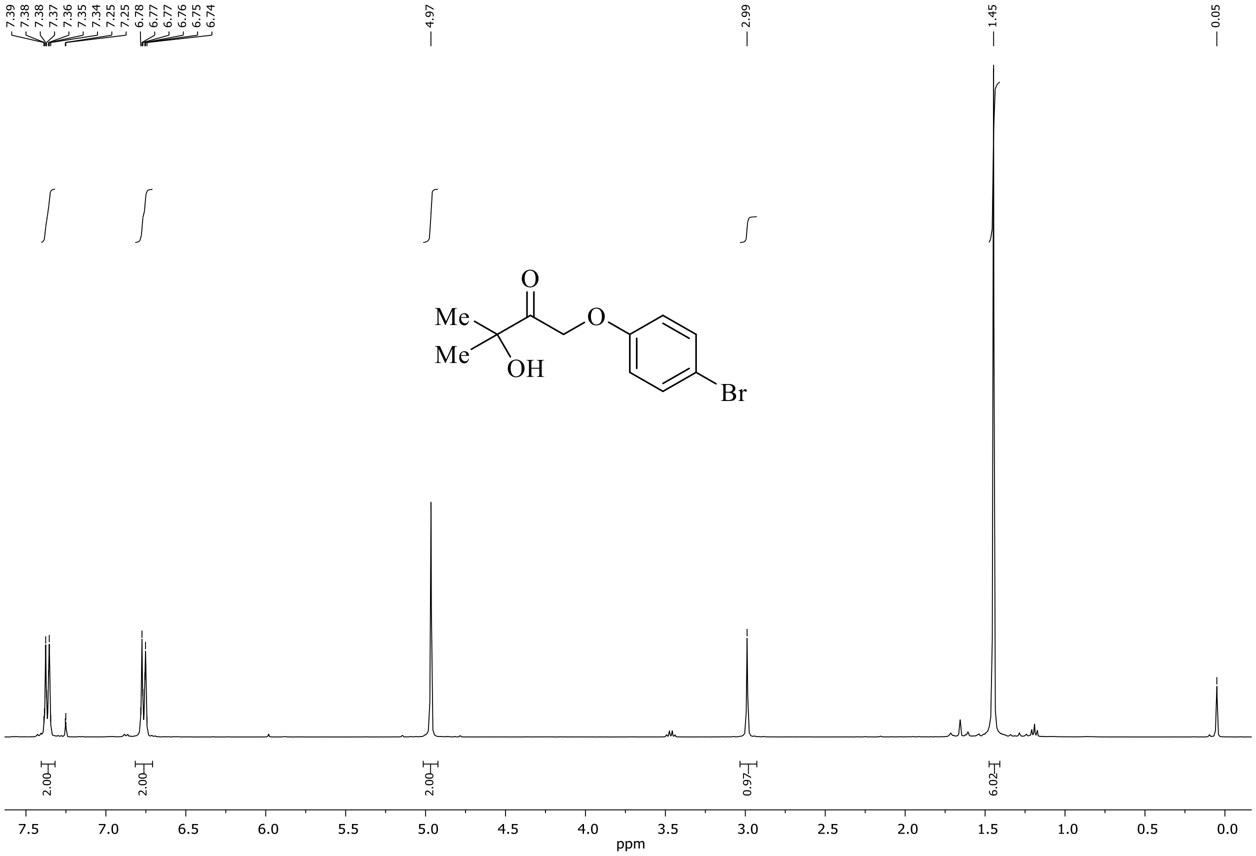


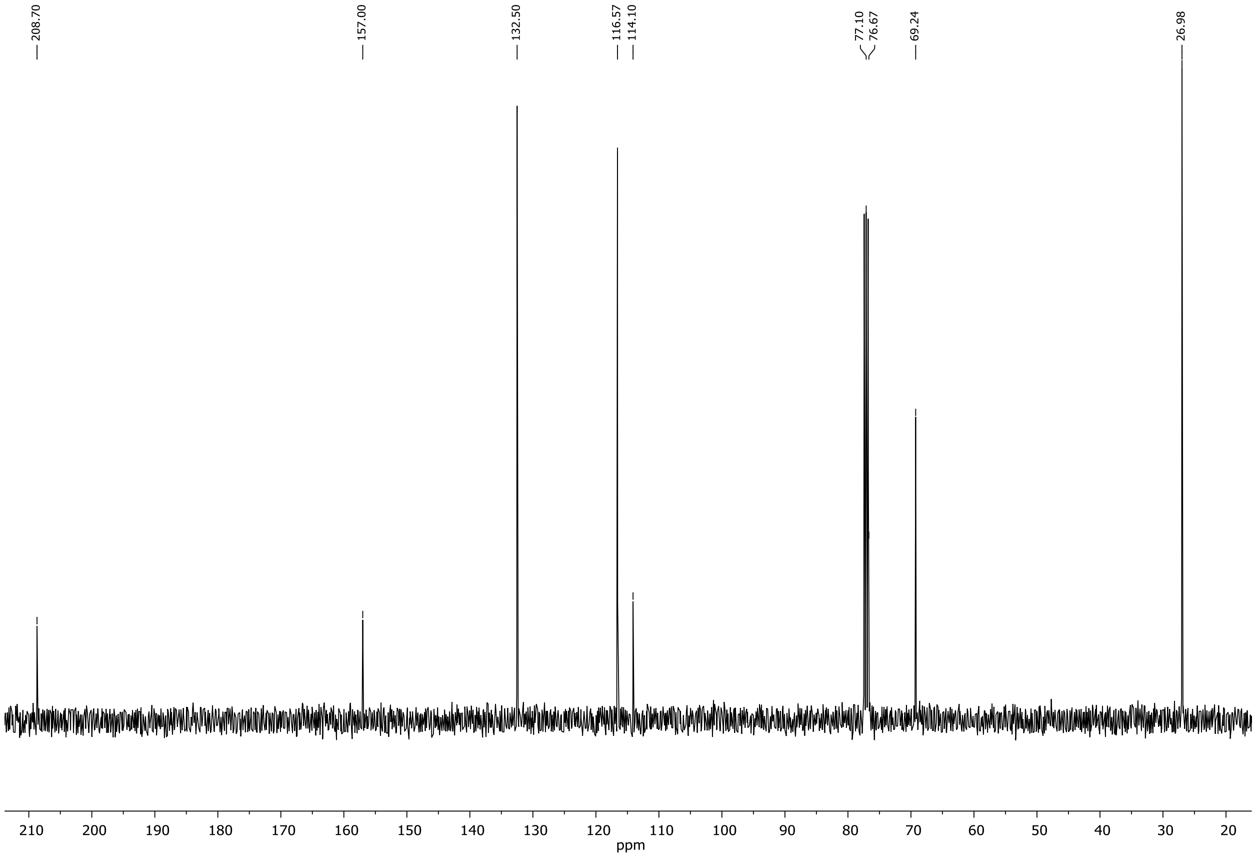

**1-(4-Allyl-2-methoxyphenoxy)-3-hydroxy-3-methylbutan-2-one (4i)**

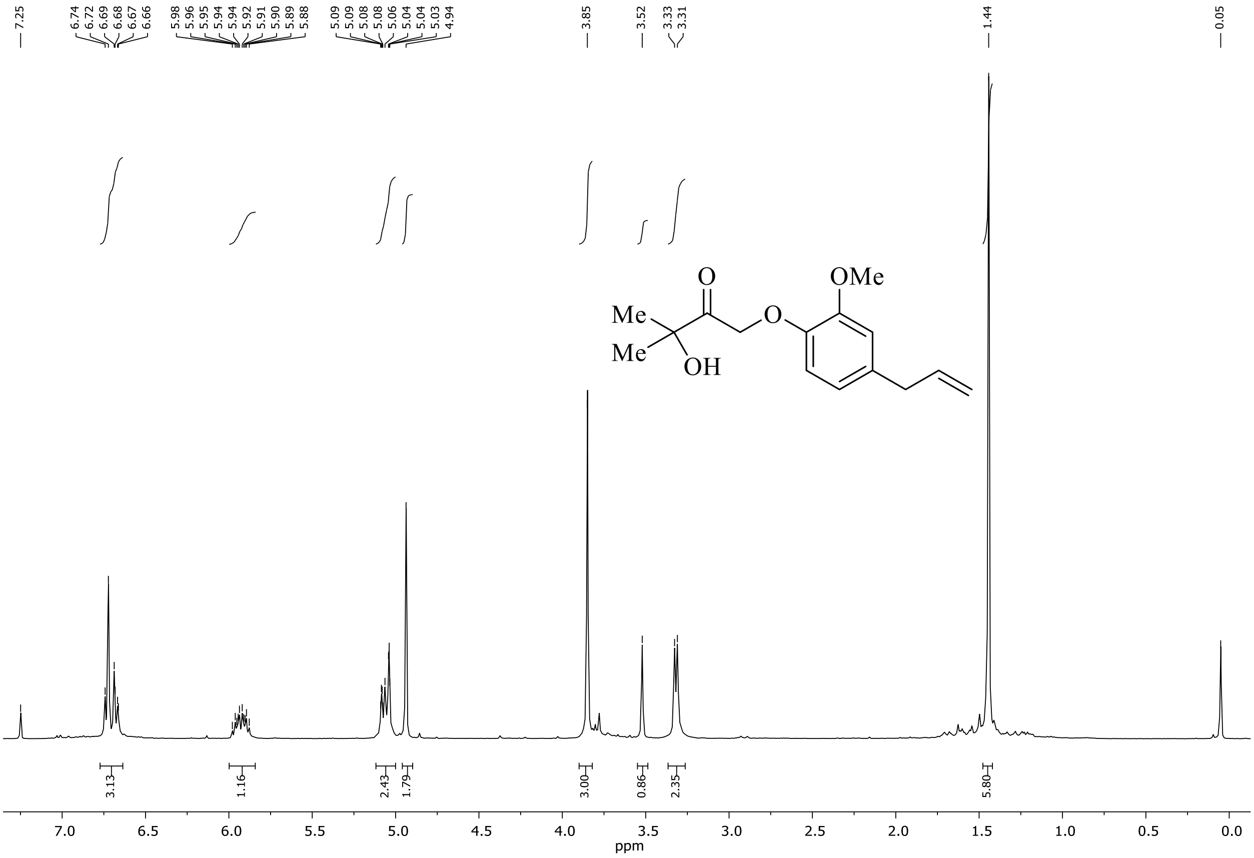


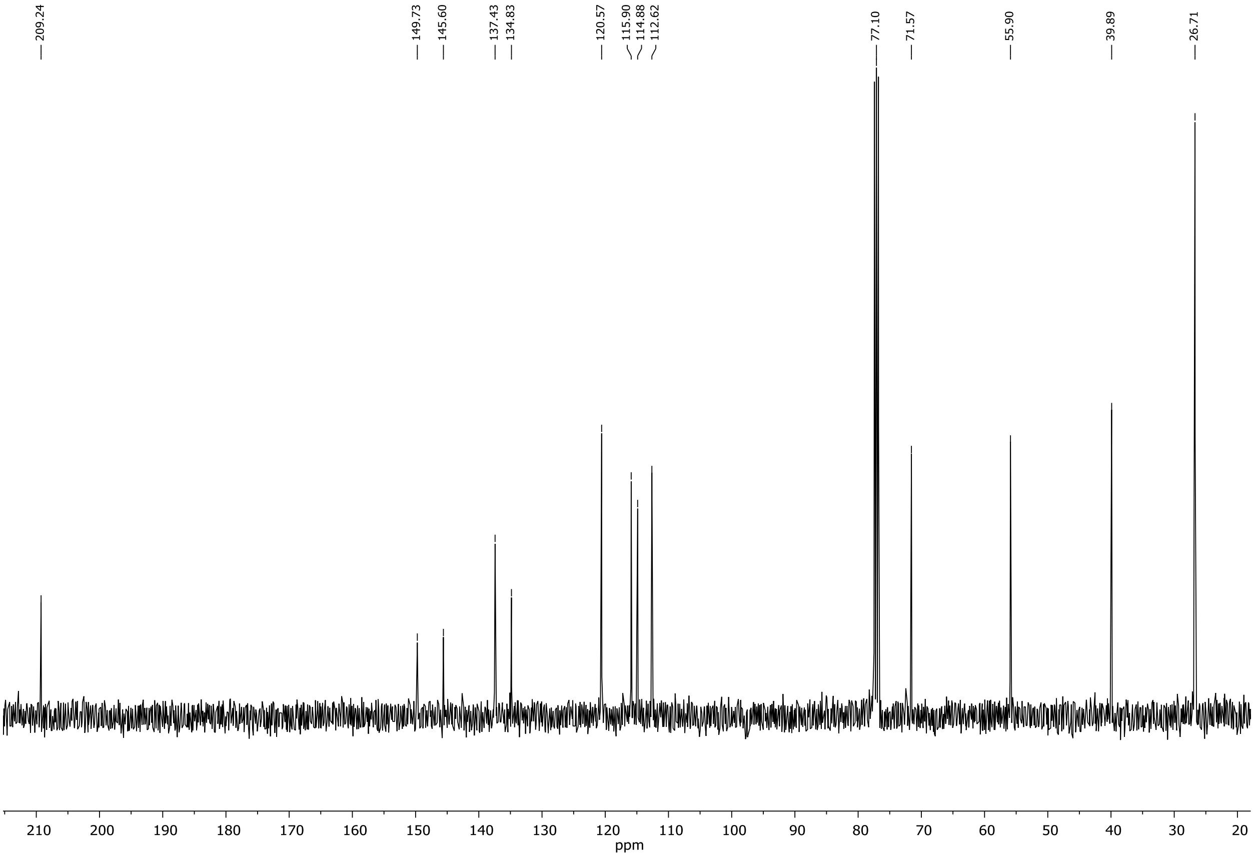

# 1-(1-Hydroxycyclohexyl)-2-phenoxyethan-1-one (4j)

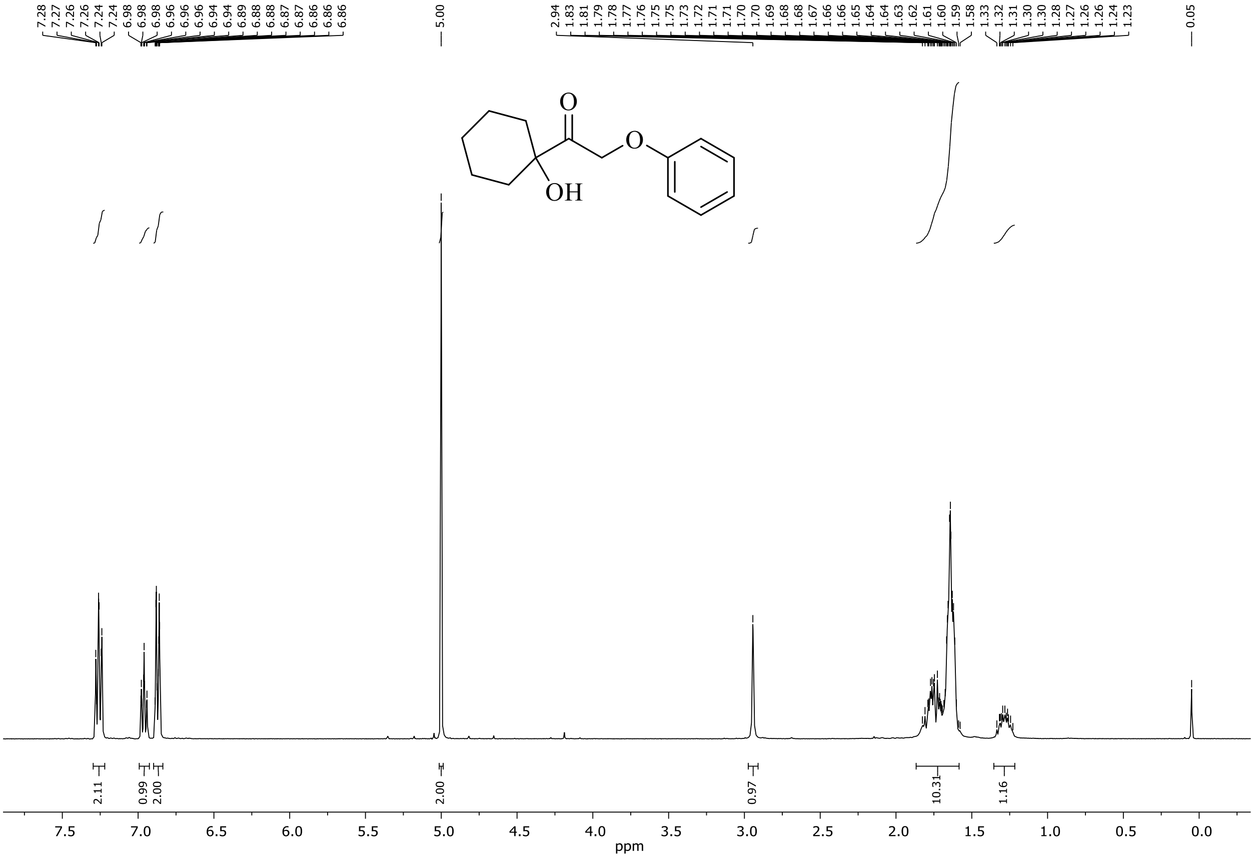


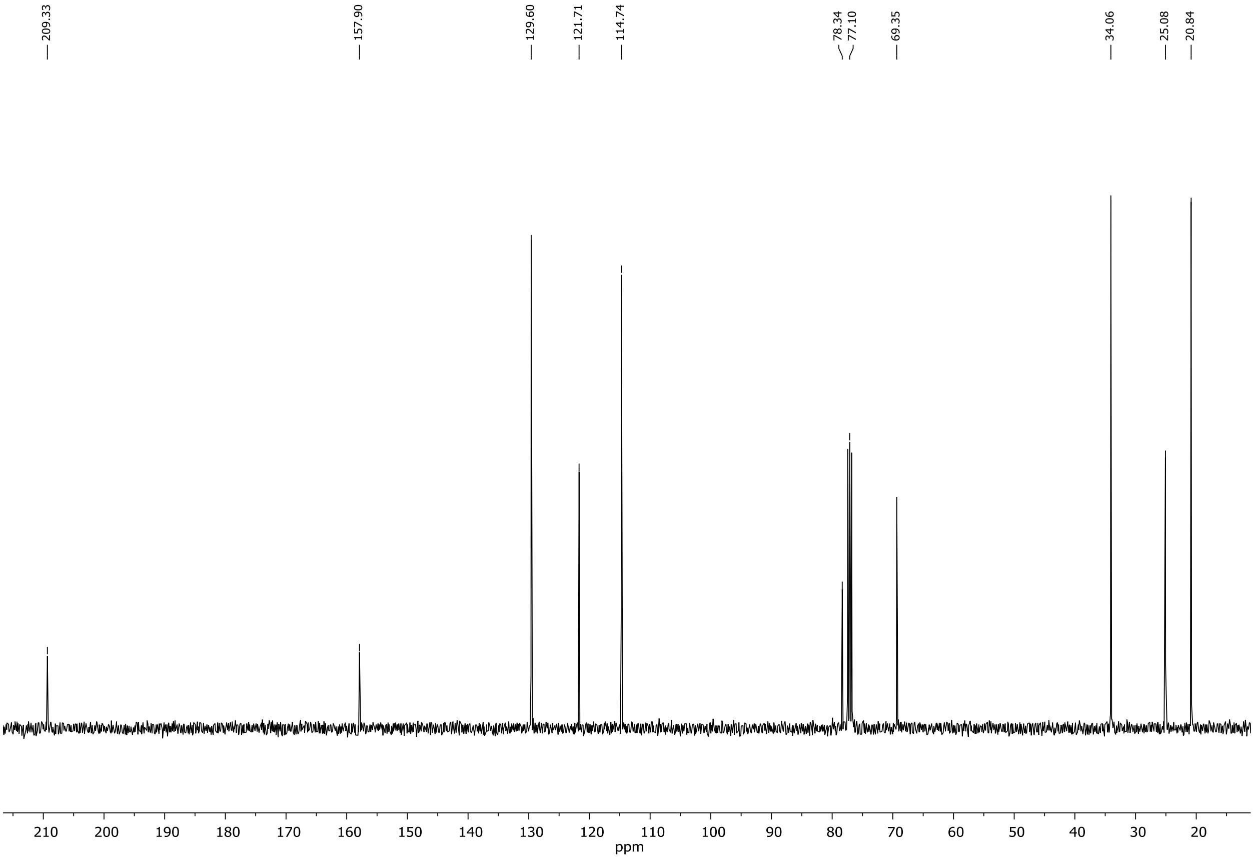

# 1-(1-Hydroxycyclohexyl)-2-(4-nitrophenoxy)ethan-1-one (4k)

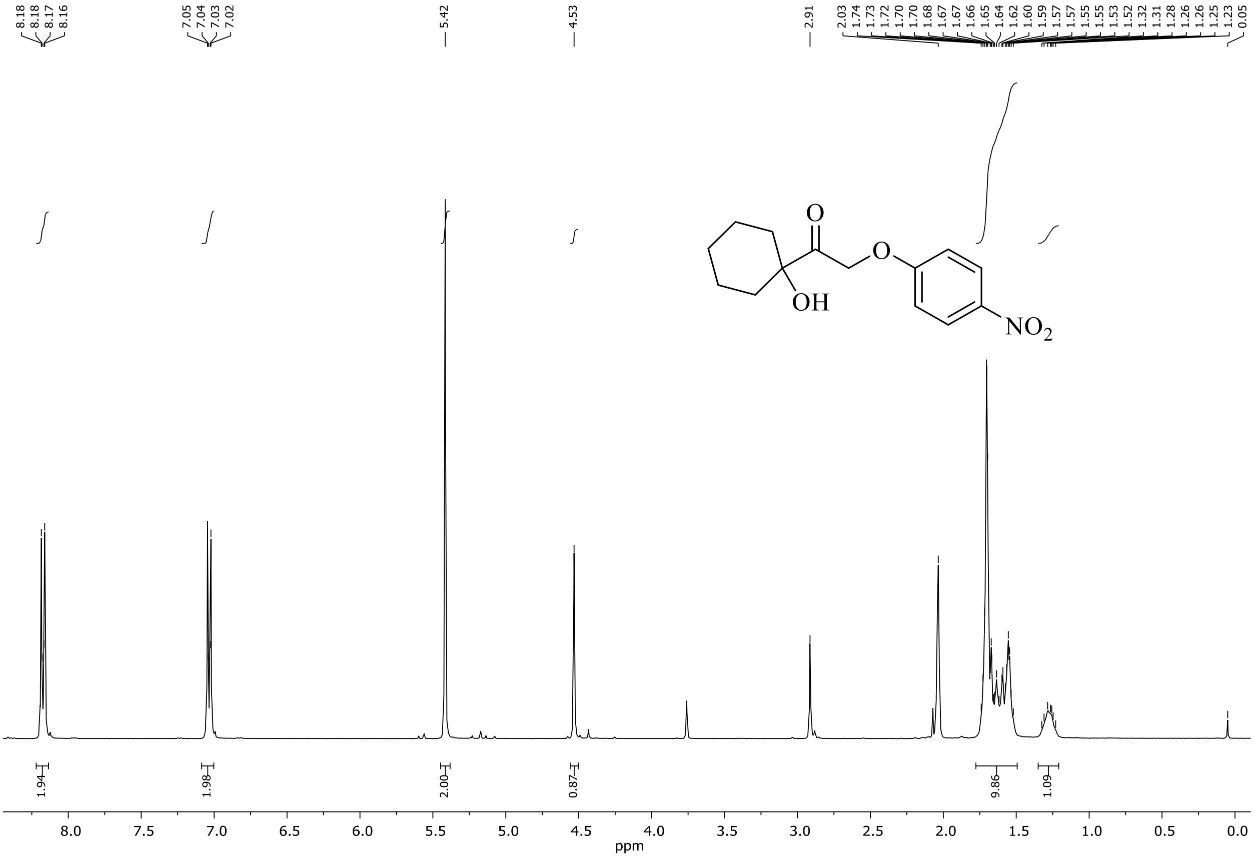


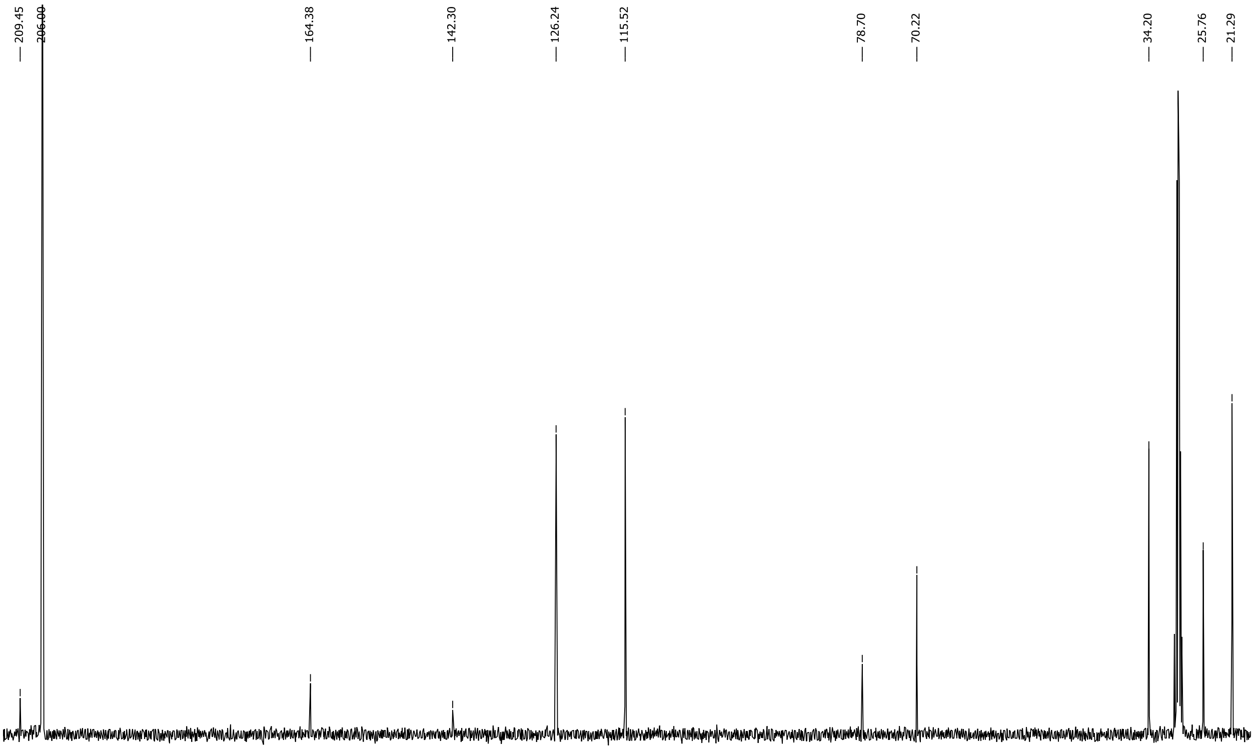

**3-Hydroxy-3,4,4-trimethyl-1-phenoxypentan-2-one (4l)**

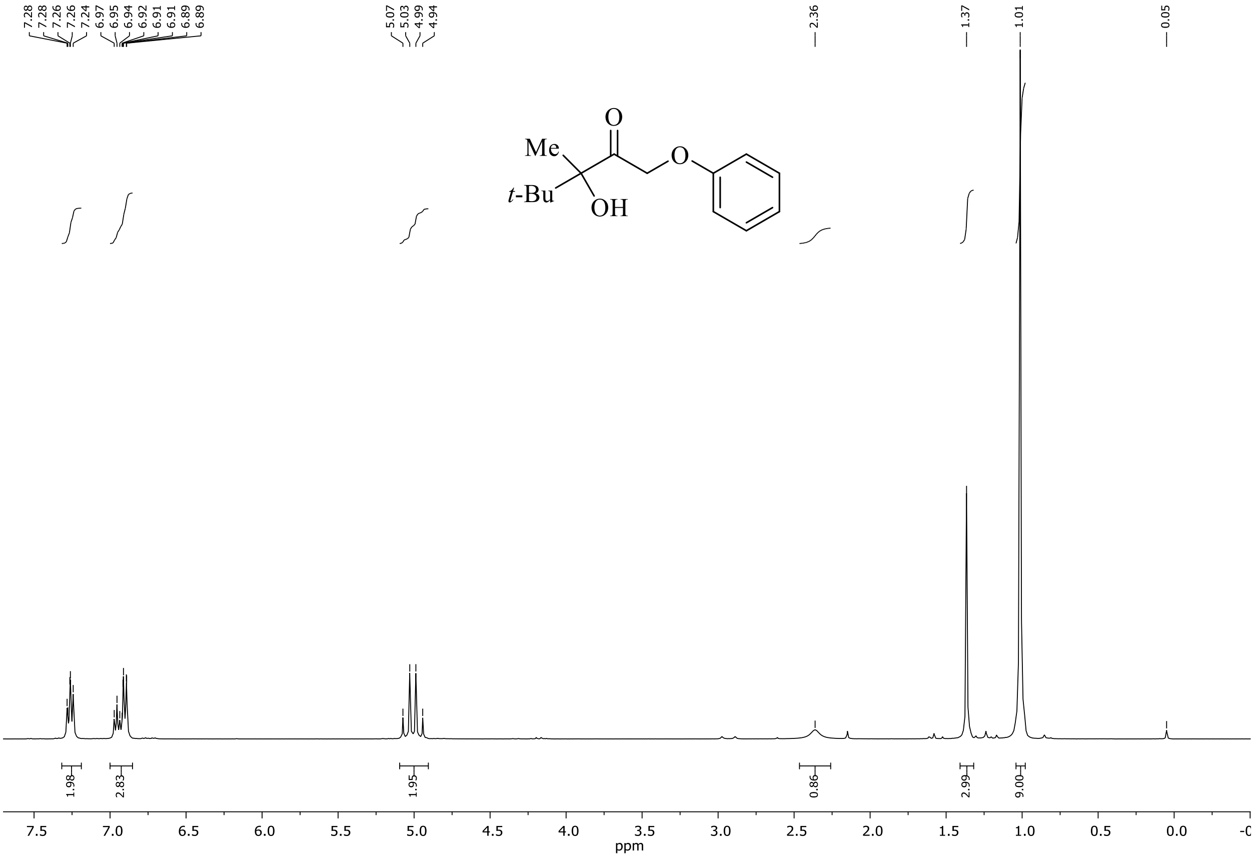

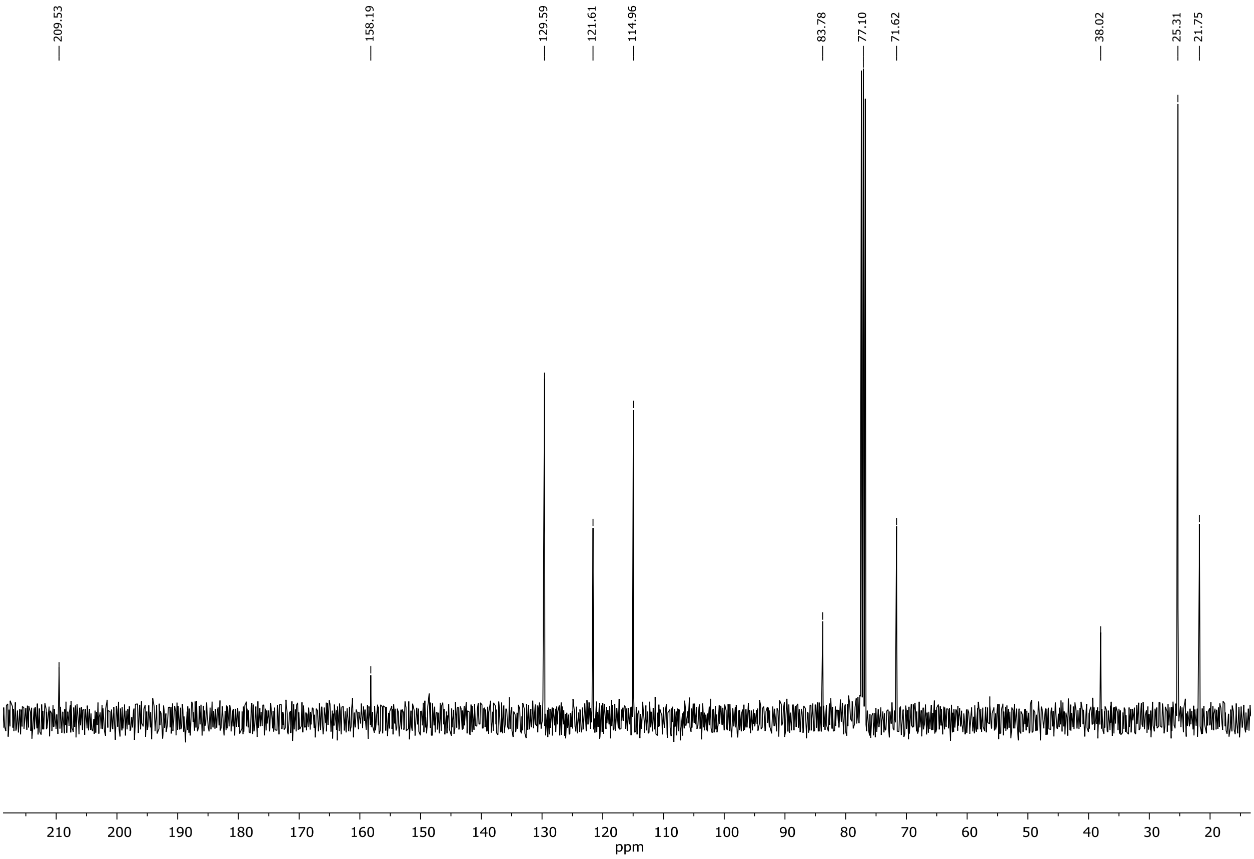

# 3-Methyl-1,1-diphenoxybutan-2-one (5a)

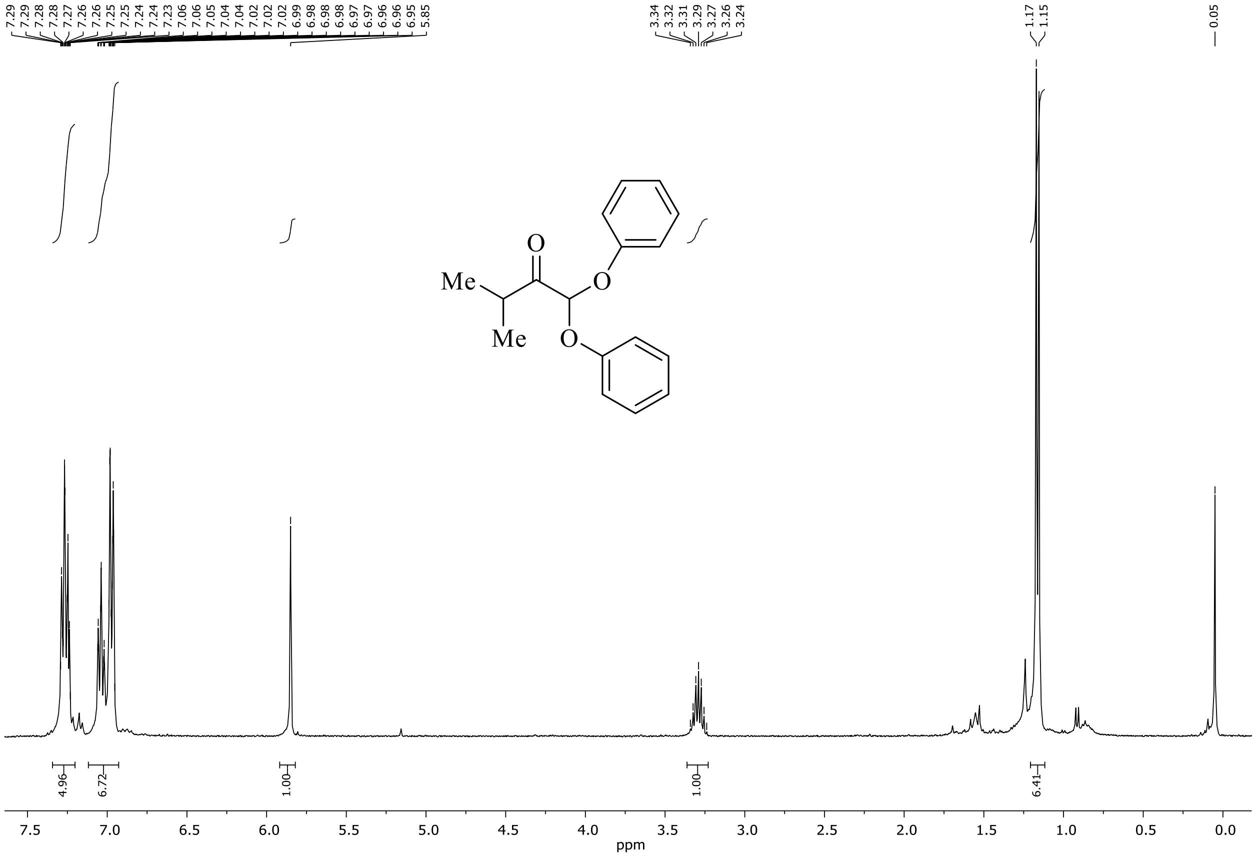


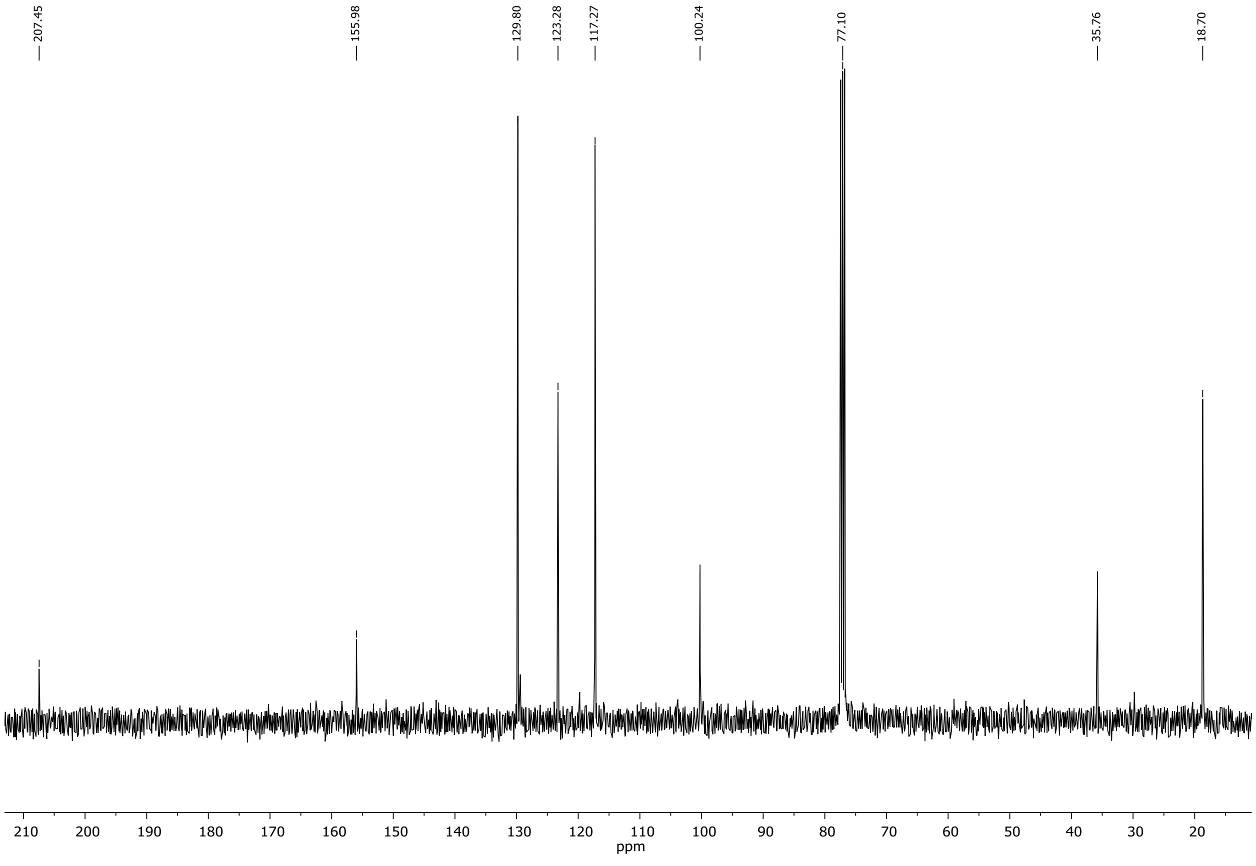

# 1,1-Bis(4-methoxyphenoxy)-3-methylbutan-2-one (5e)

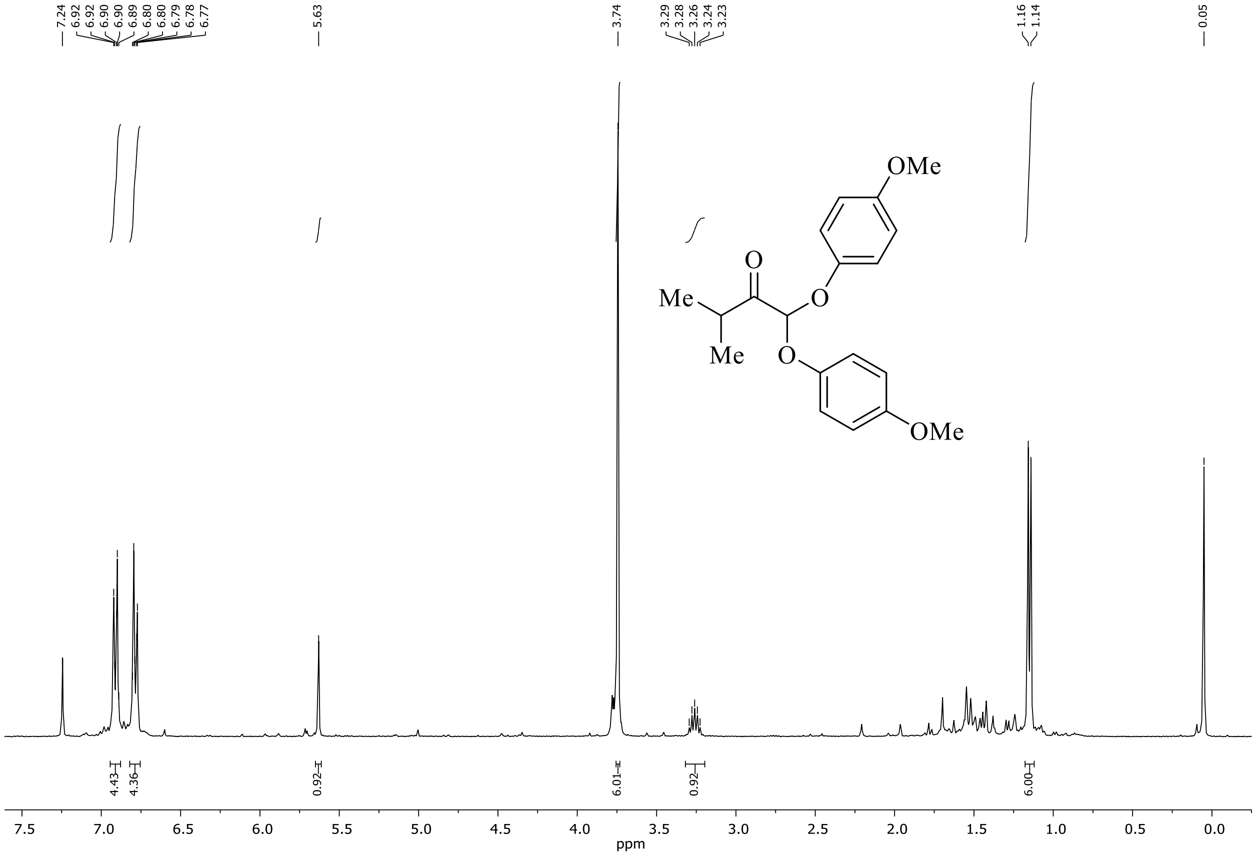


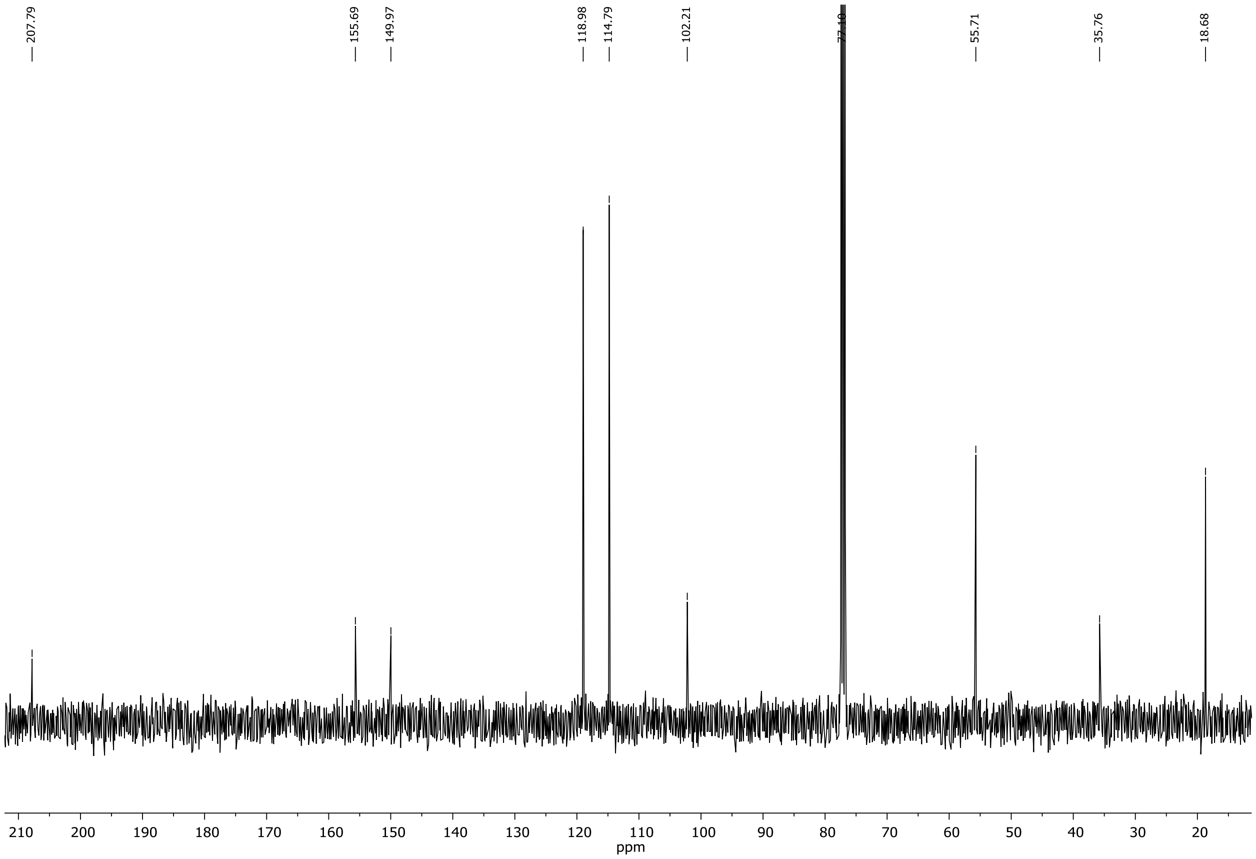

# 1,1-Bis(4-bromophenoxy)-3-methylbutan-2-one (5f)

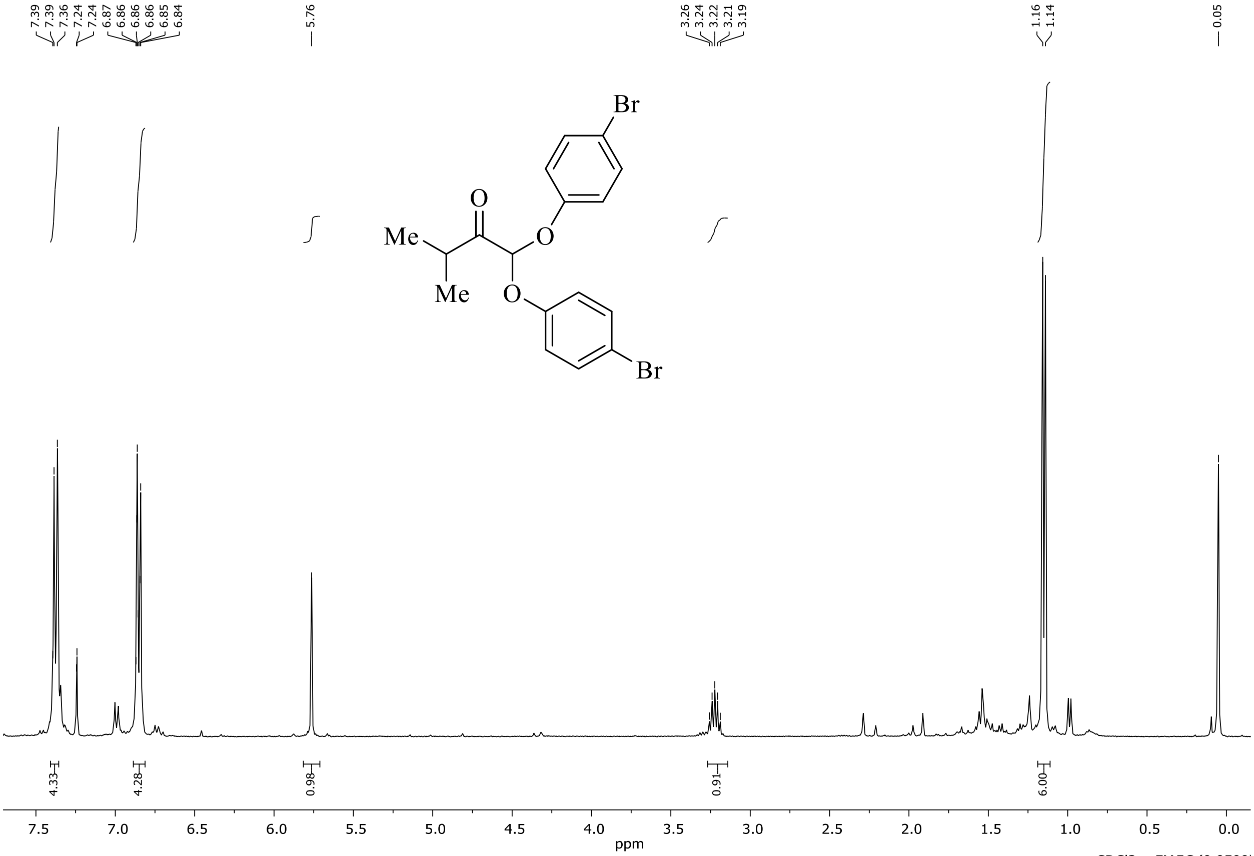


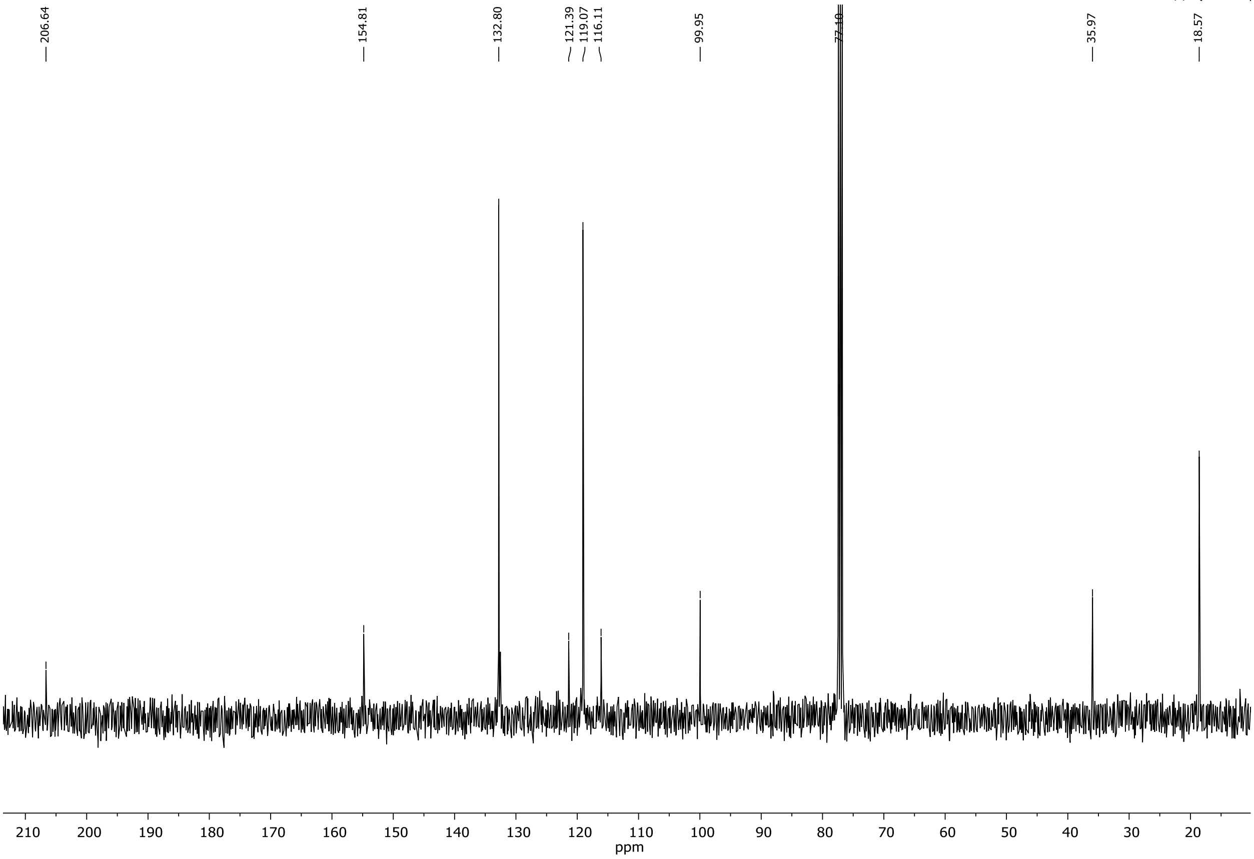

**1,1-Bis(4-allyl-2-methoxyphenoxy)-3-methylbutan-2-one (5g)**

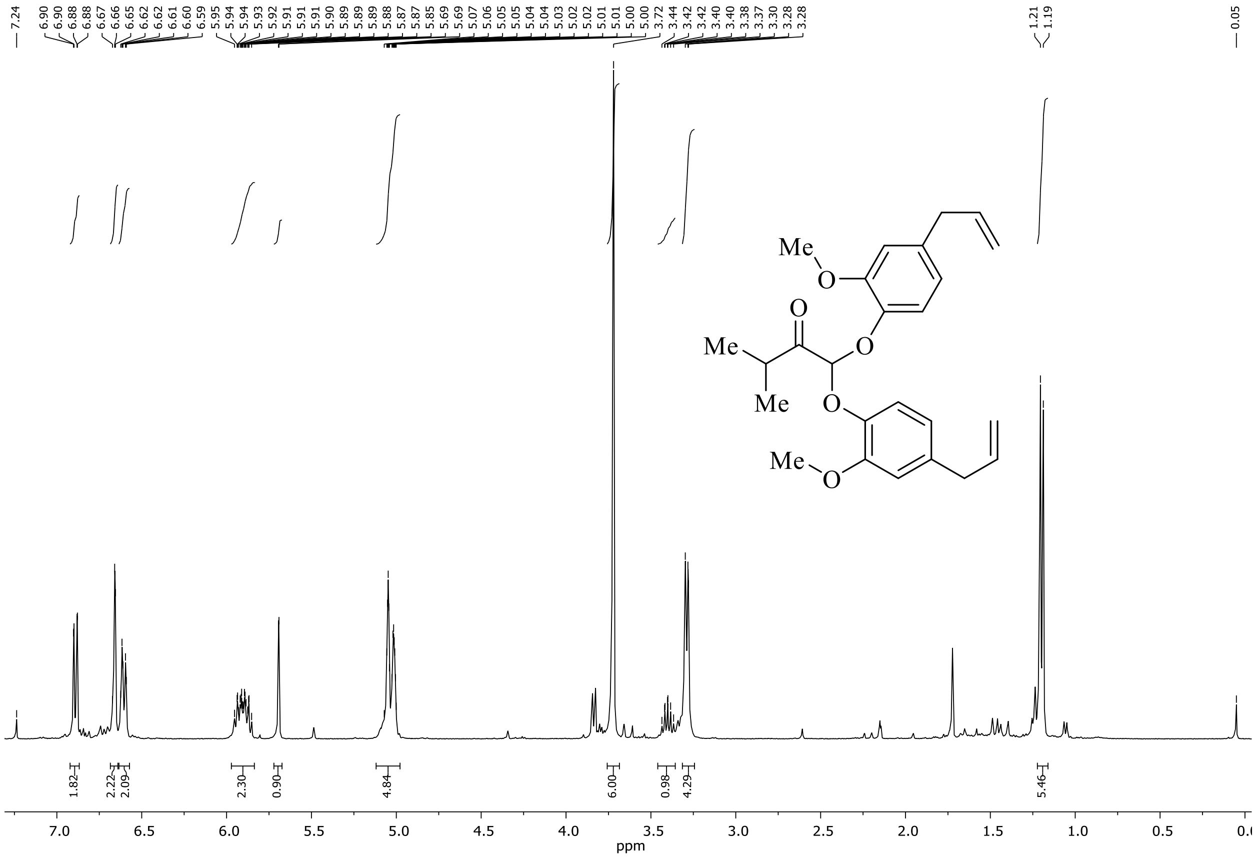

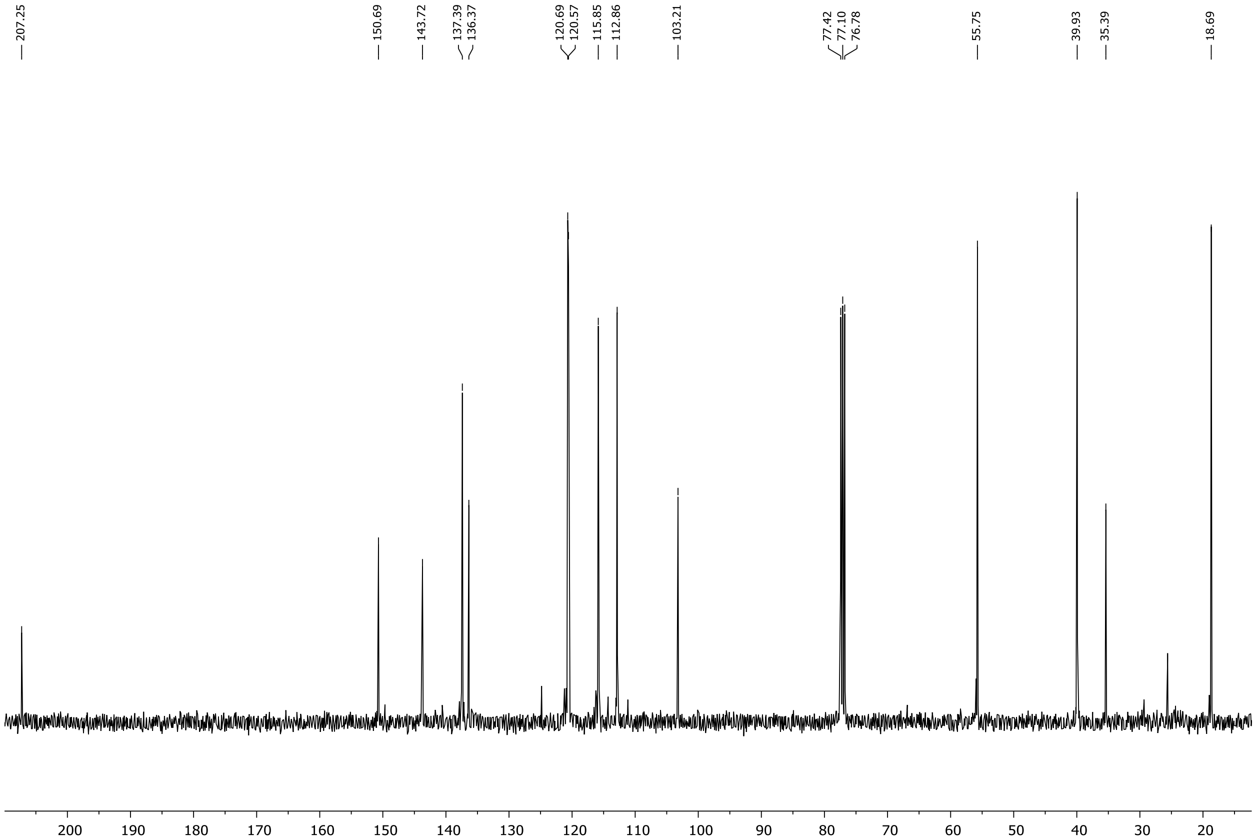

## (Z)-4,4-Dimethyl-5-(phenoxymethylene)-1,3-dioxolan-2-one (7)

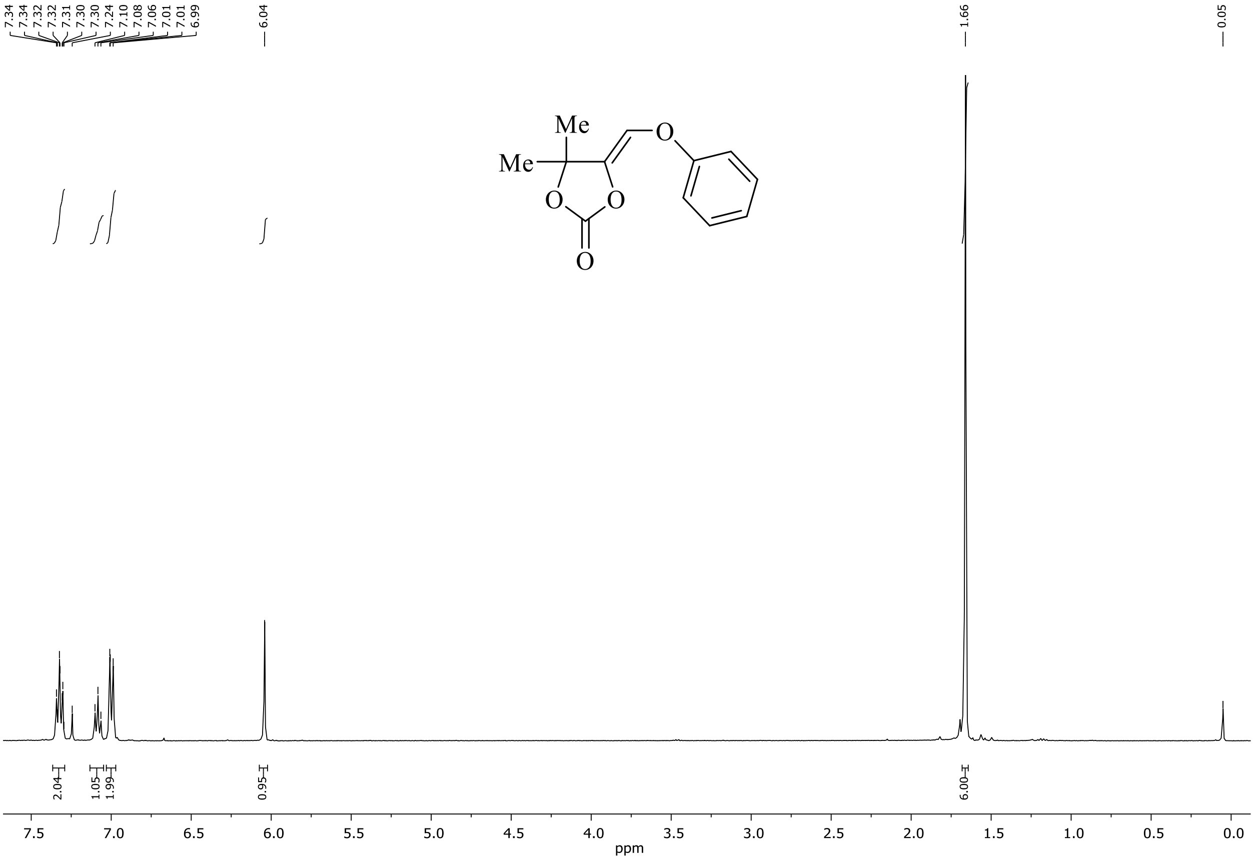


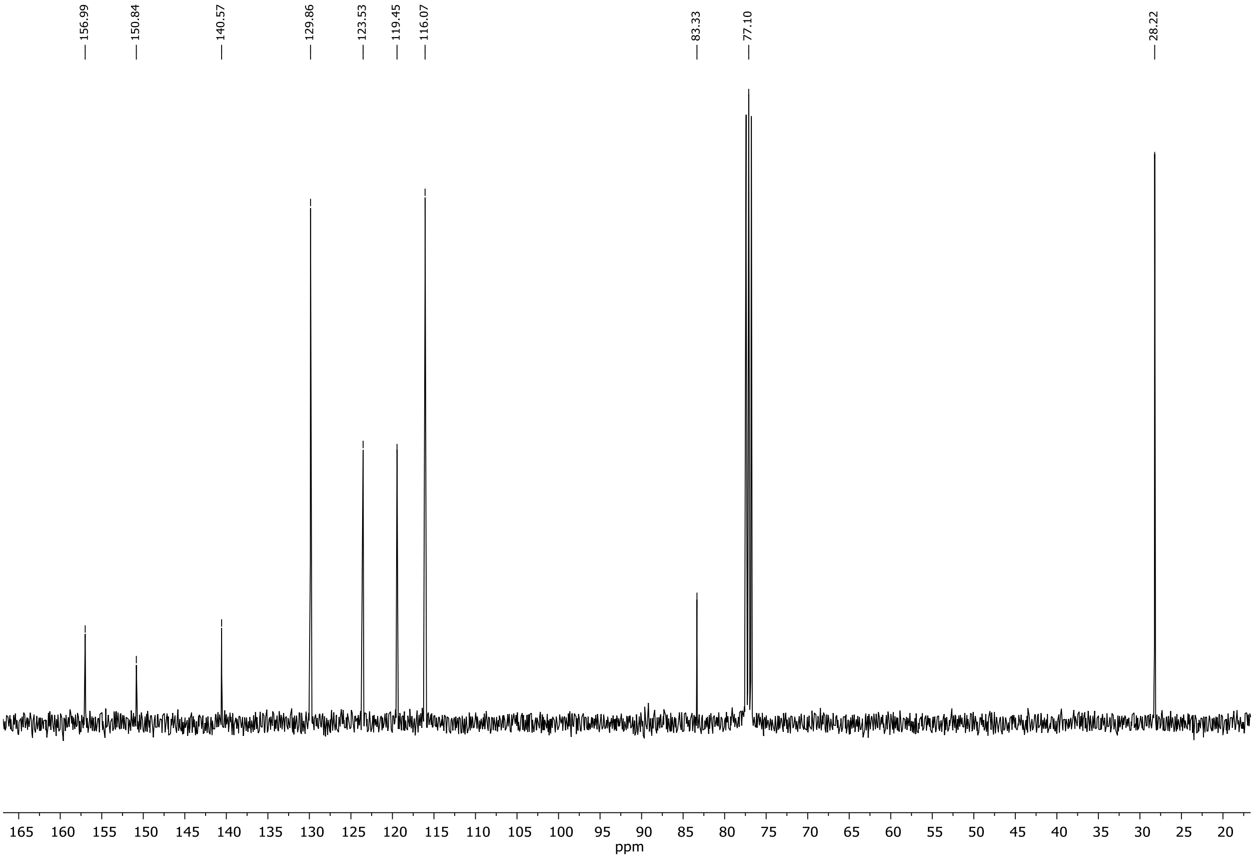

**2D $^{1}H$-$^{13}C$ HMBC Spectrum of 7 ($CDCl_3$)**

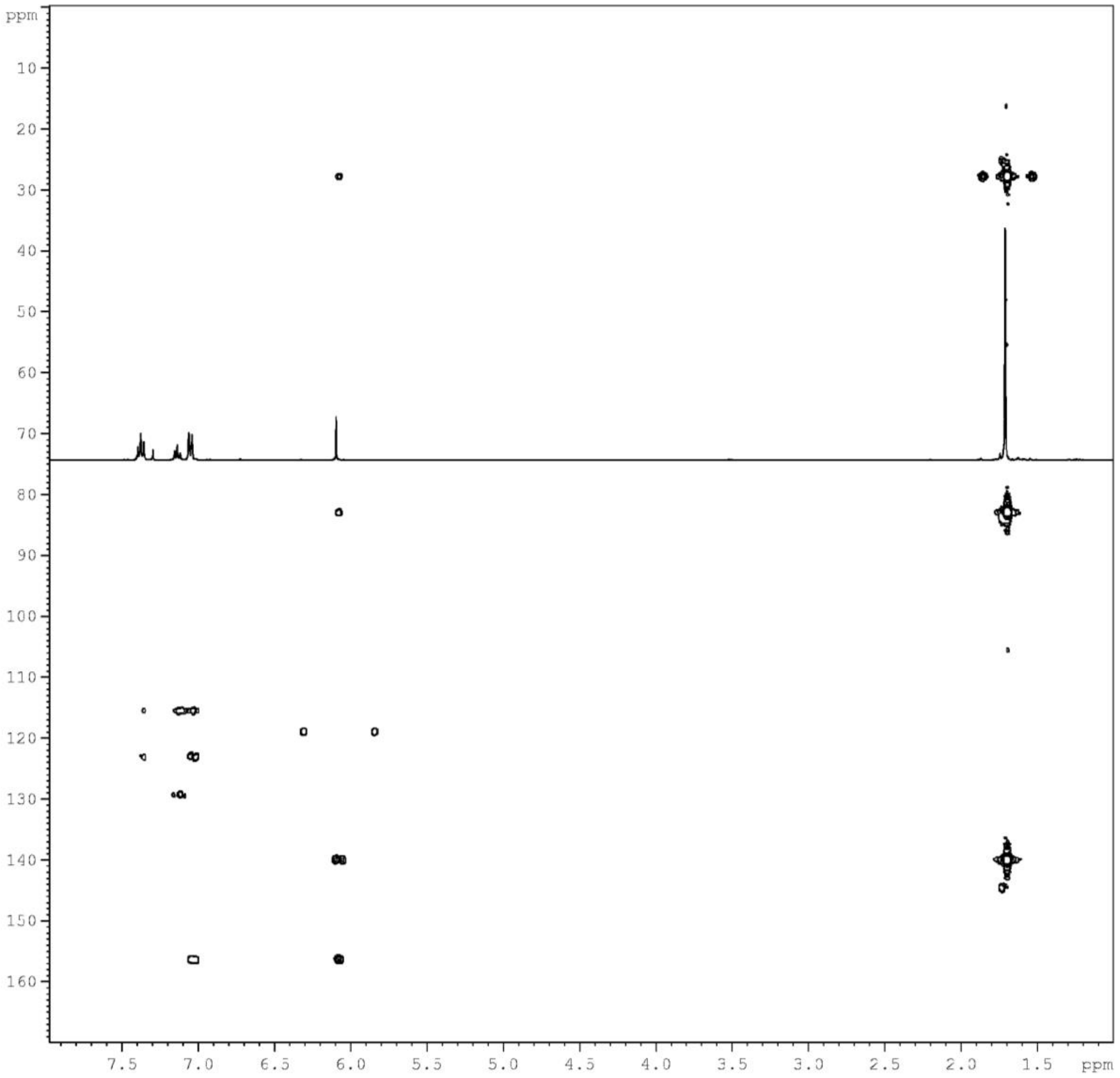

**2D NOESY Spectrum of 7 ($CDCl_3$)**

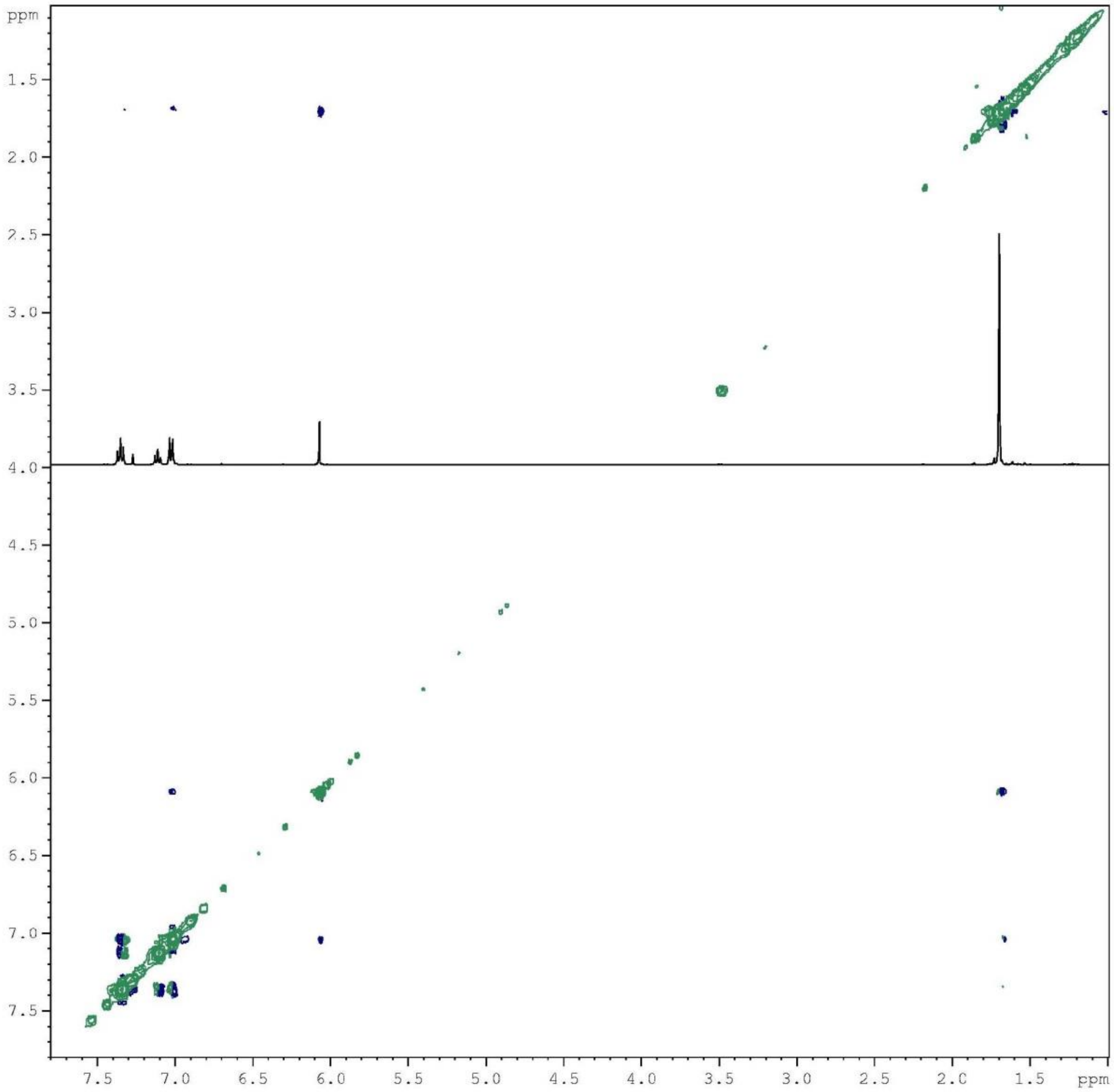

## 3-Hydroxy-3-methyl-4-phenoxybutan-2-one (9a)

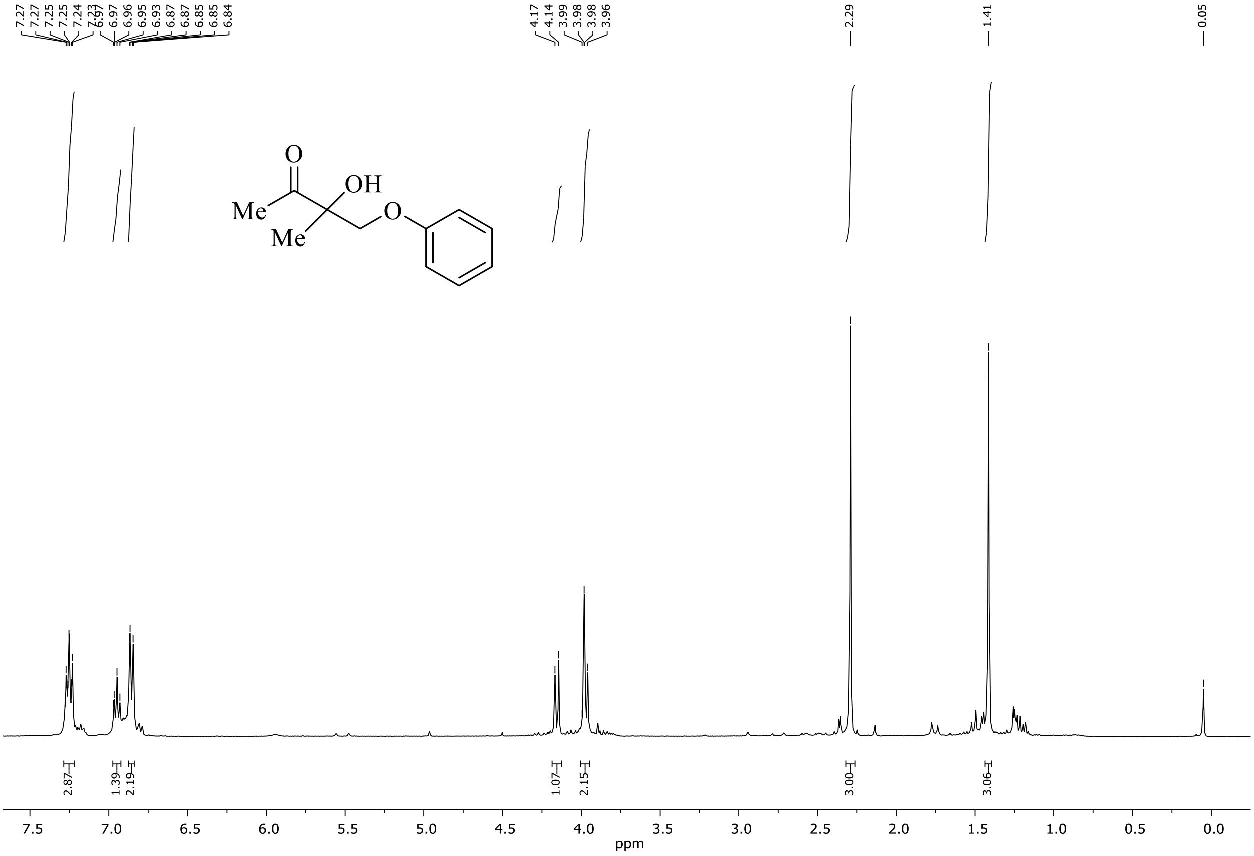

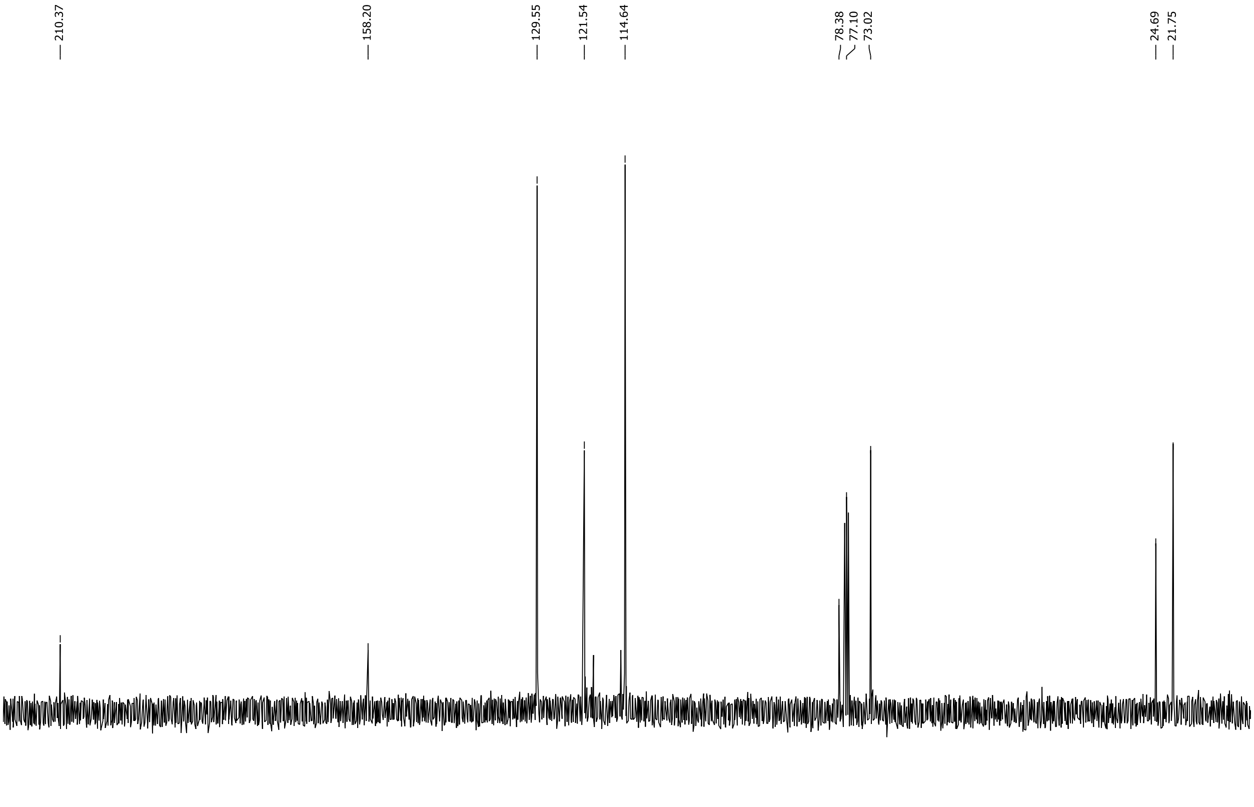

## 3-Hydroxy-3-methyl-4-(*p*-tolyloxy)butan-2-one (9b)

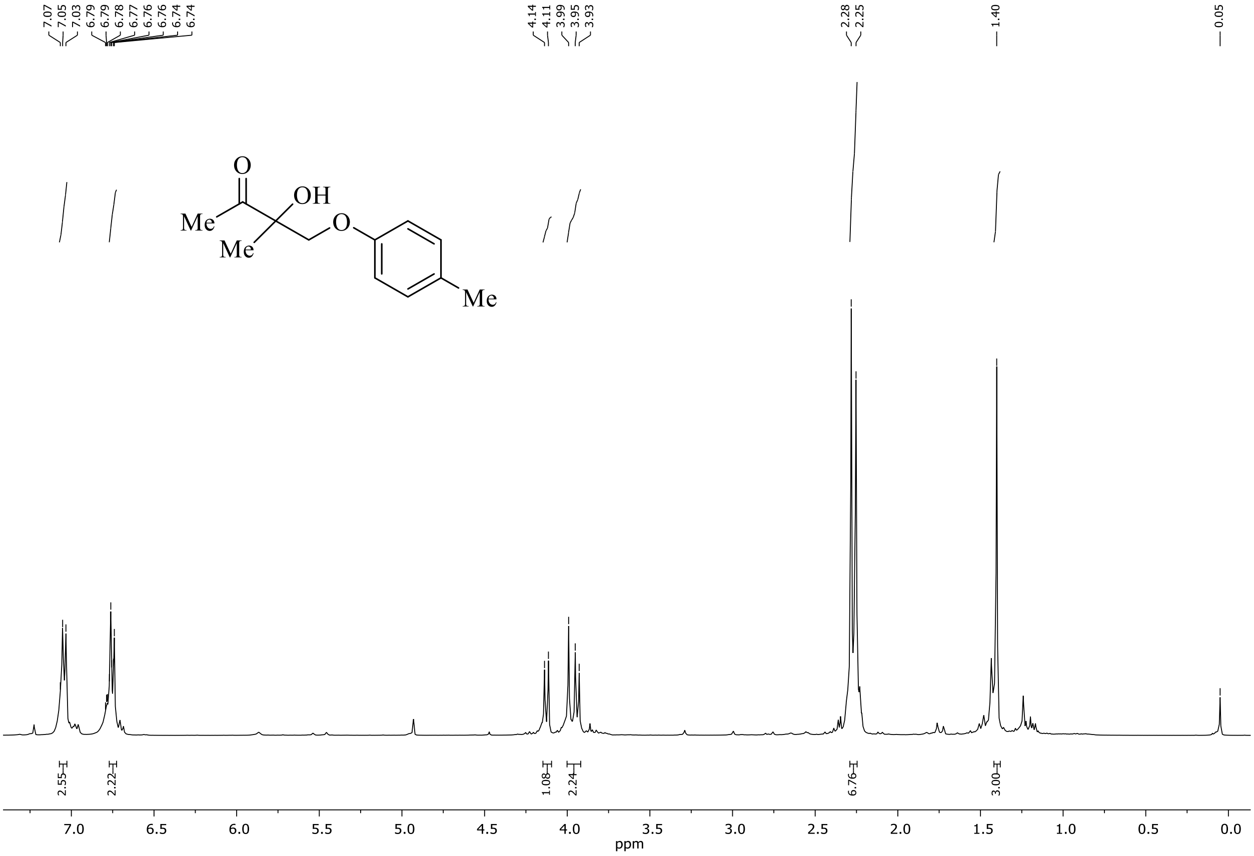


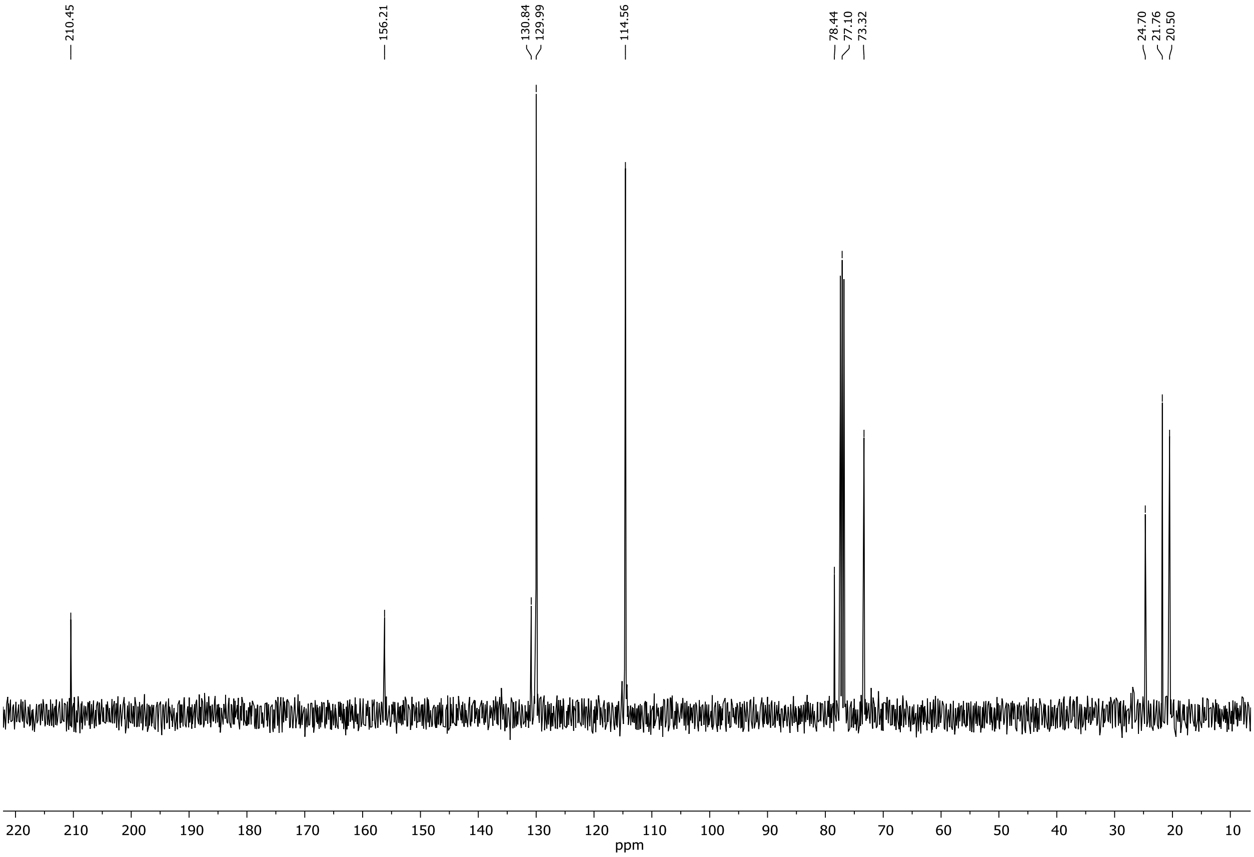

Supplement: File 1 — General information, synthetic procedures and additional optimization results, NMR spectra and characterization of synthesized compounds. [file Beilstein_J_Org_Chem-18-420-s001.pdf]
